# Hepatic NMNAT1 is required to defend against alcohol-associated fatty liver disease

**DOI:** 10.1126/sciadv.adt6195

**Published:** 2025-06-27

**Authors:** Qinchao Ding, Feiwei Cao, Hui Zhuge, Shanglei Lai, Wenjing Cao, Haibin Wei, Rui Guo, Jiannan Qiu, Qing Song, Liuhua Pei, Chaolan Li, Caijuan Si, Zhaoli Sun, Zhenyuan Song, Xiaobing Dou, Songtao Li

**Affiliations:** ^1^ School of Public Health, Zhejiang Chinese Medical University, Hangzhou, Zhejiang, PR. China.; ^2^ School of Life Science, Zhejiang Chinese Medical University, Hangzhou, Zhejiang, PR. China.; ^3^ Department of Kinesiology and Nutrition, University of Illinois at Chicago, Chicago, IL, USA.; ^4^ Department of Clinical Nutrition, Affiliated Zhejiang Hospital, School of Medicine, Zhejiang University, Hangzhou, Zhejiang, PR. China.; ^5^ Department of Surgery, Johns Hopkins University School of Medicine, Baltimore, MD, USA.

## Abstract

Nicotinamide mononucleotide adenylyltransferase 1 (NMNAT1), a nicotinamide adenine dinucleotide (NAD^+^) synthetase in Preiss-Handler and salvage pathways, governs nuclear NAD^+^ homeostasis. This study investigated the role of NMNAT1 in alcohol-associated liver disease (ALD). Decreased NMNAT1 expression and activity were observed in the liver of patients with alcohol-associated hepatitis and either liver or primary hepatocytes from ALD mice. F-box and WD repeat domain containing 7 (FBXW7)–regulated interferon regulatory factor 1 (IRF1) ubiquitination degradation contributed to the alcohol-inhibited NMNAT1 transcriptional level. Hepatic NMNAT1 knockout aggravated alcohol-induced hepatic NAD^+^ decline and further hepatic steatosis and liver injury. Metabolomics and transcriptomics interaction revealed that the cysteine sulfinic acid decarboxylase (CSAD)–regulated taurine pathway was involved in NMNAT1-disrupted hepatic lipid metabolism in ALD. Hepatic CSAD overexpression or taurine supply attenuated hepatic NMNAT1 knockout–aggravated ALD. Hepatic NMNAT1 loss inhibited NMN-protected ALD. Replenishing hepatic NMNAT1 reversed liver lipid accumulation in ALD mice. These findings identified NMNAT1 as a promising therapeutic target for ALD.

## Teaser

Replenishment of hepatic NMNAT1, a nuclear NAD^+^ synthase, is critical for mitigating alcohol-associated fatty liver disease.

## INTRODUCTION

Alcohol-associated liver disease (ALD), caused by prolonged and excessive alcohol intake, stands as a predominant form of chronic liver ailment on a global scale (*1*). While early-stage ALD, characterized by hepatic steatosis, is reversible, prolonged alcohol abuse can result in progressive steatohepatitis, fibrosis, and, in severe cases, cirrhosis or hepatocellular carcinoma (*2*). Despite a considerable understanding of the disease progression and pathogenesis, the precise mechanisms driving ALD remain unclear, hampering the development of targeted therapies. Therefore, there is an urgent need to elucidate the mechanisms involved in ALD initiation and progression to facilitate the development of rational treatments.

Nicotinamide adenine dinucleotide (NAD^+^) is a pivotal metabolite essential for cellular homeostasis, particularly within hepatocytes, where it serves as a cofactor in numerous redox reactions crucial for energy metabolism, including glycolysis, tricarboxylic acid cycle, oxidative phosphorylation, and fatty acid oxidation (*3*). Decreased hepatic NAD^+^ levels are intricately linked with the progression of ALD, contributing to liver steatosis, oxidative stress, and injury (*4*, *5*). Replenishing NAD^+^ levels has shown promise in mitigating alcohol-induced hepatic steatosis, mitochondrial dysfunction, and liver damage (*6*–*8*). However, the precise mechanisms underlying alcohol-induced NAD^+^ decline in the liver remain incompletely understood. Nicotinamide mononucleotide adenylyltransferase (NMNAT) plays a central role in NAD^+^ synthesis, catalyzing its formation from nicotinic acid mononucleotide and nicotinamide mononucleotide (NMN) via the Preiss-Handler and salvage pathways, respectively (*9*). While three NMNAT isozymes (Nmnat1 to Nmnat3) exist, each with distinct subcellular localizations and functions, the specific involvement of hepatic NMNAT1 in ALD development warrants further investigation, given that NMNAT1 is the most abundant and catalytically efficient NAD^+^ synthetase governing nuclear NAD^+^ homeostasis (*10*–*12*). Whole-body deletion of the *Nmnat1* gene in vivo is embryonically lethal, suggesting its essential role in maintaining cellular NAD^+^ levels and homeostasis (*13*). However, the relevance and mechanisms of hepatic NMNAT1 and nuclear NAD^+^ in ALD pathogenesis remain largely elusive.

In the present study, we found that hepatic NMNAT1 and nuclear NAD^+^ were significantly decreased in the liver of both patients with alcohol-associated hepatitis (AH) and ALD mice, and this was transcriptionally regulated by alcohol-induced interferon regulatory factor 1 (IRF1) ubiquitination degradation. Hepatic NMNAT1 null mice exacerbated alcohol-induced hepatic steatosis and liver injury by inhibiting the cysteine sulfinic acid decarboxylase (CSAD)–regulated taurine pathway in a NAD^+^-dependent manner. Our findings demonstrated that NMNAT1 is a promising therapeutic target for ALD treatment.

## RESULTS

### Hepatic NMNAT1 is suppressed in both humans and mice with ALD

**Fig. 1.**
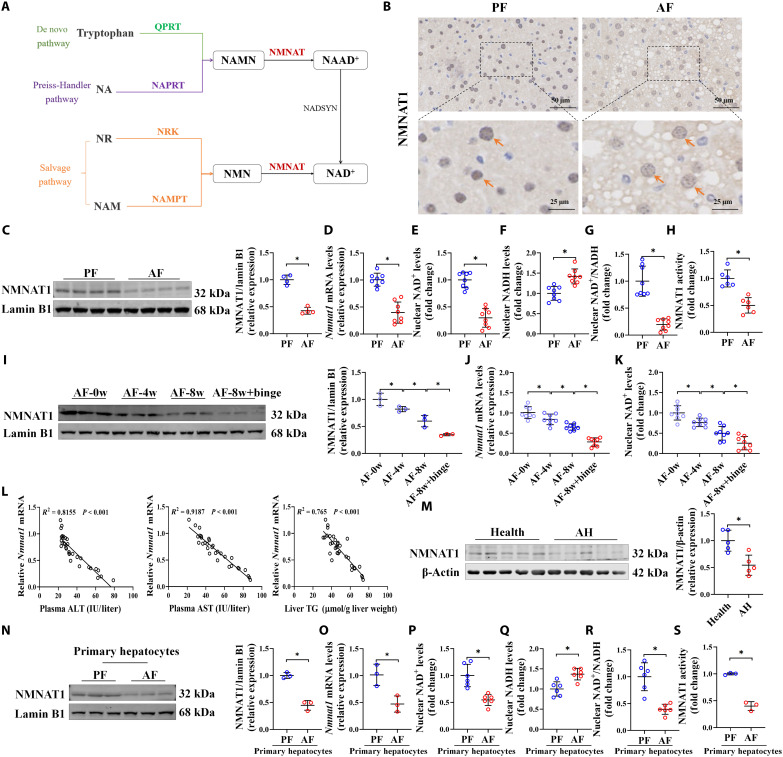
Hepatic NMNAT1 is suppressed in both humans and mice with ALD. (**A**) Schematic diagram of the NAD^+^ synthesis pathway. (**B** to **H**) Representative immunohistochemical images of hepatic NMANT1 (B), NMNAT1 protein expression (C), *Nmnat1* mRNA expression (D), nuclear NAD^+^ content (E), nuclear NADH content (F), nuclear NAD^+^/NADH ratio (G), and NMNAT1 activity (H) were obtained in the Lieber-DeCarli diet plus single binge model (*n* = 4 to 8). (**I** to **K**) Liver NMNAT1 protein expression (I), mRNA expression (J), and nuclear NAD^+^ content (K) were detected at different stages of Lieber-DeCarli alcohol feeding (*n* = 4 to 8). (**L**) Correlation analysis of *Nmnat1* mRNA expression with plasma ALT and AST and liver TG (*n* = 32). (**M**) NMNAT1 protein expression in patients with AH (*n* = 5) and healthy individuals (*n* = 5). (**N** to **S**) Primary hepatocyte nuclei were isolated from the livers of Lieber-DeCarli diet–fed PF and AF mice, and NMNAT1 protein expression (N) and mRNA expression (O), as well as NAD^+^ (P), NADH (Q), NAD^+^/NADH ratio (R), and NMNAT1 activity (S), were detected (*n* = 3). The protein band intensity was quantified by ImageJ. Data are presented as the means ± SD. *P < 0.05 represents statistical difference.

NMNAT1 is crucial for the generation of nuclear NAD^+^ from either nicotinic acid mononucleotide in the Preiss-Handler pathway or NMN in the salvage pathway (Fig. 1A). To test the role of NMNAT1 in ALD development, a commonly used ALD model established by feeding C57BL/6N mice with a Lieber-DeCarli liquid ethanol diet and a single binge or an isocaloric pair-fed (PF) diet was used in this study (fig. S1); compared to the PF group, alcohol-fed (AF) diet led to a significant decrease in hepatic NMNAT1 expression at both mRNA and protein levels, along with decreased nuclear NAD^+^ levels, NAD^+^/NADH (reduced form of NAD^+^) ratio, and NMNAT1 activity (Fig. 1, B to H, and figs. S1 and S2). Alcohol-decreased NMNAT1 exhibited a time-course manner, concomitant with a corresponding abatement of nuclear NAD^+^ content (Fig. 1, I to K, and fig. S3). The characteristic pathological indicators of ALD, including plasma alanine transaminase (ALT), aspartate transaminase (AST), and liver triglyceride (TG) levels, are significantly negatively associated with the protein abundance of hepatic NMNAT1 (Fig. 1L). Hepatic NMNAT1 was also robustly decreased in the CCl_4_-induced alcohol-associated liver fibrosis model (fig. S4). To validate the clinical relevance of our findings in mice, NMNAT1 expression in liver samples obtained from patients with AH and healthy individuals was detected. As shown in Fig. 1M, hepatic NMNAT1 protein expression was significantly decreased in patients with AH. In addition, NMNAT1 expression was down-regulated in primary hepatocytes isolated from the livers of chronic AF mice, accompanied by decreased nuclear NAD^+^ levels, NAD^+^/NADH ratio, and NMNAT1 activity (Fig. 1, N to S). In vitro, decreased NMNAT1 was observed in mouse primary hepatocytes, AML-12 mouse hepatocytes, and human VL-17A hepatocytes in response to lipotoxicity, acetaldehyde, and hydrogen peroxide exposure (fig. S5); however, this phenomenon was not observed in hepatocytes directly exposed to ethanol (fig. S5).

### Alcohol-stimulated IRF1 ubiquitination degradation contributes to an NMNAT1 decrease

**Fig. 2.**
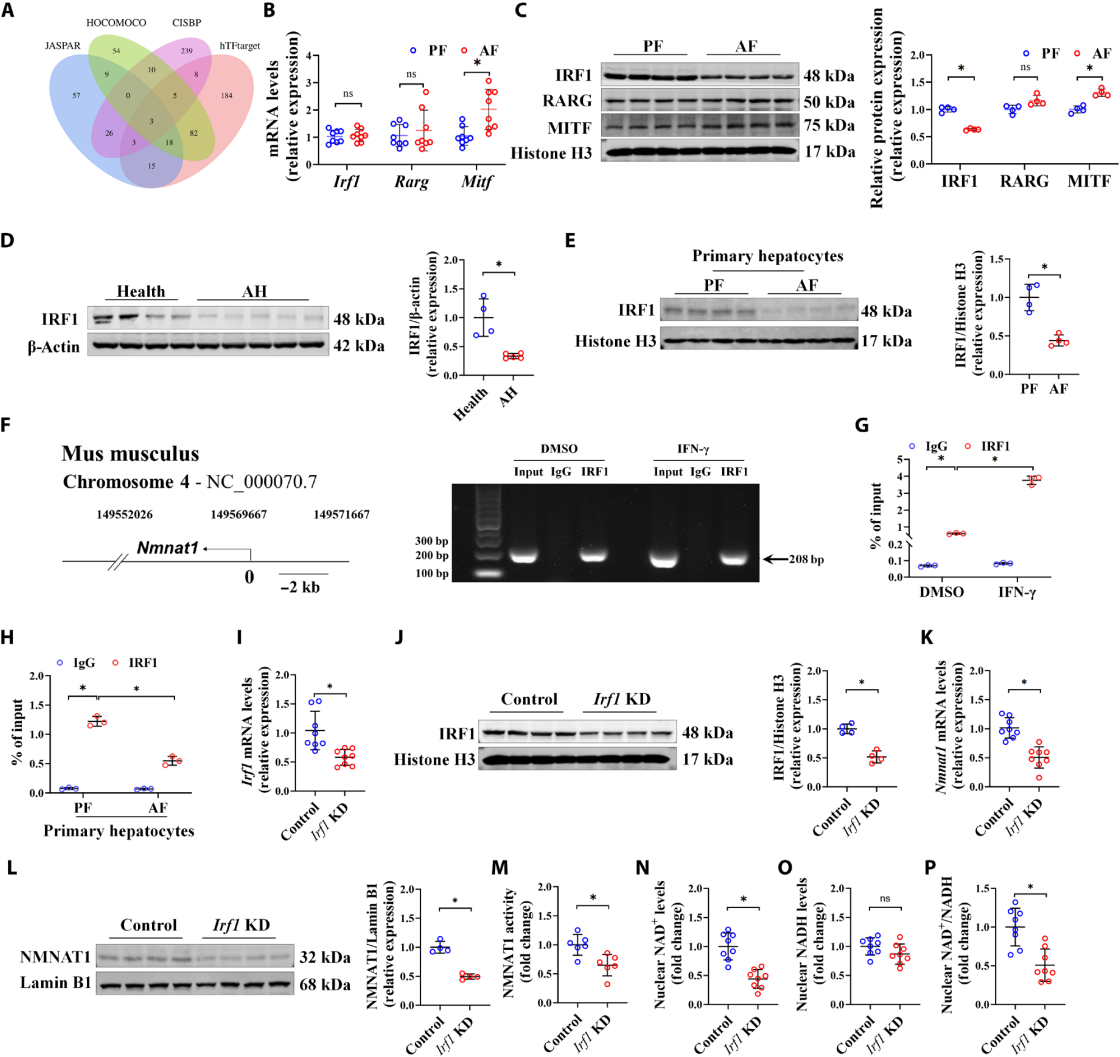
Alcohol-stimulated IRF1 ubiquitination degradation contributes to NMNAT1 reduction. (**A**) Venn plot of nuclear transcription factor prediction for NMNAT1. (**B**) Relative expression of *Irf1*, *Rarg*, and *Mitf* in the AF mouse liver (*n* = 8). (**C**) IRF1, RARG, and MITF protein expressions in the AF mouse liver (*n* = 4). (**D**) IRF1 protein expression in patients with AH (*n* = 5) and healthy individuals (*n* = 4). (**E**) Primary hepatocyte nuclei were isolated from the livers of Lieber-DeCarli diet–fed PF and AF mice. IRF1 protein expression was detected (*n* = 4). (**F** and **G**) AML-12 cells were treated with/without IFN-γ (1 ng/ml) for 6 hours, and IRF1 recruitment was determined by ChIP assay. DNA was extracted from the immunoprecipitates, and a fragment of the ribosomal DNA (rDNA) promoter sequence was amplified by real-time qPCR. PCR products were analyzed by 2% agarose gel electrophoresis (F). The rDNA levels were quantified by real-time qPCR [(G); *n* = 3]. IgG, immunoglobulin G. (**H**) Primary hepatocytes isolated from PF and AF mouse livers were used to detect the recruitment of IRF1 on the promoter sequence of NMNAT1 by ChIP assay. The rDNA levels were quantified by real-time qPCR (*n* = 3). (**I** to **P**) Hepatocyte-specific IRF1 knockdown mice were generated as described in Materials and Methods. Liver *Irf1* mRNA expression (I), IRF1 protein expression (J), *Nmnat1* mRNA expression (K), NMNAT1 protein expression (L), NMNAT1 activity (M), nuclear NAD^+^ content (N), nuclear NADH content (O), and nuclear NAD^+^/NADH ratio (P) were detected (*n* = 4 to 8). The protein band intensity was quantified by ImageJ. Data are presented as the means ± SD. *P < 0.05 represents statistical difference; ns represents no statistical difference.

Four bioinformatics databases, including JASPAR, HOCOMOCO, CISBP, and hTFtarget, were selected for NMNAT1 transcriptional factor screening, in which our data indicated that NMNAT1 was transcriptionally regulated by alcohol feeding (Fig. 1, D and J). Three genes, including *Irf1*, retinoic acid receptor gamma (*Rarg*), and microphthalmia transcription factor (*Mitf*), were predicted as potential nuclear factors of NMNAT1 (Fig. 2A). The expression of RARG was not affected by alcohol feeding (Fig. 2, B and C). Although MITF was up-regulated in the liver of AF mice (Fig. 2, B and C), Mitf knockdown did not affect the expression of NMNAT1 in cultured hepatocytes (fig. S6). Therefore, the involvement of either RARG or MITF was excluded. Further validation showed that nuclear IRF1 was significantly decreased by alcohol treatment at the protein level but not the mRNA level (Fig. 2, B and C). Similarly, nuclear IRF1 was also decreased in either the liver of patients with AH or primary hepatocytes isolated from the livers of chronic AF mice (Fig. 2, D and E). To test whether NMNAT1 was transcriptionally regulated by IRF1, the chromatin immunoprecipitation (ChIP) assay was performed by selecting the 2000–base pair (bp) upstream region in front of the NMNAT1 transcriptional start site as the prediction area of the IRF1 binding site. The ChIP quantitative polymerase chain reaction (ChIP qPCR) confirmed that IRF1 could bind with the NMNAT1 promoter region to regulate its gene expression directly (Fig. 2F). Activating IRF1 using its special agonist interferon-γ (IFN-γ) enhanced the combination of IRF1 to the promoter area of NMNAT1 (Fig. 2G), while the combination was decreased in the primary hepatocytes isolated from the livers of chronic AF mice (Fig. 2H). To consolidate this notion, hepatocyte-specific IRF1 knockdown mice were established via the caudal vein delivery of IRF1 short hairpin RNA constructed with adeno-associated viral serotype 8 (AAV8), by which a significant decrease in liver IRF1 expression and activity [reflected by mRNA expressions of the known downstream targets of IRF1, including nitric oxide synthase 2 (*Nos2*) and eukaryotic translation initiation factor 2 alpha kinase 2 (*Eif2ak2*) (*14*, *15*)] was attained (Fig. 2, I and J, and fig. S7). Consistent with our expectations, IRF1 loss decreased NMNAT1 expression and activity, along with decreased nuclear NAD^+^ content and NAD^+^/NADH ratio (Fig. 2, K to P). These data collectively suggested that IRF1 is a potential nuclear transcriptional factor of NMNAT1 and is involved in alcohol–down-regulated hepatic NMNAT1 in ALD.

### Oxidative stress promotes IRF1 ubiquitination degradation in hepatocytes

Furthermore, we explore the reason for the IRF1 decline. Oxidative stress plays a crucial role in the pathological progress of ALD (*16*). Here, we observed that hydrogen peroxide, a commonly used oxidative stress inducer, intervention led to a robust decrease in IRF1 and NMNAT1, which was inhibited by the ubiquitin-proteasome pathway antagonist MG132 but not by autophagy-lysosome degradation pathway inhibitors (bafilomycin A1 and chloroquine) pretreatment (fig. S8). In support of this phenomenon, we observed that hepatic IRF1 ubiquitination was enhanced in AF mice compared to that in PF mice (fig. S9). It has been reported that F-box and WD repeat domain containing 7 (FBXW7) functioned as an E3 ligase facilitating IRF1 ubiquitination degradation (*17*). Our data showed that FBXW7 was significantly up-regulated in AF mice (fig. S10). Genetically silencing FBXW7 attenuated IRF1 degradation and NMNAT1 down-regulation in response to hydrogen peroxide treatment in cultured hepatocytes (fig. S11). We identified that *N*-acetylcysteine (NAC), a potent antioxidant, abrogated the hydrogen peroxide–induced FBXW7 increase and IRF1 and NMNAT1 decrease in cultured hepatocytes (fig. S12). In addition, administrating mice with antioxidants, either NAC or mitoquinone (MitoQ), rescued alcohol-induced hepatic steatosis and liver injury and improved alcohol-led hepatic FBXW7 up-regulation and the loss of IRF1 and NMNAT1 expressions, along with the restoration of nuclear NAD^+^ levels and NAD^+^/NADH ratio (fig. S13). Those data collectively implied that FBXW7 was mechanistically involved in oxidative stress–stimulated IRF1 degradation and further NMNAT1 down-regulation in ALD.

### Liver-specific NMNAT1 knockout deteriorates alcohol-induced hepatic steatosis

**Fig. 3.**
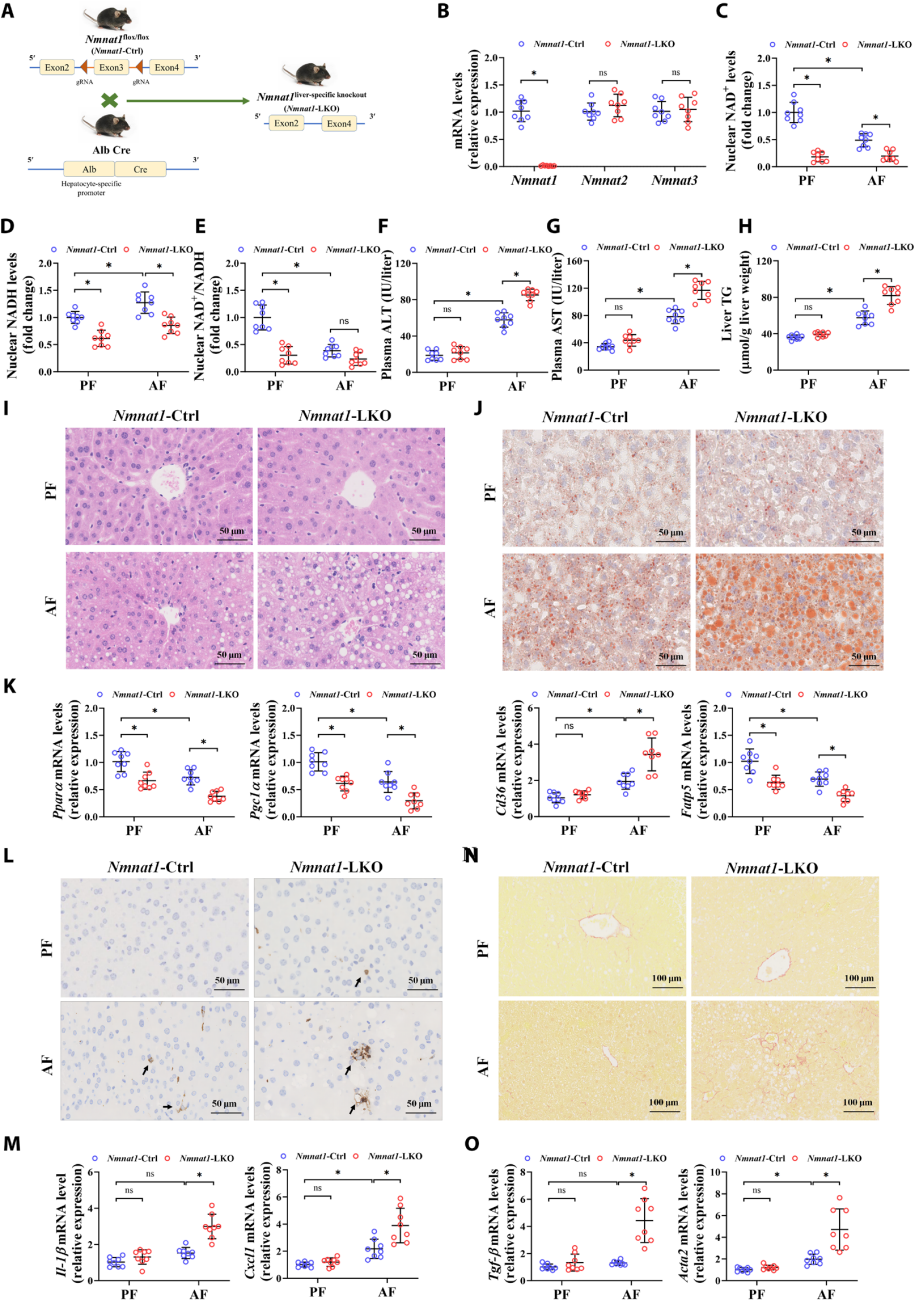
Liver-specific NMNAT1 knockout deteriorates alcohol-induced hepatic steatosis. (**A**) Schematic representation of the generation of hepatic *Nmnat1* knockout (*Nmnat1*-LKO) mice by crossing Nmnat1 Flox (*Nmnat1*-Ctrl) mice with Alb-Cre mice. (**B**) mRNA expressions of *Nmnat1*, *Nmnat2*, and *Nmnat3* in the livers of 12-week-old male mice fed a normal chow diet (*n* = 8). (**C** to **O**) *Nmnat1*-Ctrl and *Nmnat1*-LKO mice were given a PF or AF diet for 8 weeks plus a single binge of ethanol (5 g/kg) before 4 hours of tissue collection. (C) Liver nuclear NAD^+^ content (*n* = 8). (D) Liver nuclear NADH content (*n* = 8). (E) Liver nuclear NAD^+^/NADH ratio (*n* = 8). (F) Plasma ALT levels (*n* = 8). (G) Plasma AST levels (*n* = 8). (H) Liver TG content (*n* = 8). (I) Liver H&E staining. (J) Liver Oil red O staining (*n* = 4). (K) mRNA levels of lipid-metabolizing enzymes in the mouse liver (*n* = 8). (L) Liver immunohistochemistry of MPO staining. (M) mRNA expressions of *Il-1*β and *Cxcl1* in the mouse liver (*n* = 8). (N) Liver Sirius red staining. (O) mRNA expressions of *Tgf-*β and *Acta2* in the mouse liver (*n* = 8). Data are presented as the means ± SD. *P < 0.05 represents statistical difference.

To investigate the pathogenic role of hepatic NMNAT1 loss in ALD, liver-specific NMNAT1 knockout (*Nmnat1*-LKO) C57BL/6N mice were generated (Fig. 3, A and B, and fig. S14). A decline of NAD^+^ and NADH levels in both the nucleus and cytoplasm was observed in the *Nmnat1*-LKO mouse liver either with or without alcohol feeding (Fig. 3, C to E, and fig. S15). *Nmnat1*-LKO per se was not sufficient to induce, but it aggravated alcohol-induced hepatic steatosis and liver injury based on the measurements of ALT and AST levels in the plasma, liver TG level, hematoxylin and eosin (H&E) and Oil red O staining, liver weight, and liver weight–to–body weight ratio (Fig. 3, F to J, and figs. S16 and S17). *Nmnat1*-LKO also enhanced the alcohol-induced increase in plasma TG, total cholesterol (TC), and liver TC levels (fig. S16). A small array of mRNA expression assay on lipid metabolism–related genes showed that *Nmnat1*-LKO enhanced alcohol-dysregulated fatty acid β-oxidation and uptake (Fig. 3K and fig. S18). We also observed that *Nmnat1*-LKO significantly exacerbated alcohol-induced neutrophil infiltration and transcriptional up-regulation of pro-inflammatory cytokines, including interleukin-1β (*Il-1*β) and C-X-C motif chemokine ligand 1(*Cxcl1*) (Fig. 3, L and M, and fig. S19). In addition, an alcohol-induced early fibrosis–like change in the hepatic sinus area was aggravated in *Nmnat1*-LKO mice, accompanied by the mRNA up-regulation of transforming growth factor–1β (*Tgf*-β) and actin alpha 2 (*Acta2*) (Fig. 3, N and O). Lipolysis enhancement in adipose tissue is implicated in chronic alcohol consumption–induced hepatic steatosis (*18*). In line with a previous study, we observed that the fat weight and fat weight–to–body weight ratio were lowered by alcohol feeding along with the up-regulation of lipolytic enzymes, including adipose triglyceride lipase (*Atgl*) and hormone-sensitive triglyceride lipase (*Hsl*), and the increase in circulatory glycerol levels, which were not further aggravated in *Nmnat1*-LKO mice (fig. S20), preliminarily excluding the involvement of lipolysis in hepatic NMNAT1–regulated ALD. In addition, we observed that there are no statistical differences in both blood ethanol concentrations and the expressions of hepatic cytochrome P450 2E1, alcohol dehydrogenase 1, and acetaldehyde dehydrogenase 2 between AF *Nmnat1*-Ctrl and *Nmnat1*-LKO mice (fig. S21), preliminarily excluding the regulation of ethanol metabolism by hepatic NMNAT1.

### CSAD-regulated taurine metabolism is potentially involved in NMNAT1-regulated ALD

**Fig. 4.**
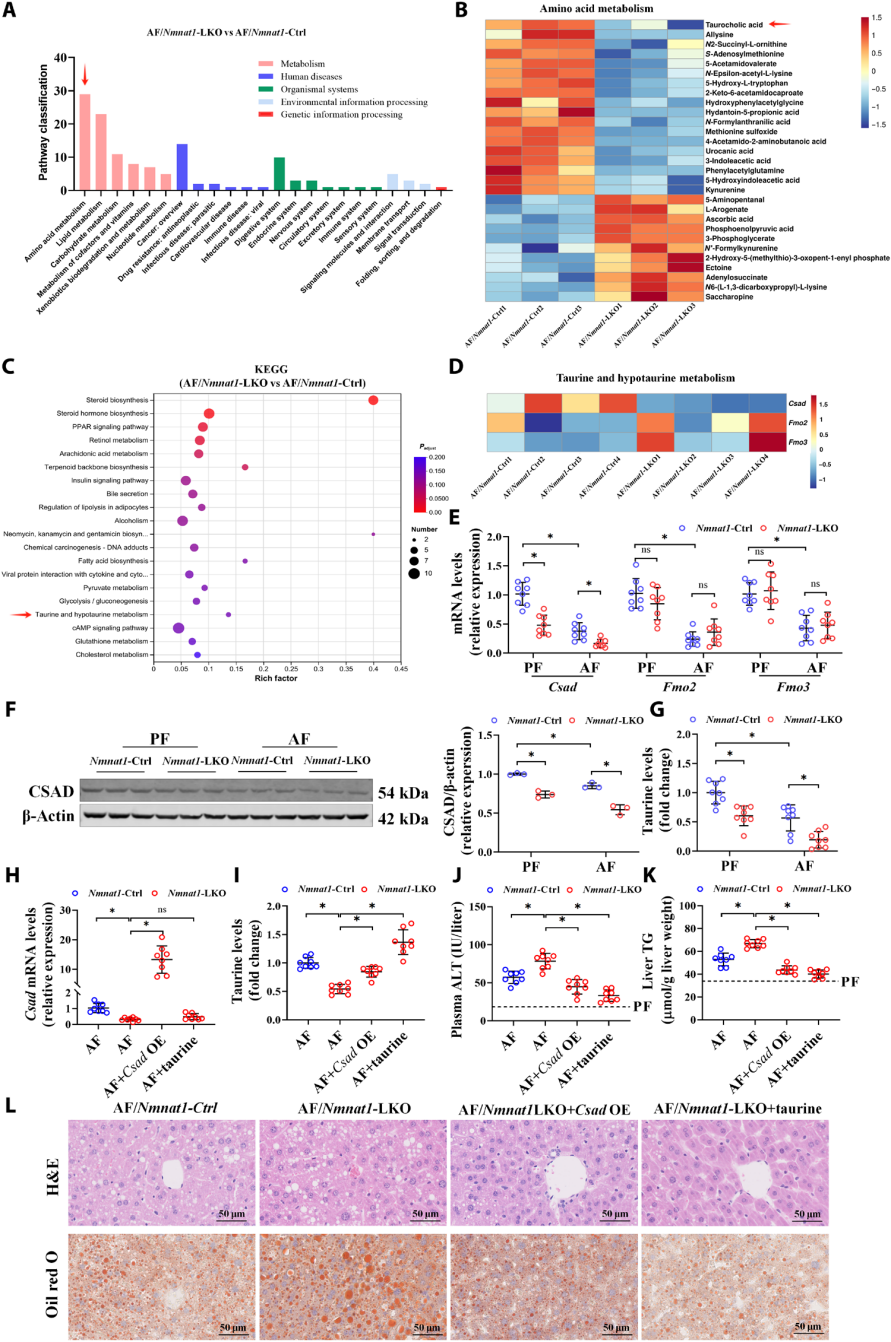
CSAD-regulated taurine metabolism is potentially involved in NMNAT1-regulated ALD. (**A** and **B**) Nontargeted metabolomic KEGG analysis of the differential metabolite enrichment pathway (A) and differential metabolites in amino acid metabolic pathways (B) in AF *Nmnat1*-Ctrl and *Nmnat1*-LKO mouse livers (*n* = 4). (**C** and **D**) Transcriptome KEGG enrichment signal pathway analysis (C) and differential genes expression in taurine and hypotaurine metabolism signaling pathways (D) in AF *Nmnat1*-Ctrl and *Nmnat1*-LKO mouse livers (*n* = 4). (**E**) mRNA expressions of *Csad*, *Fmo2*, and *Fmo3* in the mouse liver (*n* = 8). (**F**) CSAD protein expression in the mouse liver (*n* = 3). (**G**) Liver taurine content (*n* = 8). (**H** to **L**) Liver-specific CSAD overexpression mice were generated by caudal vein injection with an AAV8-constructed vector containing the *Csad* sequence. Mice injected with a null vector serve as the control. Taurine (1 g/kg per day) was supplemented in the diet of the AF + taurine group. (H) mRNA expressions of *Csad* in the mouse liver (*n* = 8). (I) Liver taurine content (*n* = 8). (J) Plasma ALT levels (*n* = 8). (K) Liver TG content (*n* = 8). (L) Liver H&E and Oil red O staining (*n* = 4). The protein band intensity was quantified by ImageJ. Data are presented as the means ± SD. *P < 0.05 represents statistical difference.

Multiomics were used in this study to explore the mechanism through which *Nmnat1*-LKO aggravated hepatic steatosis in ALD. Data from the metabolomic analysis of liver samples between *Nmnat1*-LKO and its littermate control with alcohol feeding showed statistical differences in multiple metabolic pathways, including amino acid and lipid metabolism (Fig. 4A). As shown in Fig. 4B, hepatic taurocholic acid and the other 18 metabolites were significantly decreased, while 11 metabolites in the amino acid metabolism pathway were increased in *Nmnat1*-LKO mice when compared to those in their littermate control. The RNA sequencing analysis identified 2548 statistical difference genes, among which 1085 were up-regulated and 1463 were down-regulated by *Nmnat1*-LKO compared to its littermate control in AF mouse liver (fig. S22). Kyoto Encyclopedia of Genes and Genomes (KEGG) analysis showed that the exacerbation of ALD by *Nmnat1*-LKO was closely associated with steroid biosynthesis, peroxisome proliferator–activated receptor (PPAR) signaling pathway, and taurine and hypotaurine metabolism (Fig. 4C). Considering the results from metabolomics and genomics, we speculated that the taurine metabolic pathway might be mechanistically involved in *Nmnat1*-LKO–deteriorated hepatic steatosis in ALD. Three genes with statistical differences in taurine and hypotaurine metabolism pathways, including *Csad*, flavin containing dimethylaniline monoxygenase 2 (*Fmo2*), and flavin containing dimethylaniline monoxygenase 3 (*Fmo3*), were identified by RNA sequencing analysis (Fig. 4D). Further verification revealed that hepatic CSAD was down-regulated by *Nmnat1*-LKO at both mRNA and protein levels (Fig. 4, E and F), along with a significant decrease in liver taurine content (Fig. 4G). Hepatocyte nuclear factor 4α (HNF4α) has been reported to be implicated in the regulation of CSAD (*19*). Analysis of publicly available human liver sample data showed that the transcriptional levels of both HNF4α and CSAD were decreased in the patients with AH (fig. S23). Besides, we observed that HNF4α was decreased by chronic alcohol feeding, and this abatement was further exacerbated by *Nmnat1*-LKO (fig. S24). In addition, alcohol-decreased hepatic HNF4α and CSAD expressions and taurine content in the liver were significantly improved by antioxidant intervention (fig. S25). Those data implied that HNF4α was potentially involved in regulating NMNAT1 on CSAD. To further confirm whether a hepatic CSAD decrease was implicated in *Nmnat1*-LKO–aggravated ALD, liver-specific CSAD overexpression (*Csad*-LOE) mice were generated via a caudal vein injection with a full-length *Csad* sequence–constructed plasmid with AAV8 (Fig. 4H and fig. S26). The data showed that *Csad*-LOE reversed the *Nmnat1*-LKO–induced decrease in taurine content in ALD mice (Fig. 4I). Meanwhile, *Nmnat1*-LKO–aggravated hepatic steatosis and liver injury in the presence of alcohol intervention were robustly rescued by *Csad*-LOE (Fig. 4, J to L). Taurine supplementation restored the hepatic taurine content and abrogated *Nmnat1*-LKO–aggravated pathological changes in the ALD mouse liver (Fig. 4, I to L). This evidence clearly indicated that CSAD-regulated taurine metabolism mechanistically contributes to the aggravation of ALD by *Nmnat1*-LKO.

### *Nmnat1*-LKO abrogates NMN supplementation–alleviated ALD

**Fig. 5.**
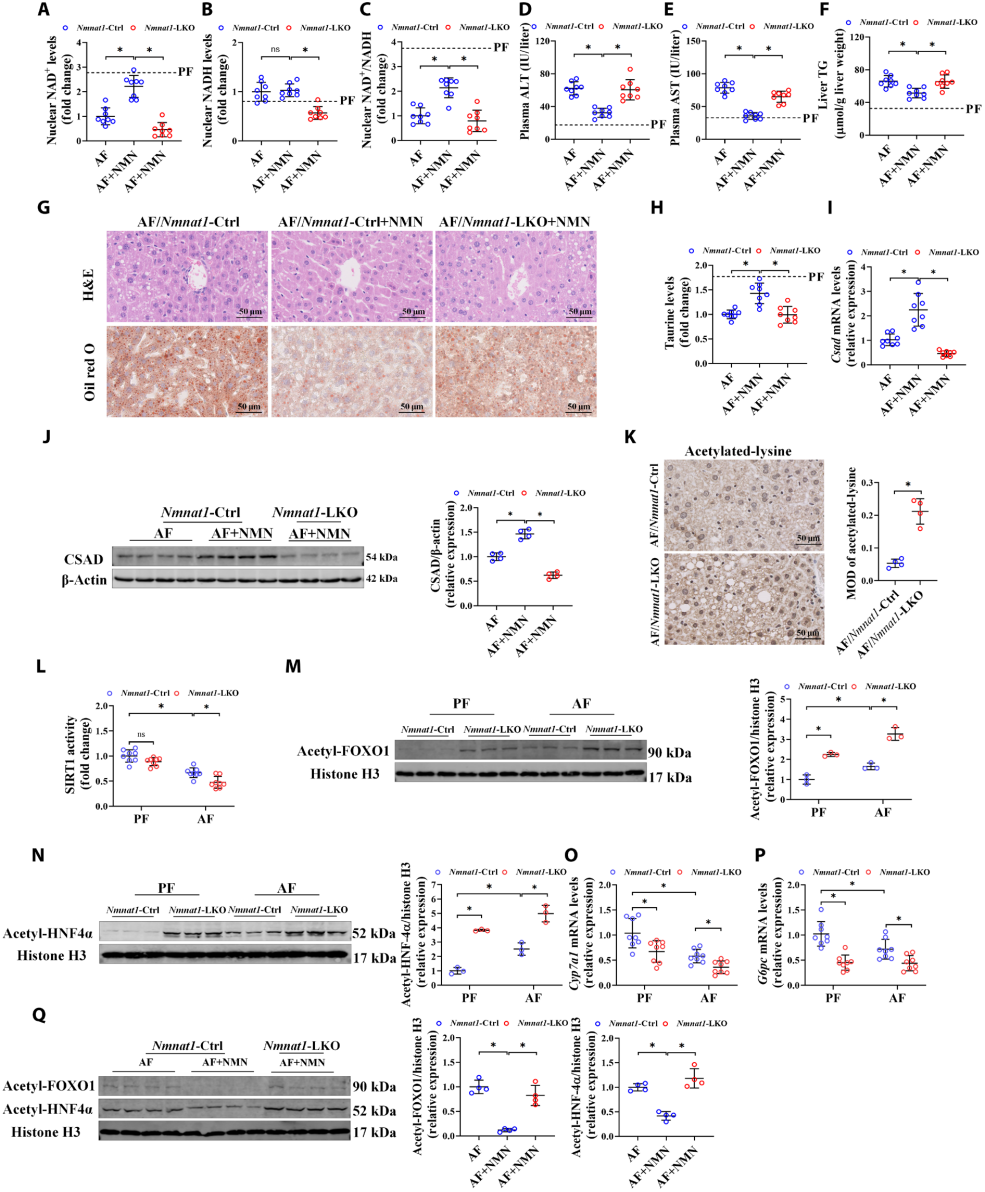
*Nmnat1*-LKO abrogates NMN supplementation–alleviated ALD. (**A** to **J**) *Nmnat1*-Ctrl and *Nmnat1*-LKO mice were supplemented with/without NMN (500 mg/kg per day) in the AF group. (A) Liver nuclear NAD^+^ content (*n* = 8). (B) Liver nuclear NADH content (*n* = 8). (C) Liver nuclear NAD^+^/NADH ratio (*n* = 8). (D) Plasma ALT levels (*n* = 8). (E) Plasma AST levels (*n* = 8). (F) Liver TG content (*n* = 8). (G) Liver H&E and Oil red O staining (*n* = 4). (H) Liver taurine content (*n* = 8). (I) mRNA expressions of *Csad* in the mouse liver (*n* = 8). (J) CSAD protein expression in the mouse liver (*n* = 4). (**K** to **P**) Liver immunohistochemistry of acetylated-lysine staining (K), SIRT1 activity (L), acetyl-FOXO1 protein expression (M), acetyl-HNF4α protein expression (N), and mRNA expressions of *Cyp7a1* (O) and *G6pc* (P) was detected in AF *Nmnat1*-Ctrl and *Nmnat1*-LKO mouse livers (*n* = 3 to 8). (**Q**) Acetyl-FOXO1 and acetyl-HNF4α protein expressions were measured in the liver of NMN-administered *Nmnat1*-LKO ALD mice (*n* = 4). The protein band intensity was quantified by ImageJ. Data are presented as the means ± SD. *P < 0.05 represents statistical difference. MOD, mean optical density.

Emerging evidence showed NMNAT1 exerting its biofunction via either a NAD^+^-dependent or NAD^+^-independent manner (*20*, *21*). To clarify whether the synthetic ability of nuclear NAD^+^ contributes to NMNAT1 loss–aggravated ALD, NMN, a direct precursor substrate of NMNAT1, was administered to AF mice. Our data showed that NMN intervention significantly restored the decline of nuclear NAD^+^ content and NAD^+^/NADH ratio caused by alcohol in the mouse liver (Fig. 5, A to C). Accordingly, chronic alcohol feeding–induced hepatic steatosis and liver injury were also alleviated by NMN treatment (Fig. 5, D to G). Meanwhile, *Nmnat1*-LKO significantly inhibited the protective role of NMN against ALD (Fig. 5, A to G). Besides, we also found that NMN supplementation improved the alcohol-induced decrease in hepatic taurine content and CSAD expression, and this beneficial role of NMN was inhibited by *Nmnat1*-LKO (Fig. 5, H to J). Those results together indicated that *Nmnat1*-LKO decreased CSAD and further aggravated ALD via a nuclear NAD^+^–dependent manner.

### *Nmnat1*-LKO deteriorates the alcohol consumption–induced SIRT1 activity decrease

In this study, we observed that chronic alcohol consumption promoted hepatic acetylation levels, especially in the nucleus area, which was obviously intensified by *Nmnat1*-LKO (Fig. 5K). Sirtuin 1 (SIRT1), a NAD^+^-dependent deacetylase located in the nucleus, has been reported to implicate alcohol-induced disruption of hepatic lipid metabolism (*22*). Here, we showed that *Nmnat1*-LKO significantly aggravated the alcohol-inhibited activity of SIRT1 and the alcohol-induced increase in acetylated forkhead box protein O1 (FOXO1) [a known downstream target reflecting SIRT1 activity (*23*)] without affecting SIRT1 protein expression (Fig. 5, L and M, and fig. S27). Notably, we observed that chronic alcohol consumption induced hepatic acetylation levels of HNF4α, and this induction was significantly amplified by *Nmnat1*-LKO (Fig. 5N). This attenuated the transcriptional activity of HNF4α (Fig. 5, O and P). Genetically silencing SIRT1 in cultured hepatocytes aggravated the hydrogen peroxide–induced enhancement of HNF4α acetylation and the decrease in CSAD expression and also led to a decrease in intracellular taurine levels (fig. S28). Meanwhile, antioxidant intervention significantly improved the hydrogen peroxide–induced hepatic SIRT1 expression and activity decrease, HNF4α acetylation increase, and CSAD expression decrease (fig. S29). This evidence suggested that a SIRT1-regulated HNF4α pathway might be involved in NMNAT1-regulated CSAD in ALD. We also identified that NMNAT1 regulated hepatic SIRT1 activity and HNF4α acetylation in a NAD^+^-dependent way, because NMN supplementation–ameliorated hepatic FOXO1 and HNF4α acetylation elevation and HNF4α transcriptional activity decrease were inhibited by *Nmnat1*-LKO in ALD mice (Fig. 5Q and fig. S30).

### PPARα activation abrogates *Nmnat1*-LKO–aggravated ALD

**Fig. 6.**
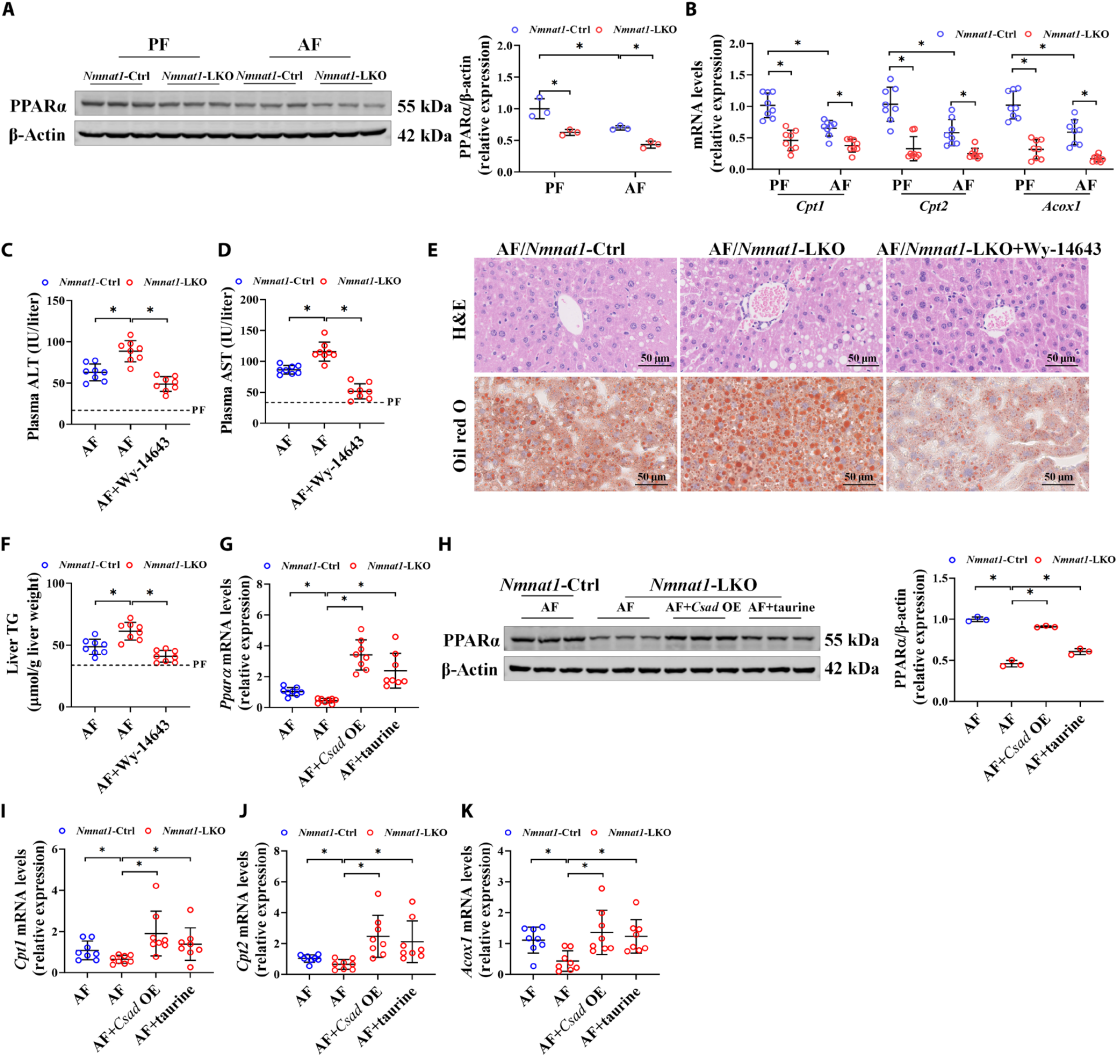
PPARα activation abrogates *Nmnat1*-LKO–aggravated ALD. (**A** and **B**) Expression of PPARα protein (A) and mRNA expressions of *Cpt1*, *Cpt2*, and *Acox1* (B) in AF *Nmnat1*-Ctrl and *Nmnat1*-LKO mouse livers (*n* = 3 to 8). (**C** to **F**) *Nmnat1*-Ctrl and *Nmnat1*-LKO mice were supplemented with/without Wy-14643 (10 mg/kg per day) in AF mice. (C) Plasma ALT levels (*n* = 8). (D) Plasma AST levels (*n* = 8). (E) Liver H&E and Oil red O staining (*n* = 4). (F) Liver TG content (*n* = 8). (**G** to **K**) Liver *Ppar*α mRNA (G), PPARα protein (H), and *Cpt1*, *Cpt2*, and *Acox1* mRNA [(I) to (K)] expressions were detected in the liver of AF liver–specific *Csad*-overexpressing or taurine-supplemented mice (*n* = 3 to 8). The protein band intensity was quantified by ImageJ. Data are presented as the means ± SD. *P < 0.05 represents statistical difference.

The implication of PPARα in *Nmnat1*-LKO–aggravated ALD was considered next on the basis of the analysis of RNA sequencing data (Fig. 4C). Our results showed that *Nmnat1*-LKO sensitized alcohol–down-regulated PPARα at both mRNA and protein levels (Figs. 3K and 6A). The hepatic activity of PPARα as a nuclear transcription factor was correspondingly decreased by *Nmnat1*-LKO, evidenced by the down-regulation of its identified downstream targets, including carnitine palmitoyltransferase 1 (*Cpt1*), carnitine palmitoyltransferase 2 (*Cpt2*), and acyl-CoA oxidase 1 (*Acox1*) (Fig. 6B). NMN supplementation significantly reversed alcohol-induced down-regulation of hepatic PPARα, which was inhibited by *Nmnat1*-LKO (fig. S31). Activating PPARα, using its special chemical agonist Wy-14643, was sufficient to reverse *Nmnat1*-LKO–aggravated hepatic steatosis and liver injury (Fig. 6, C to F, and fig. S32), indicating that PPARα inhibition was mechanistically involved in *Nmnat1*-LKO–aggravated ALD. Notably, we also observed that the *Nmnat1*-LKO–induced decrease in PPARα expression and activity in the AF mouse liver was strongly rescued by either *Csad*-LOE or taurine administration (Fig. 6, G to K), implying that PPARα was a potential down-target of CSAD/taurine–improved ALD.

### Liver-specific NMNAT1 overexpression ameliorates alcohol-induced hepatic steatosis

**Fig. 7.**
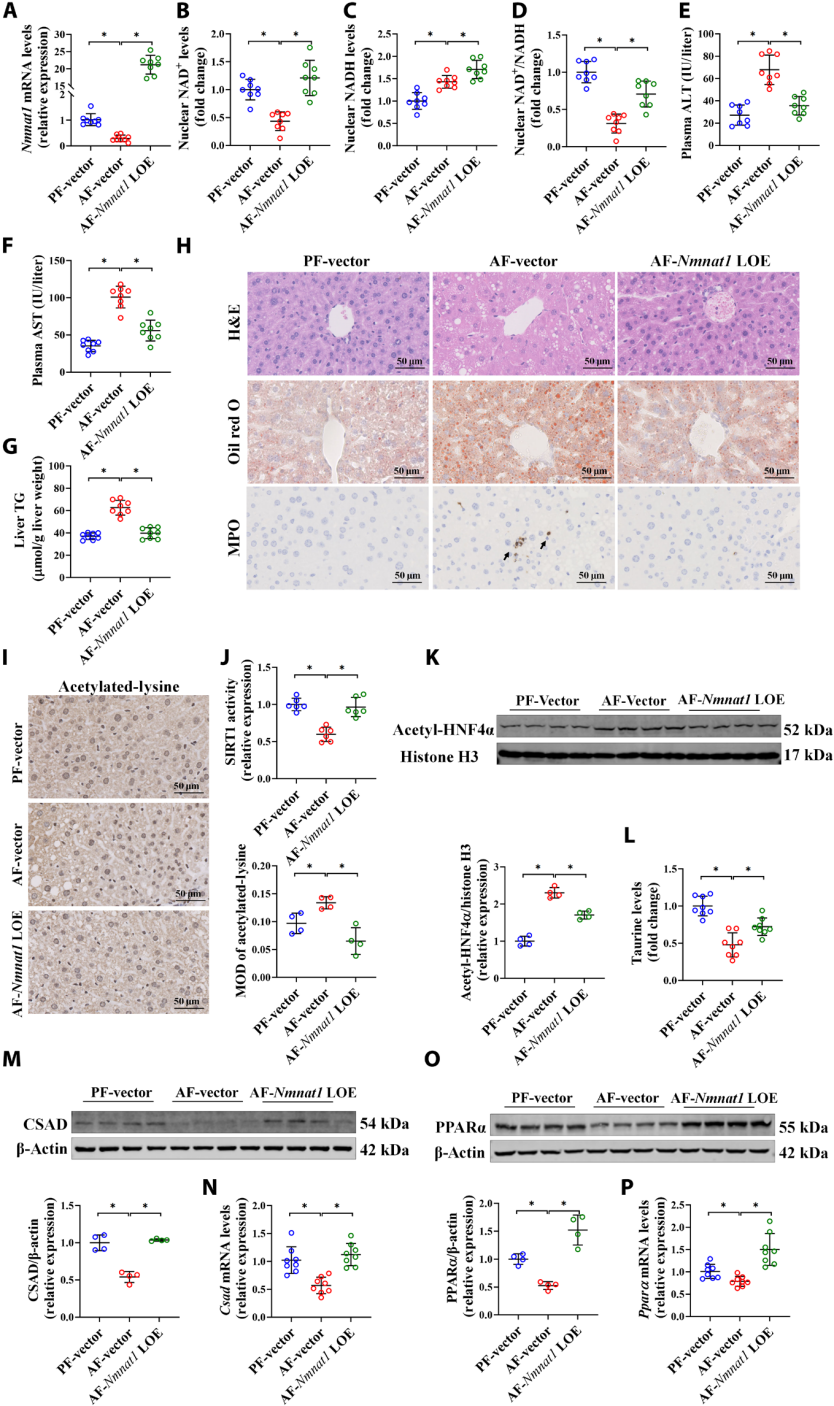
Liver-specific NMNAT1 overexpression ameliorates alcohol-induced hepatic steatosis. Liver-specific *Nmnat1* overexpression mice were generated by caudal vein injection with an AAV8-constructed vector containing the *Nmnat1* sequence. Mice injected with a null vector serve as the control. (**A**) mRNA expressions of *Nmnat1* in mice. (**B**) Liver nuclear NAD^+^ content (*n* = 8). (**C**) Liver nuclear NADH content (*n* = 8). (**D**) Liver nuclear NAD^+^/NADH ratio (*n* = 8). (**E**) Plasma ALT levels (*n* = 8). (**F**) Plasma AST levels (*n* = 8). (**G**) Liver TG content (*n* = 8). (**H**) Liver H&E, Oil red O, and MPO staining (*n* = 4). (**I**) Immunohistochemistry of acetylated-lysine staining (*n* = 4). (**J**) SIRT1 activity (*n* = 6). (**K**) Acetyl-HNF4α protein expression in the mouse liver (*n* = 4). (**L**) Liver taurine content (*n* = 8). (**M**) CSAD protein expression in the mouse liver (*n* = 4). (**N**) mRNA expressions of *Csad* in the mouse liver (*n* = 8). (**O**) PPARα protein expression in the mouse liver (*n* = 4). (**P**) mRNA expressions of *Ppar*α in the mouse liver (*n* = 8). The protein band intensity was quantified by ImageJ. Data are presented as the means ± SD. *P < 0.05 represents statistical difference.

To directly determine the therapeutic potential of NMNAT1 in ALD, liver-specific NMNAT1 overexpression (*Nmnat1*-LOE) mice were generated via caudal vein injection with a full-length NMNAT1 sequence–constructed plasmid with AAV8. Our data showed that *Nmnat1*-LOE significantly increased the mRNA expression of hepatic NMNAT1, along with enhanced nuclear NAD^+^ content and NAD^+^/NADH ratio (Fig. 7, A to D). *Nmnat1*-LOE markedly rescued alcohol-induced elevation of plasma ALT and AST and liver TG and restored adverse morphological changes in the mouse liver (Fig. 7, E to H). Accordingly, the chronic alcohol consumption–induced SIRT1 activity decrease, HNF4α acetylation increase, and the decrease in hepatic taurine content, CSAD expression, and PPARα expression in the mouse liver were reversed by *Nmnat1*-LOE (Fig. 7, I to P).

## DISCUSSION

**Fig. 8.**
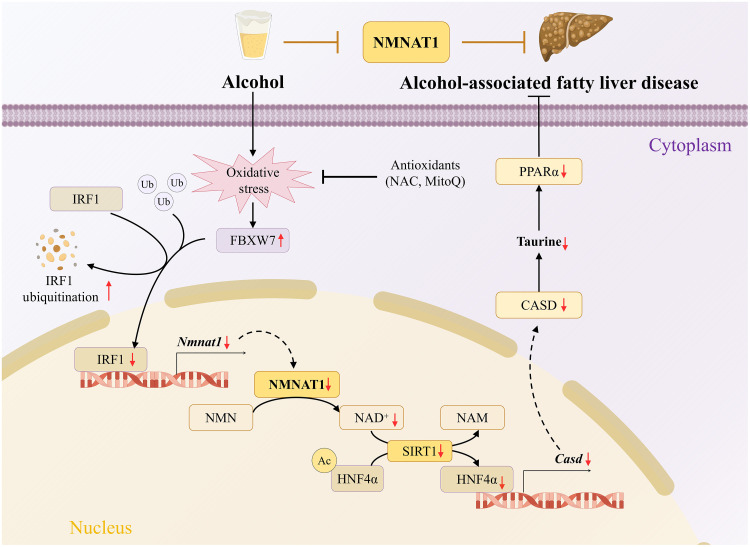
Schematic mechanistic illustration of hepatic NMNAT1–regulated ALD.

The main findings of this study include the following: (i) Chronic alcohol consumption is positively associated with hepatic NMNAT1 suppression, concomitant with a corresponding decrease in nuclear NAD^+^ content; (ii) IRF1 is a potential nuclear transcription factor of NMNAT1 and contributes to the alcohol-induced NMNAT1 decrease; (iii) *Nmnat1*-LKO aggravates hepatic steatosis and liver injury in ALD via a CSAD-associated pathway; (iv) *Nmnat1*-LOE alleviates ALD. A summarized schematic illustration of hepatic NMNAT1–regulated ALD is shown in Fig. 8. These data collectively implied that NMNAT1 is a promising therapeutic target for ALD treatment.

NAD^+^ decrease–disturbed glycolipid metabolism has been well recognized as the frontline mechanism of ALD (*7*, *24*), while restoring hepatic NAD^+^ levels through NAD^+^ precursor substance supplementation ameliorates ALD (*6*, *25*). The currently known pathways involved in alcohol-decreased NAD^+^ include the following: (i) Ethanol oxidation by either alcohol dehydrogenase or acetaldehyde dehydrogenase requires the consumption of NAD^+^ (*26*); (ii) alcohol decreased the expression of NAD^+^ synthase, e.g., nicotinamide phosphoribosyltransferase (*6*); (iii) alcohol increased the activity or expression of the enzyme using NAD^+^ as a substrate, e.g., poly(ADP-ribose) polymerase (*27*); (iv) alcohol increased the activity or expression of NAD^+^ precursor substance metabolic enzyme, e.g., nicotinamide *N*-methyltransferase (*24*). NMNAT1 is a critical enzyme in the nucleus that catalyzes NAD^+^ production. It has been reported that NMNAT1 was implicated in the pathological progress of retinal degeneration, axonal degeneration, tauopathy, and ischemic stroke (*21*, *28*–*31*). NMNAT1 loss impaired hepatic insulin signaling and mitochondrial function in high-fat diet–fed mice and aggravated doxorubicin-induced hepatotoxicity in mice (*20*, *32*). However, whether NMNAT1 participates in alcohol-decreased NAD^+^ and contributes to the further progression of ALD remains to be elucidated. To test this hypothesis, human liver samples, ALD animal models, and liver-specific gene–edited animals were used in this study. Our data showed that hepatic NMNAT1 was significantly decreased in patients with ALD, ALD mice, and alcohol-exposed primary hepatocytes. *Nmnat1*-LKO aggravated the alcohol-induced nuclear NAD^+^ decrease and sensitized hepatic steatosis and liver injury in ALD mice. Unlike the role of NMNAT1 in ALD, a recent study reported that *Nmnat1*-LKO was irrelevant with high-fat diet–induced hepatic steatosis (*33*); this might be due to the different roles of NAD^+^ between alcohol- and non–alcohol-induced fatty livers. Several studies have reported that high-fat diet treatment did not decrease or even enhance hepatic NAD^+^ levels in mice (*34*, *35*). NMNAT1 is essential for nuclear NAD^+^ biosynthesis in multiple tissues (*20*, *36*, *37*), while NMNAT1 also exerts its activity in a NAD^+^-independent manner (*21*). Here, we observed that NMN supply–alleviated ALD was inhibited by hepatic NMNAT1 depletion, implying that a NAD^+^-dependent manner was involved in the regulation of NMNAT1 on ALD. Replenishing hepatic NMNAT1 reversed lipid accumulation in ALD mice’s livers, implying that NMNAT1 is a promising therapeutic target for ALD management.

We observed that in AF *Nmnat1*-LKO mice, NMN intervention still partially restored nuclear NAD^+^ levels to those comparable to the AF *Nmnat1*-Ctrl group. This suggests that NMN can elevate nuclear NAD^+^ levels independently of NMNAT1. Although this study did not fully elucidate the mechanism underlying NMN-induced nuclear NAD^+^ elevation in *Nmnat1*-LKO mice, this phenomenon could partially be explained by the possibility of NAD^+^ transport between the cytoplasm and the nucleus (*38*, *39*), and cytoplasm-localized NMNAT2 also participated in catalyzing the conversion of NMN to NAD^+^. In addition, NMN-inhibited poly(ADP-ribose) polymerase-1 (*40*), which consumes NAD^+^ as a substrate, might also contribute to the observed increase in nuclear NAD^+^. In this study, we also tested the effect of chronic alcohol consumption on the other two isozymes of NMNAT and observed that alcohol feeding also down-regulated the hepatic expressions of NMNAT2 and NMNAT3 in ALD mice (fig. S33). Future studies are encouraged to address the role of NMNAT2 and NMNAT3 in ALD.

We further explored the regulating mechanism by which alcohol consumption leads to the decrease in NMNAT1. Here, we identified IRF1 as a nuclear transcriptional factor of NMNAT1 that contributes to the decrease in NMNAT1 caused by alcohol. IRF1, the first identified member of the IRF family, is originally identified as a nuclear factor that binds and activates the promoters of interferon genes (*41*). As a crucial component of the innate immune system, IRF1 regulates both innate and adaptive immunity in response to a variety of stimuli, including interferons and the pro-inflammatory cytokines, and protects the host against invading pathogens (*42*). Multiple biological functions of IRF1 have been recognized in antiviral defense, inflammatory response regulation, and the regulations of cell proliferation, differentiation, and apoptosis, as well as in the progression of diseases, including tumor, heart failure, ischemic stroke, and metabolic diseases (*43*–*45*). IRF1 is evolutionarily conserved in vertebrates and ubiquitously expressed in human cells, including hepatocytes. Emerging evidence showed the role of IRF1 in liver diseases, including hepatic ischemia-reperfusion injury, concanavalin A–induced liver injury, liver fibrosis, and hepatocellular carcinoma (*46*). Recent studies reported that the up-regulation of IRF1, stimulated by lipopolysaccharides with or without ethanol treatment in cultured primary bone marrow–derived macrophages, might be involved in ALD development (*47*, *48*). However, whether and how alcohol consumption regulates IRF1 in hepatocytes are still unclear. Here, we showed that IRF1 protein abundance but not the mRNA level was significantly decreased in the ALD mouse liver, implying that a protein degradation pathway was involved in this process. Our further data showed that inhibiting the ubiquitin-proteasome pathway but not the autophagy-lysosome degradation pathway abolished alcohol-induced IRF1 degradation. E3 ligase FBXW7 has been reported to facilitate IRF1 ubiquitination (*17*). Our results indicated that silencing FBXW7 reversed the alcohol-induced decrease in IRF1, implying that FBXW7-regulated IRF1 ubiquitination degradation contributed to alcohol-inhibited NMNAT1. It should be noted that the decrease in NMNAT1 in *Irf1* KD mice did not achieve the effect as alcohol intervention, and alcohol feeding–induced up-regulation of FBXW7 was not markedly high; therefore, we cannot rule out the possibility of other transcriptional regulatory factors or other E3 ligases being involved in alcohol-regulated NMNAT1 and IRF1, respectively. Although the exact mechanism underlining the up-regulation of FBXW7 in ALD was not fully illustrated, oxidative stress, a primary pathological mechanism of ALD (*16*, *49*), might be the initiator because we observed that antioxidant [NAC and MitoQ, which have been shown to alleviate hepatic oxidative stress in ALD mice (*5*, *50*)] treatment significantly rescued the dysregulation of FBXW7, IRF1, and NMNAT1 caused by alcohol. It is worth mentioning that we observed a complex feedback mechanism in the recovery of hepatic IRF1 in either NMN-administered or *Nmnat1*-LOE ALD mice (fig. S34); this may be related to the improvement of ALD.

Subsequently, the potential mechanism underlining NMNAT1-regulated ALD was investigated via multiomics analysis. We identified CSAD as a potential target in the regulation progress. CSAD is a rate-limiting enzyme in taurine anabolism, catalyzing cysteine sulfinic acid to produce hypotaurine; the latter generates taurine via oxidative dehydrogenation (*51*, *52*). Liver-specific overexpressing CSAD has been reported to alleviate hepatic steatosis in high-fat diet–fed mice (*53*), while the beneficial role of taurine in ALD improvement has been reported previously (*54*–*57*) and also been observed in our study (fig. S35). However, to the best of our knowledge, limited studies have been conducted to address how chronic alcohol consumption affects hepatic CSAD expression and taurine levels, as well as their implication in NMNAT1-regulated ALD. In this study, we found the following: (i) CSAD was significantly decreased in the AF mouse liver along with the decrease in taurine levels, and these decreases were aggravated in *Nmnat1*-LKO mice; (ii) either hepatic CSAD overexpression or taurine supply robustly reversed *Nmnat1*-LKO–aggravated lipid accumulation; (iii) *Nmnat1*-LOE improved the decrease in hepatic CSAD and taurine in ALD. This evidence collectively demonstrated that CSAD is a potential downstream target of NMNAT1 in regulating hepatic lipid metabolism in ALD. In addition, we have noticed that the decrease in neither NMNAT1 nor CSAD per se induced hepatic steatosis, while their loss of enhanced alcohol promoted the pathological process of the liver, implying that an environmental interaction manner jointly determined their roles in the occurrence of the disease.

How NMNAT1 regulates CSAD has been entirely unknown to date. Here, we showed that a nuclear NAD^+^–dependent manner was potentially associated with this progress, because the NMN supplementation–improved CSAD decrease induced by alcohol feeding was inhibited by *Nmnat1*-LKO. SIRT1 has been reported to implicate in NMNAT1-regulated multiple biological processes by controlling nuclear NAD^+^ biosynthesis (*31*, *58*–*60*). HNF4α has been identified as a direct nuclear transcription factor of CSAD that couples hepatic taurine production to bile acid synthesis in mice (*19*). It has been reported that alcohol decreased either SIRT1 or HNF4α, which was mechanistically involved in the pathological progress of ALD (*61*, *62*). Here, we observed that *Nmnat1*-LKO mice exhibited robustly hepatic SIRT1 activity and HNF4α expression suppression when compared to their littermate controls in response to alcohol feeding. Emerging evidence reported that hepatic HNF4α undergoes acetylation modification, which is negatively associated with its expression and transcriptional activity (*63*–*65*). The SIRT1 homolog sir2 could interact with HNF4α and promote its deacetylation and expression in *Drosophila* (*66*). Therefore, the level of acetylated-HNF4α was detected in this study, and our data showed that alcohol feeding significantly increased hepatic HNF4α acetylation, which was aggravated in *Nmnat1*-LKO ALD mice. We observed that *Nmnat1*-LOE rescued the alcohol consumption–caused decrease in SIRT1 activity and HNF4α expression. That evidence jointly implies that a SIRT1-regulated HNF4α pathway might be involved in NMNAT1-mediated CSAD in ALD. Because this study did not test the effect and degree of liver-specific activation of SIRT1 on the improvement of *Nmnat1*-LKO–aggravated ALD, we cannot rule out the possibility that targets other than SIRT1 may be involved in the regulation of ALD by NMNAT1, which requires further studies to address this issue.

In addition to the taurine metabolism pathway, our RNA sequencing data indicated that the PPARα pathway was also involved in NMNAT1-regulated ALD. The involvement of PPARα in the pathological progress of ALD has been well documented before (*67*). Here, we reported that *Nmnat1*-LKO aggravated while *Nmnat1*-LOE improved alcohol–down-regulated PPARα. Furthermore, the administration of the specific PPARα agonist Wy-14643 inhibited the ALD deterioration caused by *Nmnat1*-LKO, indicating that PPARα was also implicated in NMNAT1-regulated ALD. Given that PPARα is susceptible to acetylation, a modification regulated by SIRT1 (*68*), acetylated-PPARα was detected in SIRT1-silenced hepatocytes. Our data showed that silencing SIRT1 did not affect the level of acetylated-PPARα (fig. S36). Furthermore, neither SIRT1 expression nor PPARα acetylation was affected by Wy-14643 treatment (fig. S36). In addition, either CSAD-LOE or taurine supply rescued the *Nmnat1*-LKO–aggravated PPARα decrease. In support of this, several studies have reported that either CSAD-LOE or taurine administration up-regulated PPARα and alleviated hepatic steatosis in various animal models with fatty liver (*53*, *69*, *70*). This evidence implied that PPARα was potentially involved in NMNAT1/CSAD–regulated ALD.

There are several limitations in this study: (i) Only a single mouse strain (C57BL/6N) was used in this study, and experiments based on other strains of rodents and, ideally, nonhuman primates will enhance the clinical translational relevance. (ii) The mutations in NMNAT1 have been reported to be associated with human diseases (*71*–*73*). It is interesting to see the relationship between NMNAT1 variants and ALD via human cohort study. (iii) Lieber-DeCarli liquid alcohol diet, a commonly used commercialized feed to establish the ALD model, was used in this study. Unlike animal feed, dietary composition varied between individuals in humans. We previously reported that an isocaloric low-carbohydrate diet pattern improves ALD (*74*). It is worth exploring the interaction between the dietary pattern and NMNAT1 on ALD further. (iv) In this study, we observed FBXW7-regulated IRF1 degradation in ALD; however, the role of hepatic FBXW7 and IRF1 in ALD is still unclear and needs further investigations. (v) Only CSAD-mediated taurine and PPARα pathways were selected to search the downstream ways implicated in NMNAT1-regulated ALD. We cannot exclude the participation of other pathways because omics analysis has revealed multiple other pathways with statistical differences (Fig. 4C). This requires further work to elucidate the role of those pathways.

This study provides crucial evidence that chronic alcohol consumption leads to a hepatic NMNAT1 expression and activity decrease through an IRF1-involved transcriptional regulating mechanism, which in turn exacerbates alcohol-exhausted NAD^+^ and further promotes the pathological progression of ALD. CSAD-regulated taurine anabolism disorder contributes to NMNAT1 deprivation–aggravated ALD. Our findings suggest that NMNAT1 is a potential pharmacological target for ALD treatment.

## MATERIALS AND METHODS

### Human liver samples

Human liver samples from both healthy and AH individuals were provided by Z. Sun from Johns Hopkins University School of Medicine. The diagnosis of AH was made on the basis of the standard definitions and recommendations from the NIH/NIAAA AH Consortia. Liver tissue samples from explanted livers in patients with severe AH during liver transplantation or wedge biopsies from the donor livers (healthy) were either snap frozen in liquid nitrogen and stored at −80°C for biochemical assays. Tissue collection from explanted livers or biopsies from donor livers had been approved by Institutional Review Boards at Johns Hopkins Medical Institutions (IRB00107893 and IRB00021325), and written consent was obtained from all participants. The demographic and clinical characteristics of the patients have been described in previous studies (*75*, *76*).

### Animals

*Nmnat1^flox/flox^* mice under the C57BL/6J background were purchased from Cyagen Biosciences Inc. (S-CKO-13095, Guangzhou, China). In this mouse, the exon 3 of the *Nmnat1* gene is flanked by the LoxP sequence. *Nmnat1^flox/flox^* and albumin-cre (C001006, Guangzhou, China) mice were then backcrossed for four generations from C57BL/6J to generate the C57BL/6N background. Hepatocyte-specific *Nmnat1* KO (*Nmnat1*^LKO^) mice were generated by breeding *Nmnat1^flox/flox^* mice with albumin-cre mice. Specifically, we pair-bred *Nmnat1^flox/flox^*-Cre^+/−^ mice with *Nmnat1^flox/flox^*-Cre^−/−^ mice. The littermate offspring *Nmnat1^flox/flox^*-Cre^−/−^ (*Nmnat1*^Ctrl^) mice were used as control.

Liver-specific *Nmnat1* and *Csad* overexpression C57BL/6N mice were generated by lateral tail vein injection with recombinant AAV8 gene transfer vectors bearing a hepatocyte-specific albumin promoter combination with either a mouse *Nmnat1* full-length sequence (*Nmnat1* OE, HanBio Technology Co., Ltd., Shanghai, China) or *Csad* full-length sequence (*Csad* OE, HanBio Technology Co., Ltd., Shanghai, China). AAV8 vectors were administered by tail vein injection at a dose of 1 × 10^12^ viral titer/ml in a total volume of 100 μl per mouse 1 week before alcohol feeding.

The Lieber-DeCarli ALD model was established as described previously (*77*). Mice were fed with the Lieber-DeCarli alcohol liquid diet (AF) or isocaloric maltose dextrin control liquid diet (PF) for 9 weeks. The detailed protocols are shown in the Supplementary Materials.

Animal procedures were approved by the Institutional Animal Care and Use Committee of Zhejiang Chinese Medical University (approval number: 20210607-08). All mice were housed on a 12-h light-dark cycle at 23 ± 2°C with a 55 ± 5% relative humidity. Unless otherwise specified, all animals used in the study were male.

### Hepatocytes

Primary mouse hepatocytes were isolated as reported previously (*78*). Briefly, mice were anesthetized with pentobarbital (30 mg/kg body weight, intraperitoneally). Livers were first perfused with ice Hank’s balanced salt solution (Solarbio, Beijing, China) via the portal vein and then digested for 15 min at 37°C in digestion buffer [RPMI-1640 containing 1% fetal bovine serum (Biological Industries, ISR), deoxyribonuclease I (0.1 mg/ml; Sigma-Aldrich, St. Louis, MO), collagenase IV (0.2 mg/ml; Sigma-Aldrich, St. Louis, MO), and dispase II (0.8 mg/ml; Sigma-Aldrich, St. Louis, MO)]. After digestion, dissociated cells were collected and filtered through a 100-μm cell strainer (BD Biosciences, San Jose, CA) followed by centrifugation at 50*g* for 3 min at 4°C. Pellets were suspended with 20 ml of 40% ice-cold Percoll (GE Healthcare, Bensalem, PA) and centrifuged at 180*g* for 7 min at 4°C. After this step, primary hepatocytes were isolated.

*AML-12* cell lines were obtained from the American Type Culture Collection (Manassas, VA). *AML-12* cells were cultured in Dulbecco’s modified Eagle’s medium/F-12 containing 10% (v/v) fetal bovine serum, insulin (5 mg/ml; Solarbio, Beijing, China), transferrin (5 μg/ml; Solarbio, Beijing, China), selenium (5 ng/ml; Sigma-Aldrich, St. Louis, MO), and dexamethasone (40 ng/ml; Solarbio, Beijing, China).

### Biochemical analyses

Plasma was separated from blood after centrifugation at 3000 rpm for 15 min at 4°C and then kept at −80°C until use. Plasma levels of ALT and AST were determined by an ALT and AST determination kit (Nanjing Jiancheng Bio Co., Nanjing, China). TG, NAD^+^, and NADH in the tested samples were assayed using a TG assay kit or NAD^+^/NADH assay kit (Applygen, Beijing, China) according to the manufacturer’s recommended protocol, respectively. Liver taurine levels were measured by a commercially available competitive enzyme-linked immunoassay kit (ELK Biotechnology, Wuhan, China).

### Histological staining

For H&E staining, liver tissue samples were fixed in a 10% paraformaldehyde solution and further embedded in paraffin. Liver sections (4 μm) were deparaffinized in xylene and rehydrated through a series of decreasing ethanol concentrations. Sections were stained with H&E using a staining kit from G-Clone (Beijing, China). For the Oil red O stain, liver tissues were embedded in the Tissue-Tek optimal cutting temperature compound (Sakura, Tokyo, Japan). Frozen sections (8 μm thick) were subjected to Oil red O staining according to the instructions of the Oil red O stain kit (Solarbio, Beijing, China). For Sirius red staining, paraffin sections (4 μm) were hydrated and stained in Sirius red solution (G-Clone, Beijing, China) at room temperature for 1 hour. Images were captured by a Zeiss Axio Observer A1 inverted microscope (Oberkochen, Germany).

### Immunohistochemistry

Liver tissue paraffin sections were incubated with 3% hydrogen peroxide for 10 min to inactivate endogenous peroxidases and with normal serum (from the same species producing the secondary antibody) for 20 min. Then, the tissue sections were incubated with myeloperoxidase (MPO), acetylated-lysine, or NMNAT1 antibody at 4°C overnight, followed by incubation with the corresponding Dako EnVision^+^ System HRP Labelled Polymer Anti-Rabbit secondary antibody (Agilent, Santa Clara, CA) at room temperature for 30 min. Visualization was conducted with diaminobenzidine and hydrogen peroxide. Images were captured by a Zeiss Axio Observer A1 inverted microscope (Oberkochen, Germany).

### RNA interference

Cultured cells were transfected with FBXW7 small interfering RNA (siRNA) and SIRT1 siRNA (Santa Cruz Biotechnology, Santa Cruz, CA) using Lipofectamine 3000 according to the manufacturer’s instructions. In the control group, cells were transfected with NC siRNA (Santa Cruz Biotechnology, Santa Cruz, CA).

### Real-time qPCR

Total RNA was isolated from the tested tissues or cultured cells using TRIzol reagent (Invitrogen, Carlsbad, CA). Extracted RNA was then transcribed into cDNA by reverse transcription reagent kits (Monad, Wuhan, China) according to the manufacturer’s protocol. The primers were synthesized by Sangon Biotech Co., Ltd. (Shanghai, China), and primer sequences are shown in table S1. The abundance of mRNA was normalized to 18s ribosomal RNA.

### Western blot analysis

Western blots were performed as described previously (*77*). Briefly, liver tissues or cultured cells were lysed with radioimmunoprecipitation assay buffer (Boster Biological Technology, Wuhan, China) supplemented with protease and phosphatase inhibitors (Sigma-Aldrich, St. Louis, MO). The nuclear protein was obtained using a nuclear protein extraction kit (Beyotime, Shanghai, China). The nuclear extracts were used for the detection of nucleus-located proteins, including NMNAT1, IRF1, MITF, RARG, acetylated-FOXO1, and acetylated-HNF4α, in the samples from both mice and in vitro cells. The whole-tissue extracts were used for the detection of NMNAT1 and IRF1 in the samples from the human liver. The Western blotting for the other targets was performed on the whole-tissue/cell extracts. The protein samples were loaded into SDS–polyacrylamide gel electrophoresis gels and transferred to polyvinylidene difluoride membranes (Millipore, Bedford, MA). The membranes were blocked with 5% fat-free milk in tris-buffered saline with Tween 20 (Servicebio, Wuhan, China) and reacted with primary antibodies at 4°C for 12 to 16 hours. After washing with tris-buffered saline with Tween 20, the staining was incubated with IRDye anti-rabbit or anti-mouse secondary antibodies (1:20,000; LI-COR) for 1 hour and then visualized using an Odyssey Infrared Imager (LI-COR). The immunoblots were quantified by measuring the density of each band with ImageJ software. The antibodies are listed in table S2.

### Metabolomics analysis

Liver tissues were extracted with methanol:water (4:1, v/v) containing L-2-chlorophenylalanine (0.02 mg/ml), followed by vortexing, centrifugation, and supernatant collection for liquid chromatography–tandem mass spectrometry analysis. The liquid chromatography–mass spectrometry analysis was conducted using the UHPLC-Q Exactive system from Thermo Fisher Scientific. After mass spectrometry detection, the raw data of liquid chromatography–mass spectrometry were preprocessed by Progenesis QI (Waters Corporation, Milford, US) software, and a three-dimensional data matrix in CSV format was generated. After the database search, the preprocessed data were uploaded to the Majorbio cloud platform (https://cloud.majorbio.com) for data analysis. The R package ropls (version 1.6.2) performed principal components analysis and orthogonal least partial squares discriminant analysis. In addition, Student’s *t* test and the fold difference analysis were performed. The selection of significantly different metabolites was determined on the basis of the VIP (variable importance in the projection) obtained by the orthogonal least partial squares discriminant analysis model and the *P* value of Student’s *t* test, and metabolites with VIP > 1 and *P* < 0.05 were regarded as significant differential metabolites. Differential metabolites among the two groups were summarized and mapped into their biochemical pathways through metabolic enrichment and pathway analysis based on a database search (KEGG; www.genome.jp/kegg/).

### RNA sequencing and analysis

Total RNA was isolated using TRIzol, and RNA quality was assessed using a 2100 Expert Bioanalyzer (Agilent, Palo Alto, CA) and sent for commercial library preparation and sequencing by Majorbio Biotech (Shanghai, China) on an Illumina HiSeq 2000. Short sequence reads were analyzed on the Majorbio I-Sanger Cloud Platform (https://cloud.majorbio.com/). RNA sequencing data were generated and analyzed as described (*74*). Statistical gene set analysis was performed using the nonparametric Kruskal-Wallis test to determine differential expression at the gene level (*P* < 0.05, fold change of ±2). Partek flow default settings were used in all analyses. The data have been deposited in the Gene Expression Omnibus (GEO) data repository (NCBI accession number: PRJNA1159526).

### ChIP analysis

ChIP analysis was performed according to the instructions for the Pierce Agarose ChIP Kit (Thermo Fisher Scientific, US). AML-12 cells were treated with either DMSO (dimethyl sulfoxide) or IFN-γ for 6 hours. Cells were fixed by adding 16% formaldehyde directly into the culture media, which was diluted to 1% lastly. After 10-min fixing at room temperature, the media were added to glycine to stop fixing. The upstream 2000-bp region of the NMNAT1 transcription start site was selected as the gene promoter region. The IRF1 binding site was predicted in the JASPAR database (http://jaspar.genereg.net/). The purified, immunoprecipitated DNA was analyzed by qPCR. The primers used in ChIP qPCR were as follows: forward: 5′GGAGCCTGACCTCTCTTCGT-3′; reverse: 5′TACAGTGTACTCATGTGGGCT-3′.

### Enzyme activity

The commercialized enzyme activity detection kit was used for the measurement of NMNAT1 (Abcam, no. ab221820) and SIRT1 (Abcam, no. ab156065) activity according to the manufacturer’s protocol.

### Statistical analysis

The statistical analysis was performed with GraphPad Prism (GraphPad Software 8.0.1). Data are presented as the means ± SD. All the results were compared using a two-sided independent *t* test to compare two groups, and a one-way analysis of variance (ANOVA) followed by Tukey’s post hoc test was applied to adjust for multiple comparisons. For all tests, *P* < 0.05 was considered significant. All data points here represent individual biological replicates.

## Supplementary Materials

### The PDF file includes:

Supplementary Materials and Methods Figs. S1 to S36 Tables S1 and S2 Legend for data S1 References
