# Supplementary material for: Hepatic NMNAT1 is required to defend against alcohol-associated fatty liver disease
Source: Sci Adv. 2025 Jun 27;11(26):eadt6195. doi: 10.1126/sciadv.adt6195 (PMC12204165; doi:10.1126/sciadv.adt6195)
Supplement: Supplementary file 1 — Supplementary Materials and Methods Figs. S1 to S36 Tables S1 and S2 Legend for data S1 References [file sciadv.adt6195_sm.pdf]

Science Advances
AAAS

Supplementary Materials for

Hepatic NMNAT1 is required to defend against alcohol-associated fatty liver disease

Qinchao Ding *et al.*

Corresponding author: Songtao Li, lisongtao@zcmu.edu.cn

Sci. Adv. **11**, eadt6195 (2025)
DOI: 10.1126/sciadv.adt6195

The PDF file includes:

Supplementary Materials and Methods
Figs. S1 to S36
Tables S1 and S2
Legend for data S1
References

Other Supplementary Material for this manuscript includes the following:

Data S1

Supplementary Materials and Methods

Animals study

Animal procedures were approved by the Institutional Animal Care and Use Committee of Zhejiang Chinese Medical University (Approval number: 20220919-27). All Mice were housed on a 12-h light-dark cycle at 23 ± 2°C with 55 ± 5% relative humidity. Lieber-DeCarli ALD model was established as described previously (*77*). Mice were fed with the Lieber-DeCarli alcohol liquid diet (alcohol-fed; AF) or isocaloric maltose dextrin control liquid diet (pair-fed; PF) for nine weeks. In the first week, mice were given a modified Lieber DeCarli liquid diet (Trophic Animal Feed High-Tech Co., Ltd. Nantong, China) without alcohol to adapt to the liquid diet. Subsequently, the ethanol concentrations in the alcohol diet ranged from 3.5% to 4.06%, with an incremental increase of 0.14% every two weeks. The amount of food given to the PF mice was the same as the AF mice consumed the previous day. On the day of sacrifice, the mice in the AF group received an ethanol gavage at a dose of 4 g/kg body weight. Five hours later, the mice were euthanized under anesthesia using an intraperitoneal injection of pentobarbital solution (40 mg/kg body weight). Plasma and tissue samples were then collected for further analysis. Taurine was administrated by gavage at a dose of 1 g/kg body weight/day. For developing a mouse model for alcohol-associated liver fibrosis, mice were injected intraperitoneally with 20% carbon tetrachloride (CCl_4) diluted in corn oil (2 μL/g body weight) or vehicle (corn oil) once every three days, during alcohol liquid feed feeding. N-acetylcysteine (NAC) was administrated by gavage at a dose of 40 mg/kg body weight/day. Mitoquinone (MitoQ) was intervened by intraperitoneal injection at a dose of 5 mg/ kg body weight/day. Unless otherwise specified, animal experimental data are sourced from male animals.

Biochemical analyses

Plasma levels of free fatty acid (FFA) and glycerol were determined by glycerol assay kit (Nanjing Jiancheng Bio Co., Nanjing, China). Plasma and liver total cholesterol (TC) were determined using a TC assay kit (Applygen, Beijing, China) according to the manufacturer's recommended protocol.

Hepatocytes

VL-17A, a HepG2-based reformed cell line, was conducted according to previous study (*79*). The HepG2 cell line was obtained from the Cell Bank of the Chinese Academy of Sciences (Shanghai, China). HepG2 cells were stably transfected with both cytochrome P450 2E1 (CYP2E1) and alcohol dehydrogenase (ADH) to construct the *VL-17A* cell line. *VL-17A* cells were cultured in DMEM (Gibco, Waltham, MA) supplemented with 10% FBS and 100 U/mL penicillin/streptomycin at 37°C in a humidified atmosphere of 5% CO_2 and 95% air.

Ethanol measurement

Plasma ethanol levels were determined by gas chromatography with mass spectrometry (GC-MS). The GC/MS method was modified from work by Sun et al (*80*). All procedures were conducted at 0~4 °C. In brief, 100 μL of plasma was mixed with 500 μL of distilled water containing 50 μg/mL tert-butanol (internal standard for ethanol). The samples were centrifuged at 2000 g × 15 min at 4 °C after vortexing for 30 s. The supernatant of each sample was quantitatively transferred into a 20-mL headspace vial and capped immediately. Headspace vials

were then loaded onto the tray of a headspace sampler coupled to a GC-MS instrument (Agilent Technologies, Santa Clara, CA). The concentrations of ethanol in plasma were calculated by comparing the integrated areas of ethanol peaks on the gas chromatograms with those of internal standards added in each sample.

RNA interference

Cultured cells were transfected with special siRNA for MITF (Santa Cruz Biotechnology, Santa Cruz, CA) using Lipofectamine 3000 according to the manufacturer's instructions. In the control group, cells were transfected with NC siRNA (Santa Cruz Biotechnology, Santa Cruz, CA).

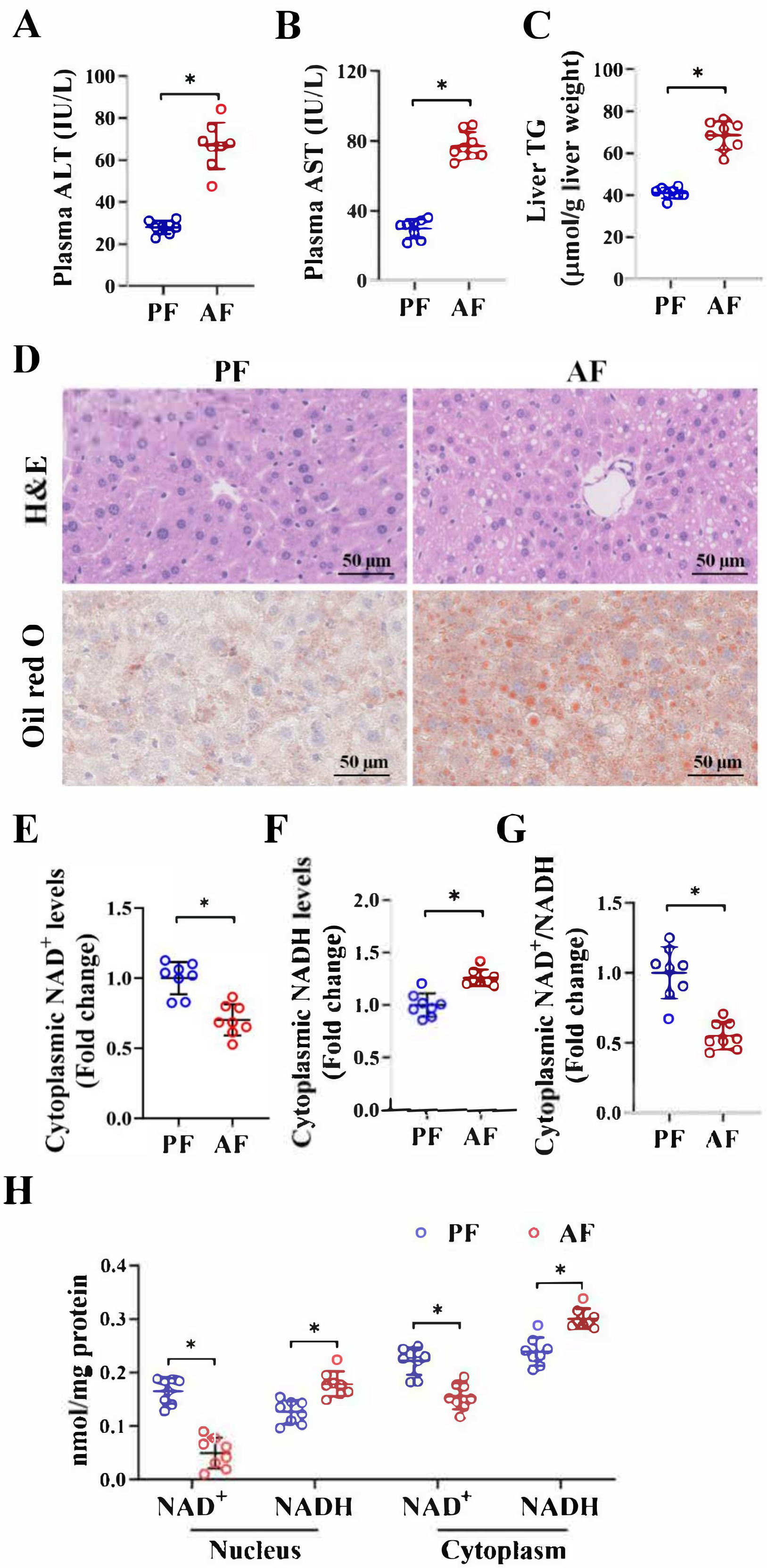

A
Plasma ALT (IU/L)
PF
AF
B
Plasma AST (IU/L)
C
Liver TG (μmol/g liver weight)
D
H&E
Oil red O
50 μm
E
Cytoplasmic NAD+ levels (Fold change)
F
Cytoplasmic NADH levels (Fold change)
G
Cytoplasmic NAD+/NADH (Fold change)
H
nmol/mg protein
NAD+
NADH
Nucleus
Cytoplasm

Figure S1. Chronic alcohol consumption induced hepatic steatosis and liver injury, as well as hepatic nuclear and cytoplasmic NAD^+ decrease. (A) Plasma ALT levels (n = 8). (B) Plasma AST levels (n = 8). (C) Liver total triglyceride (TG) content (n = 8). (D) Liver H&E and Oil red O staining (n = 4). (E) Cytoplasmic NAD^+ content (n = 8). (F) Cytoplasmic NADH content (n = 8). (G) Cytoplasmic NAD^+/NADH ratio (n = 8). (H) NAD^+ and NADH concentrations in the nucleus and cytoplasm (n = 8). Data are presented as means ± SD. *$P < 0.05$ represents statistical difference. PF, pair-fed; AF, alcohol-fed.

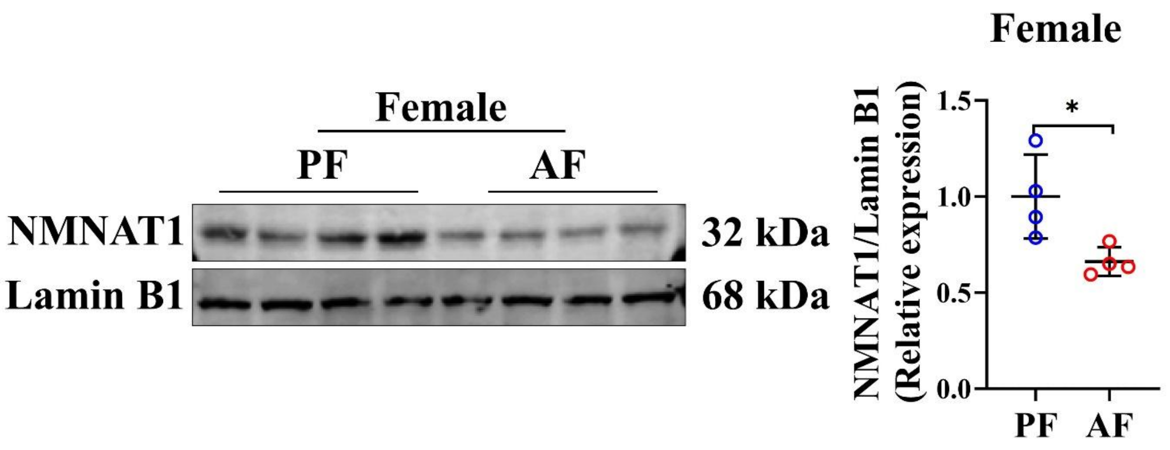

Figure S2. Chronic alcohol consumption induced a decrease of hepatic NMNAT1 in female mice. The nuclear protein was extracted from the liver tissues of female mice. The expression of nuclear NMNAT1 was detected by Western-blot (n = 4). Protein band intensity was quantified by ImageJ. Data are presented as means ± SD. *$P < 0.05$ represents statistical difference. PF, pair-fed; AF, alcohol-fed.

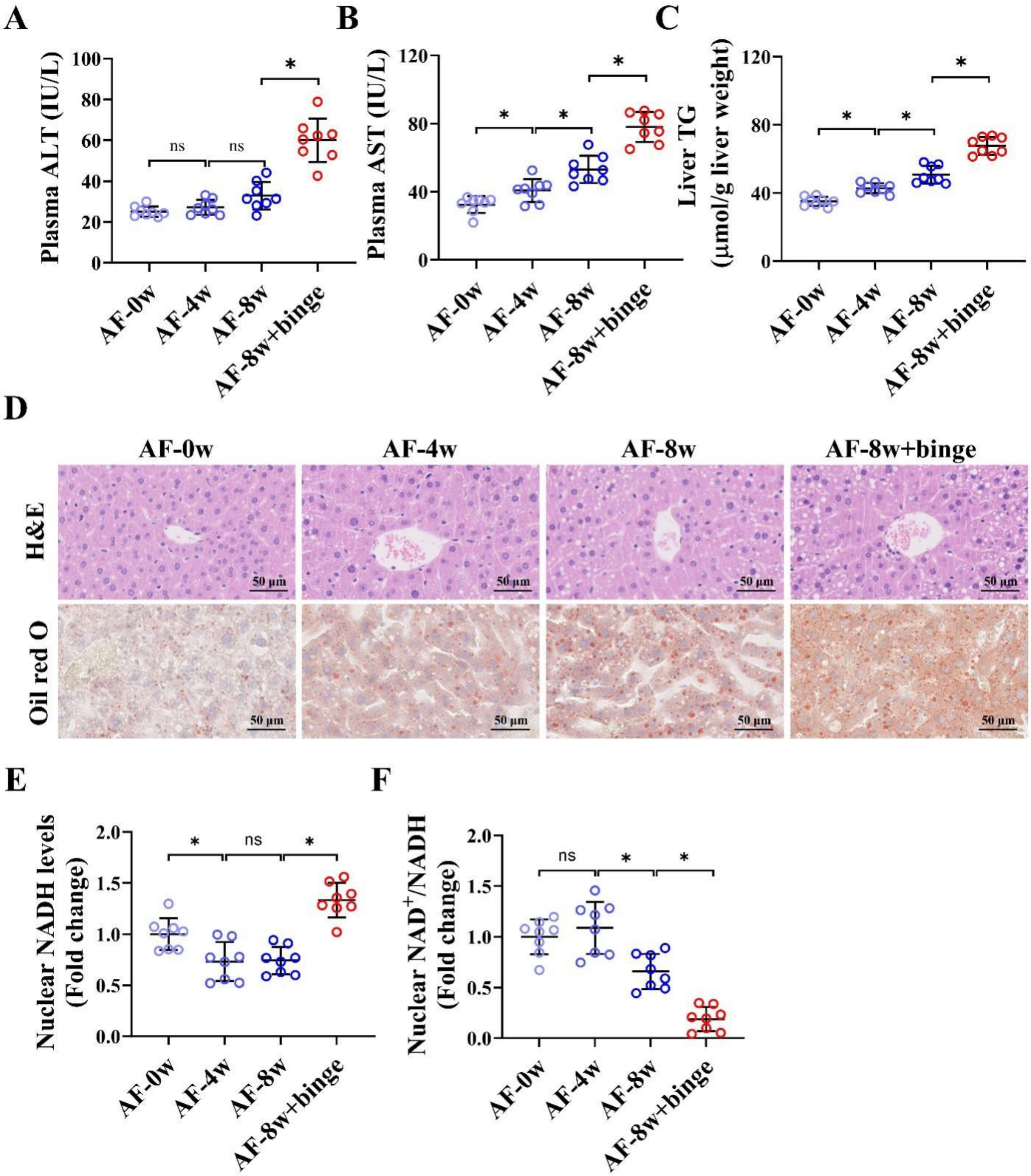

Figure S3. Chronic alcohol consumption induced dynamic alterations in hepatic steatosis and liver injury, as well as nuclear NADH and NAD$^+$/NADH ratio. (A) Plasma ALT levels (n = 8). (B) Plasma AST levels (n = 8). (C) Liver total triglyceride (TG) content (n = 8). (D) Liver H&E and Oil red O staining (n = 4). (E) Nuclear NADH content (n = 8). (F) Nuclear

NAD^+/NADH ratio (n = 8). Data are presented as means ± SD. $*P < 0.05$ represents statistical difference. AF, alcohol-fed; ns, represents no statistical difference.

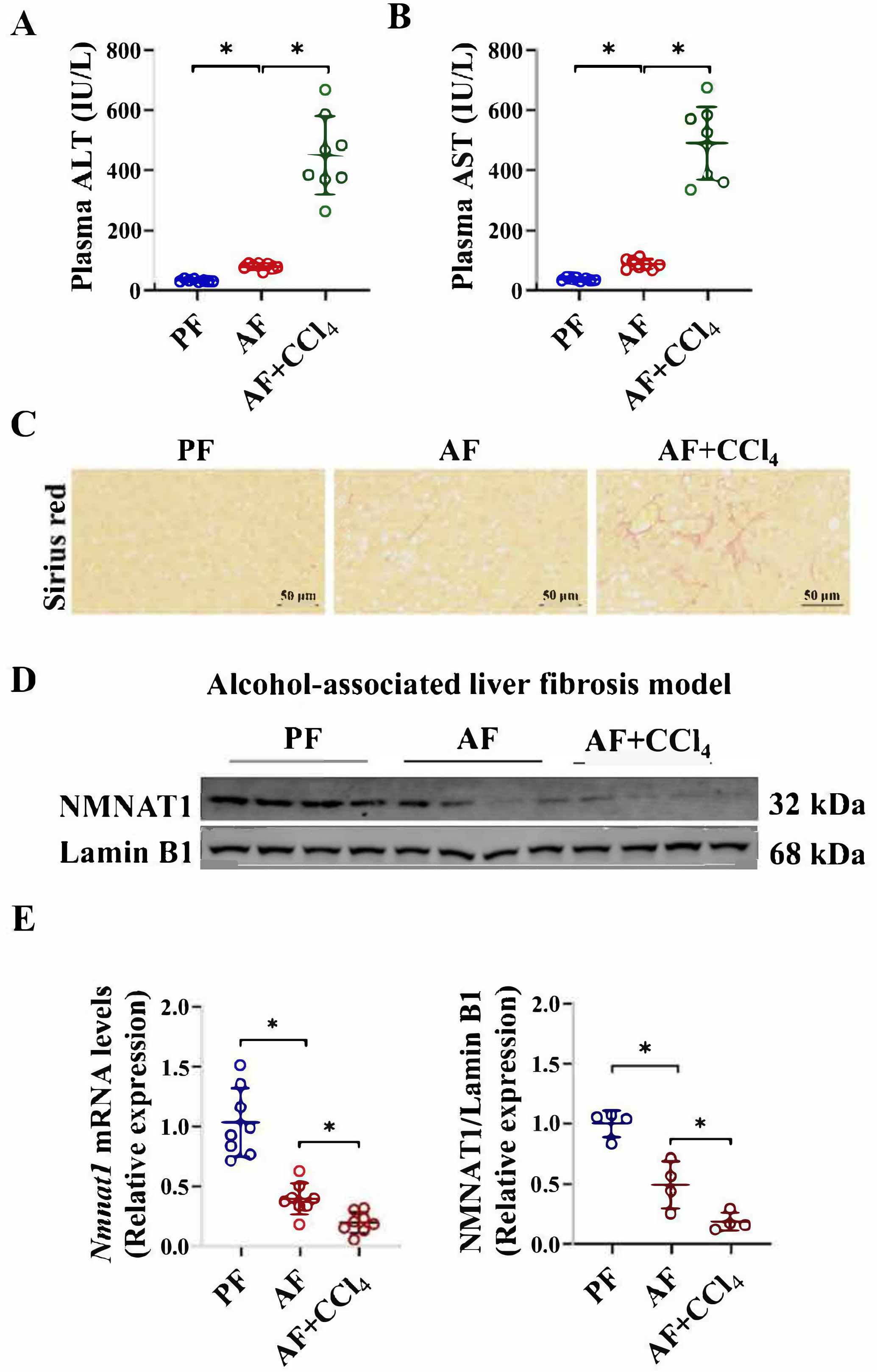
A
B
C
D
E
Plasma ALT (IU/L)
Plasma AST (IU/L)
800
600
400
200
0
PF
AF
AF+CCl4
Sirius red
50 µm
Alcohol-associated liver fibrosis model
NMNAT1
Lamin B1
32 kDa
68 kDa
Nmnat1 mRNA levels (Relative expression)
NMNAT1/Lamin B1 (Relative expression)
2.0
1.5
1.0
0.5
0.0
*

Fig S4. Lieber-DeCarli liquid alcohol diet combining with CCl_4 treatment established alcohol-related liver fibrosis model in mice. (A) Plasma ALT levels (n = 8). (B) Plasma AST levels (n = 8). (C) Sirius red staining (n = 4). (D) Nuclear NMNAT1 protein levels in the liver (n = 4). (E) Liver *Nmnat1* mRNA expression levels (n = 8). Protein band intensity was quantified by ImageJ. Data are presented as means ± SD. *$P < 0.05$ represents statistical difference. PF, pair-fed; AF, alcohol-fed.

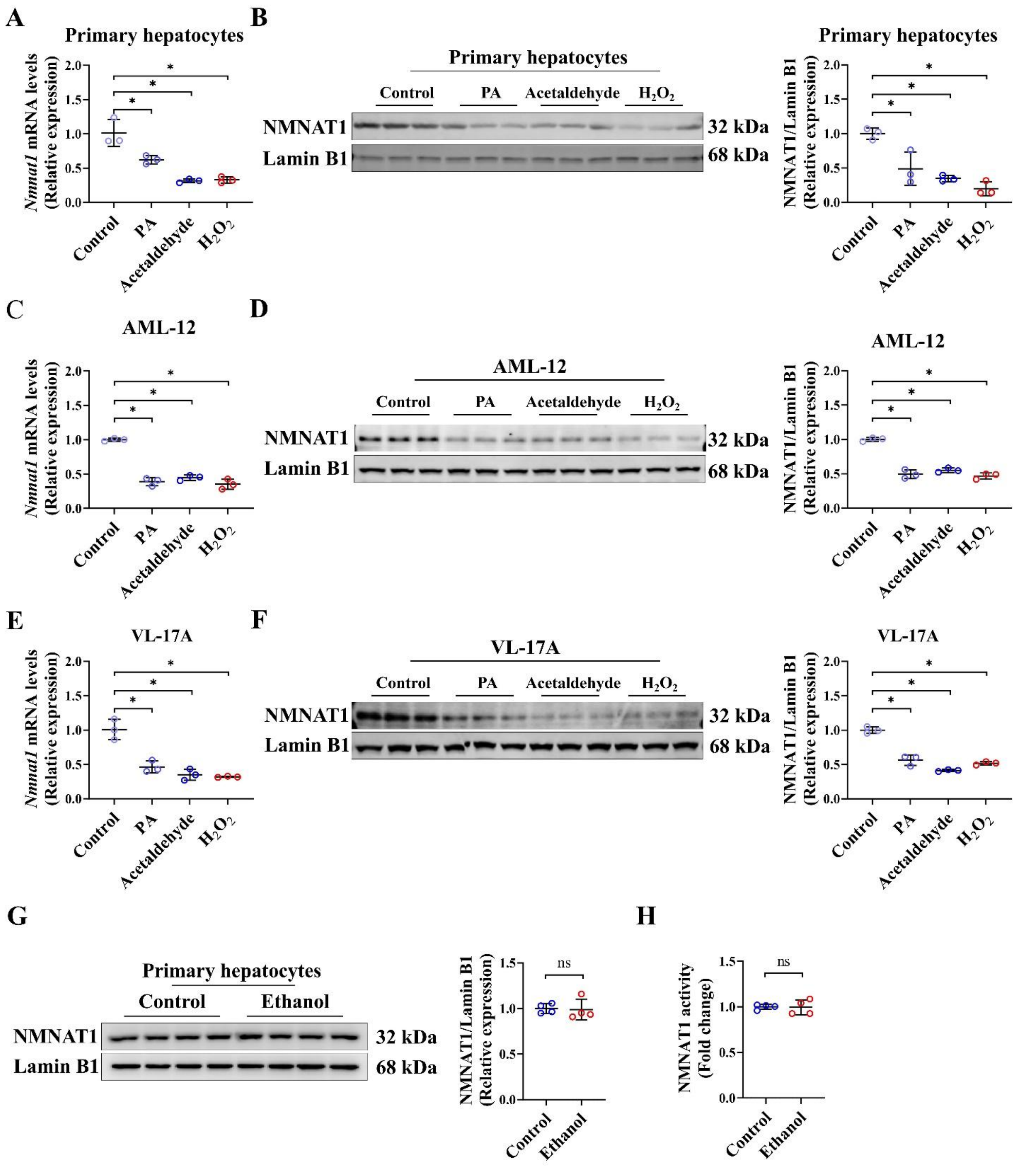

Fig S5. NMNAT1 expression was down-regulated by lipotoxicity, acetaldehyde, and hydrogen peroxide (H_2O_2) in hepatocytes. Primary mouse hepatocytes, AML-12, and VL-17A were treated with PA (0.5 mM), acetaldehyde (0.1 mM), H_2O_2 (0.1 mM), and ethanol (100 mM) for 24 h, respectively. (A-G) The mRNA and nuclear protein expressions of NMNAT1 were detected as indicated in each panel (n = 3-4). (H) NMNAT1 activity was measured (n = 4).

Protein band intensity was quantified by ImageJ. Data are presented as means ± SD. *$P < 0.05$ represents statistical difference; ns, represents no statistical difference.

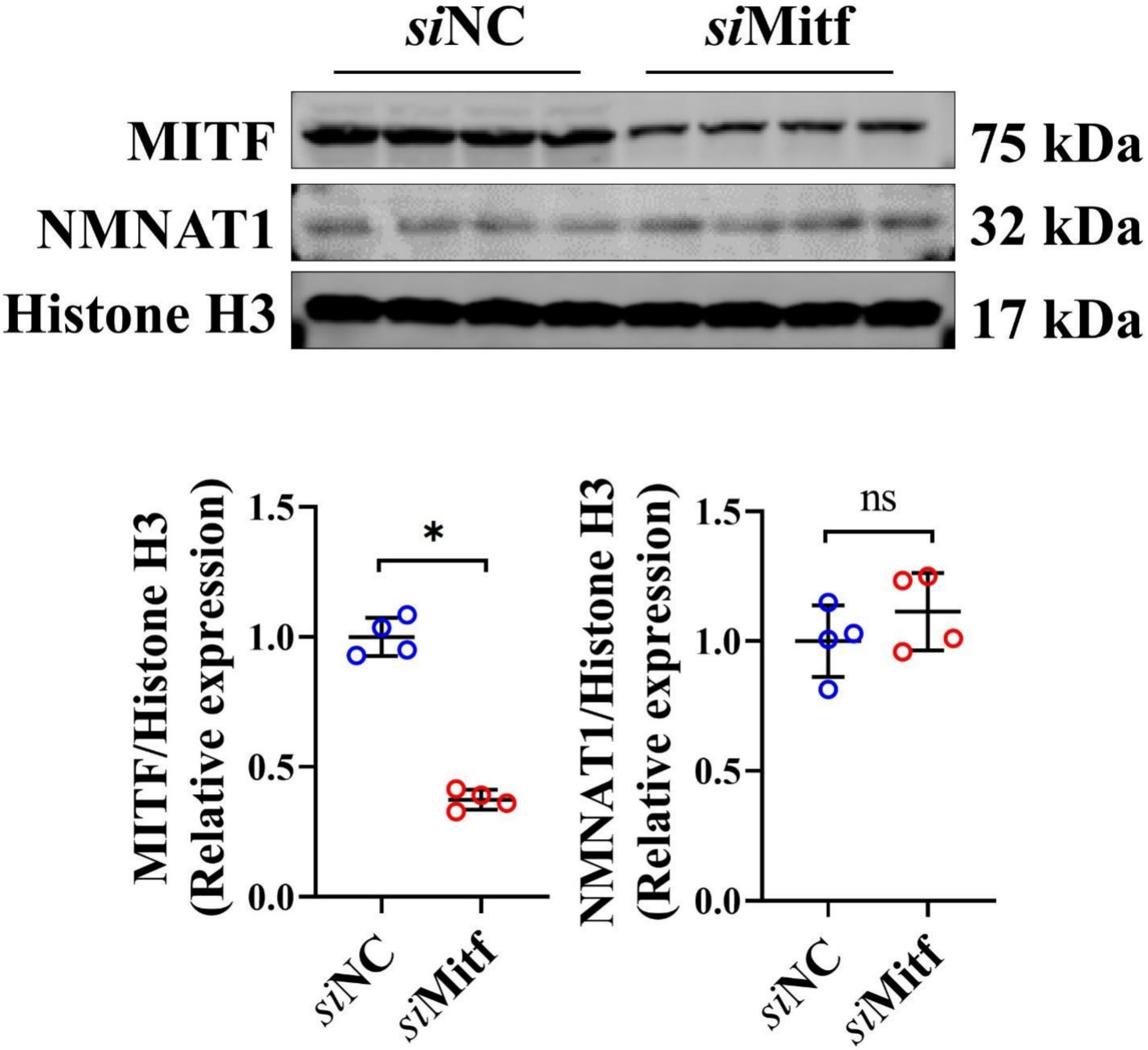

Figure S6. NMNAT1 expression was not affected by *Mitf* knockdown in hepatocytes. AML-12 cells were transfected with either control siRNA (*si*NC) or MITF siRNA (*si*Mitf) for 48 h. The intracellular MITF and nuclear NMNAT1 protein expressions were detected (n = 4). Protein band intensity was quantified by ImageJ. Data are presented as means ± SD. *$P < 0.05$ represents statistical difference; ns, represents no statistical difference.

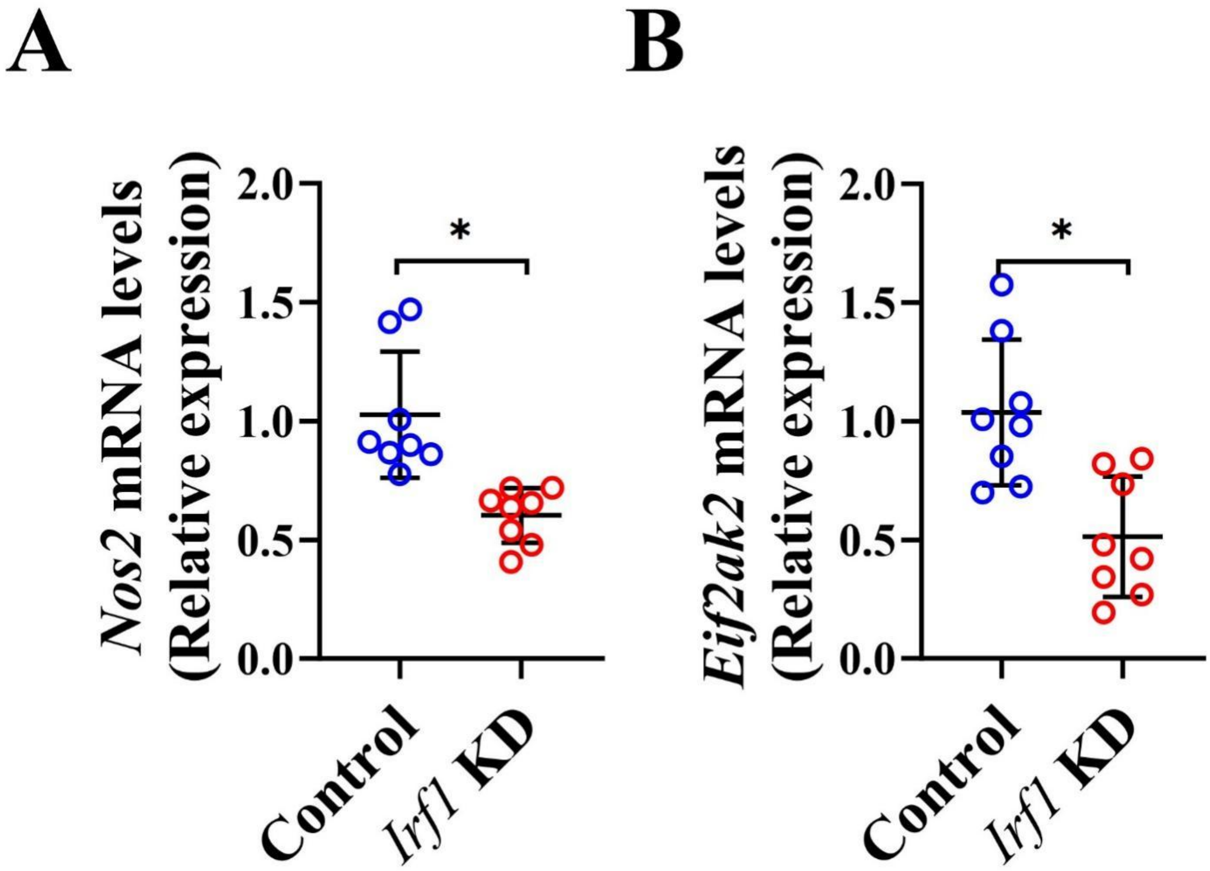

Figure S7. The mRNA expressions of *Nos2* and *Eif2ak2* were decreased in the liver of hepatic *Irf1* knockdown mice. (A) The *Nos2* mRNA expression (n = 8). (B) The *Eif2ak2* mRNA expression (n = 8). Data are presented as means ± SD. *$P < 0.05$ represents statistical difference. *Irf1* KD, liver specific *Irf1*-knockdown mice.

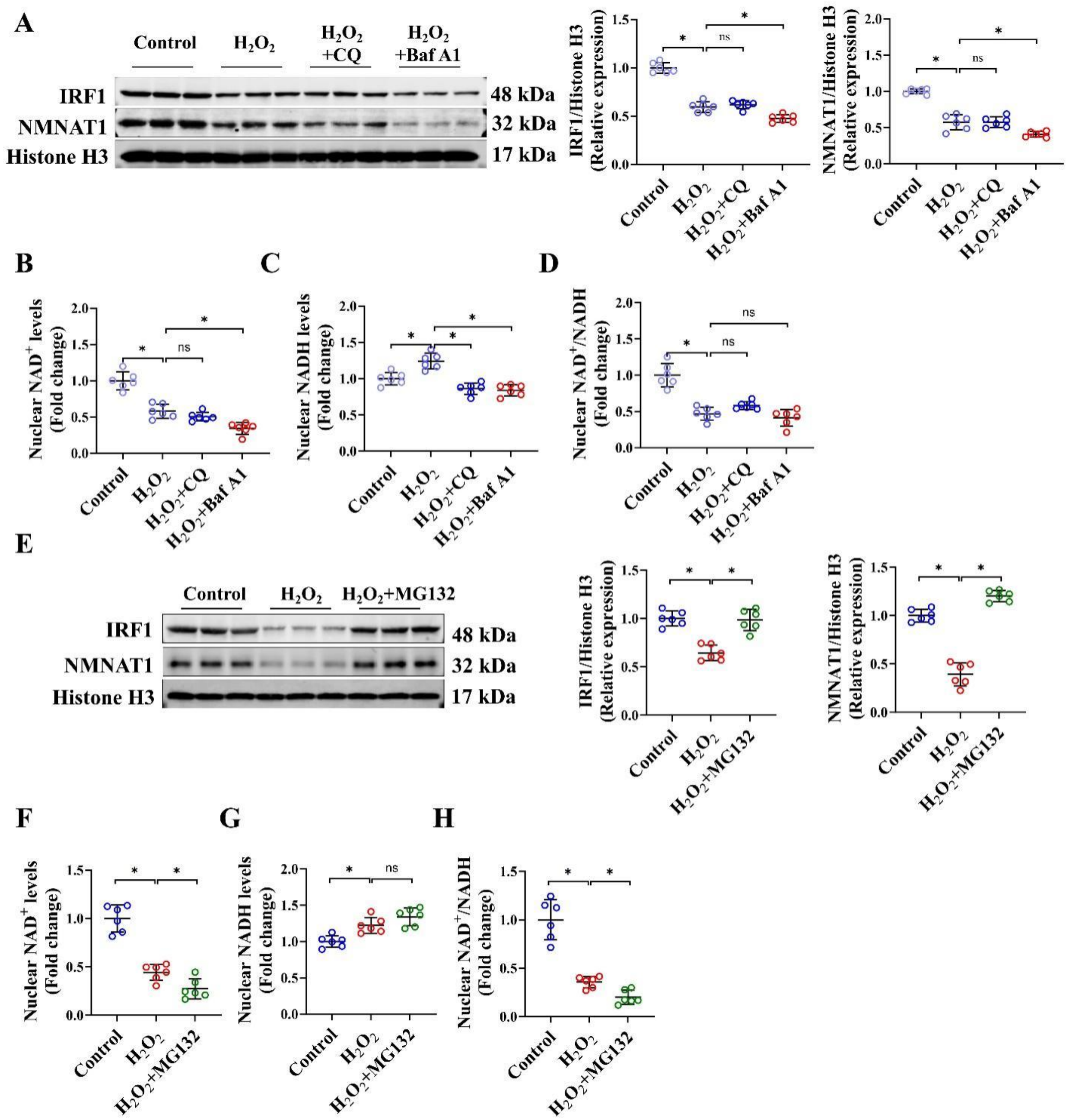

Figure S8. H_2O_2-induced decrease of nuclear IRF1 was inhibited by MG132 treatment in hepatocytes. (A-D) AML-12 cells were treated with H_2O_2 (0.1 mM) for 16 h with or without a 2 h pretreatment with CQ (10 μM) and Baf A1 (10 μM). Nuclear IRF1 and NMNAT1 protein expressions were detected by Western-blot (A, n = 6); nuclear NAD^+, NADH, and NAD^+/NADH ratio were measured/calculated (B-D, n = 6). (E-H) AML-12 cells were treated with H_2O_2 (0.2 mM) for 6 h with or without a 1 h pretreatment with MG132 (10 μM). Nuclear IRF1 and

NMNAT1 protein expressions were detected by Western-blot (E, n = 6); nuclear NAD^+, NADH, and NAD^+/NADH ratio were measured/calculated (F-H, n = 6). Protein band intensity was quantified by ImageJ. Data are presented as means ± SD. *$P < 0.05$ represents statistical difference; ns represents no statistical difference.

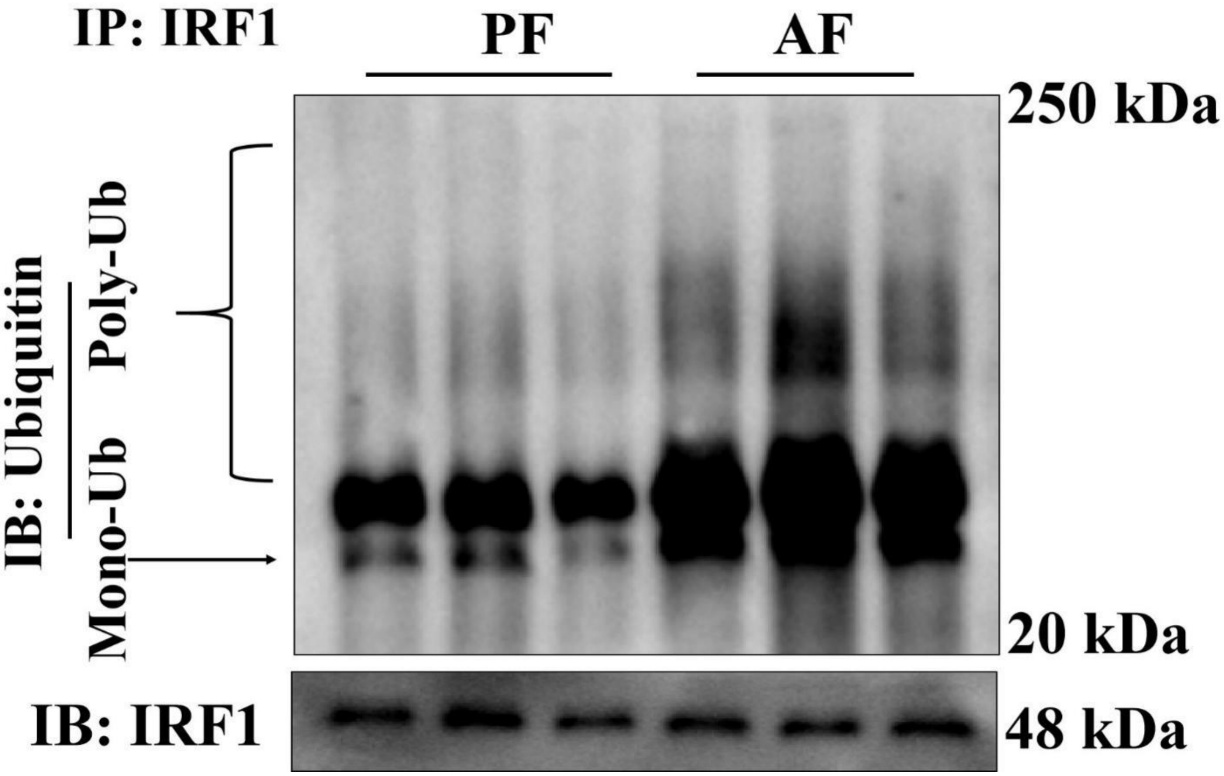

Figure S9. Chronic alcohol consumption induced increase of IRF1 ubiquitination. The ubiquitinated-IRF1 content was detected in the liver of alcohol-fed mice (n = 3). Data are presented as means ± SD. *$P < 0.05$ represents statistical difference. PF, pair-fed; AF, alcohol-fed.

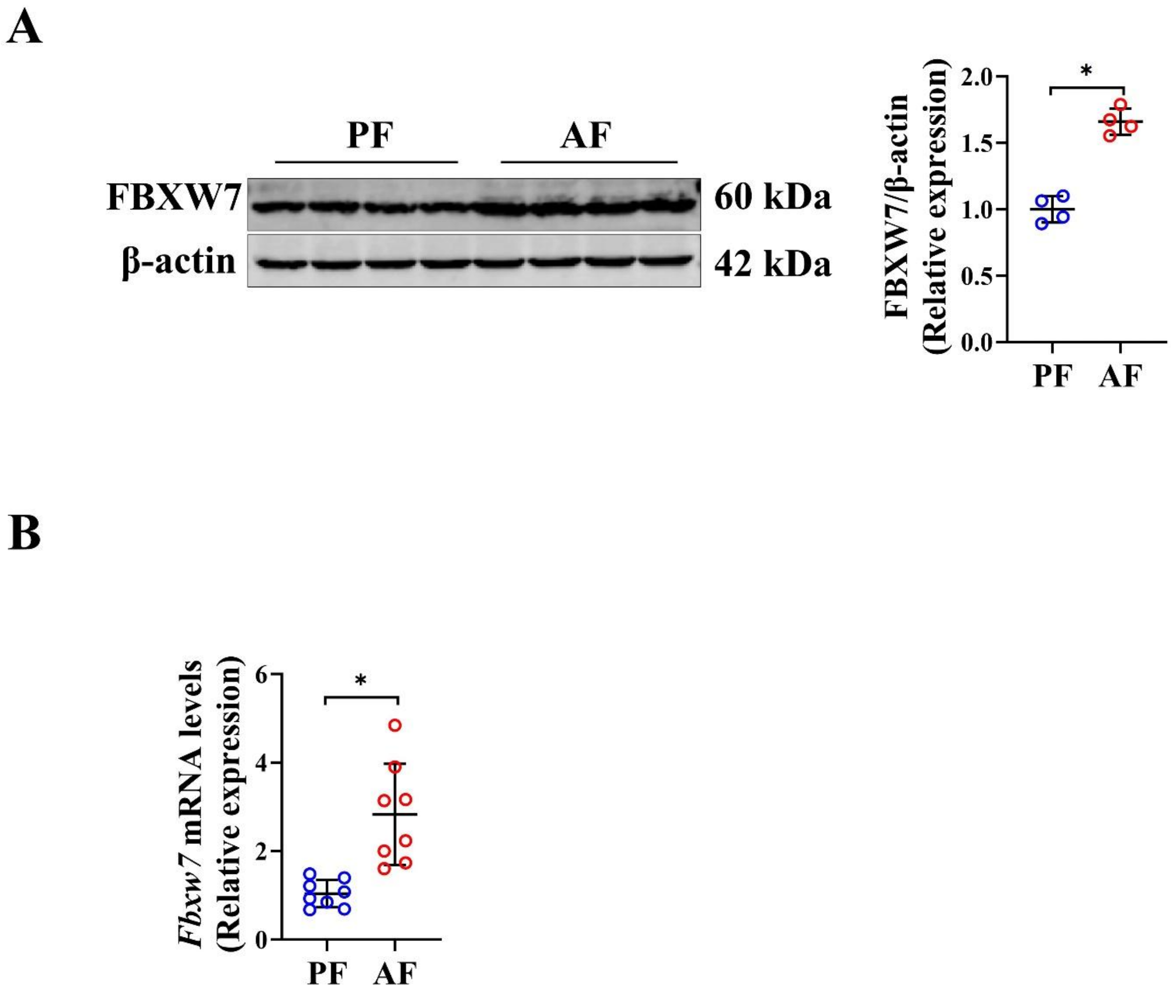

Figure S10. Chronic alcohol consumption induced increase of hepatic FBXW7 expression. (A) Total protein was extracted from liver tissues. The FBXW7 protein expression was detected by Western blot (n = 4). (B) The *Fbxw7* mRNA expression in the liver (n = 8). Protein band intensity was quantified by ImageJ. Data are presented as means ± SD. *$P < 0.05$ represents statistical difference. PF, pair-fed; AF, alcohol-fed.

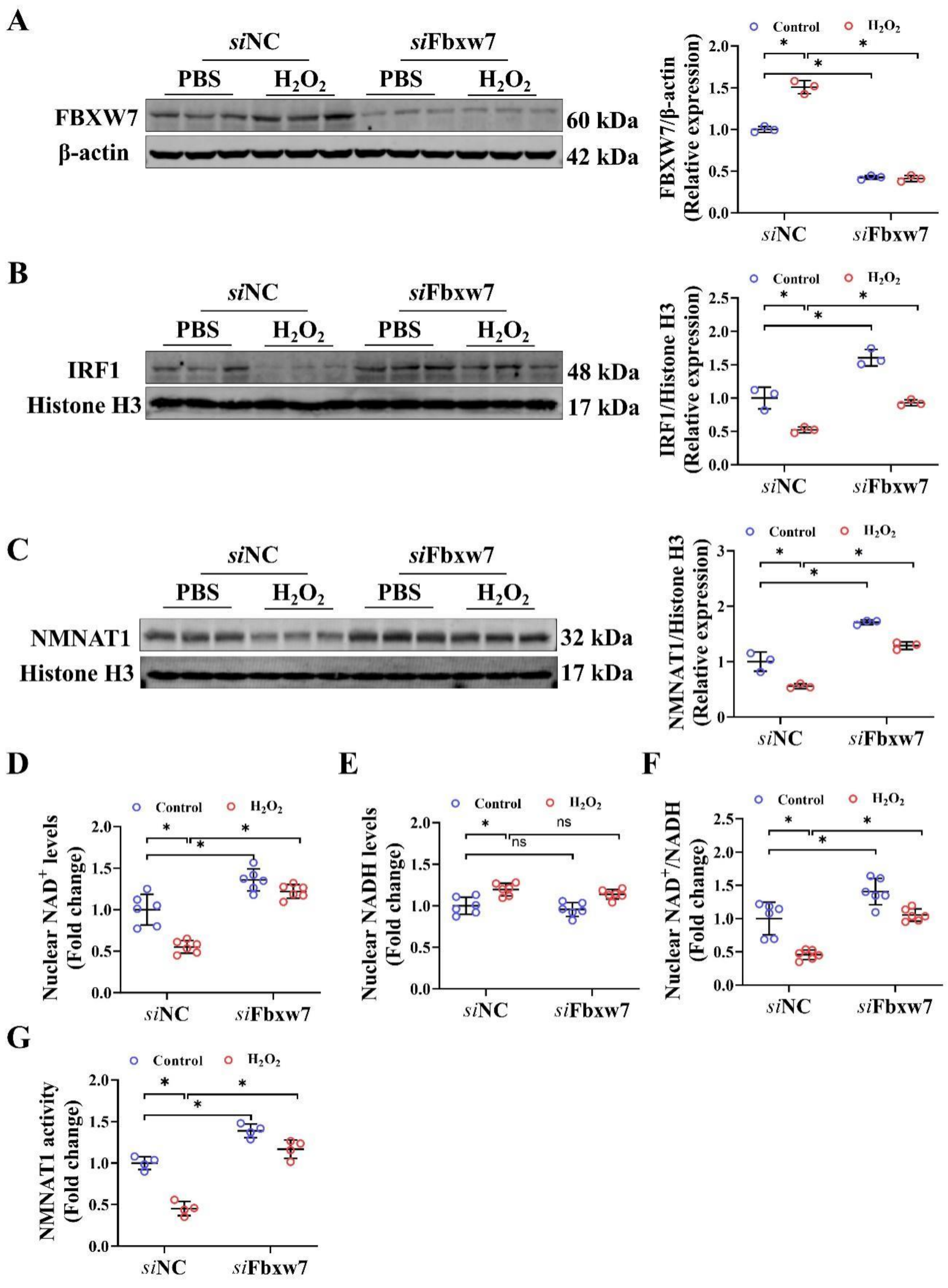

Figure S11. FBXW7 knockdown inhibited H_2O_2-decreased IRF1 and NMNAT1 expression in hepatocytes. AML-12 cells were transfected with either control siRNA (*si*NC) or FBXW7 siRNA (*si*FBXW7) for 48 h and treated with H_2O_2 (0.1 mM) for 24 h. The intracellular FBXW7

(A), nuclear IRF1 (B), and nuclear NMNAT1 (C) protein expressions were detected by Western-blot, respectively (n = 3). (D-F) Nuclear NAD^+, NADH, and NAD^+/NADH ratio were measured/calculated (n = 6). (G) NMNAT1 activity was measured (n = 4). Protein band intensity was quantified by ImageJ. Data are presented as means ± SD. *$P < 0.05$ represents statistical difference.

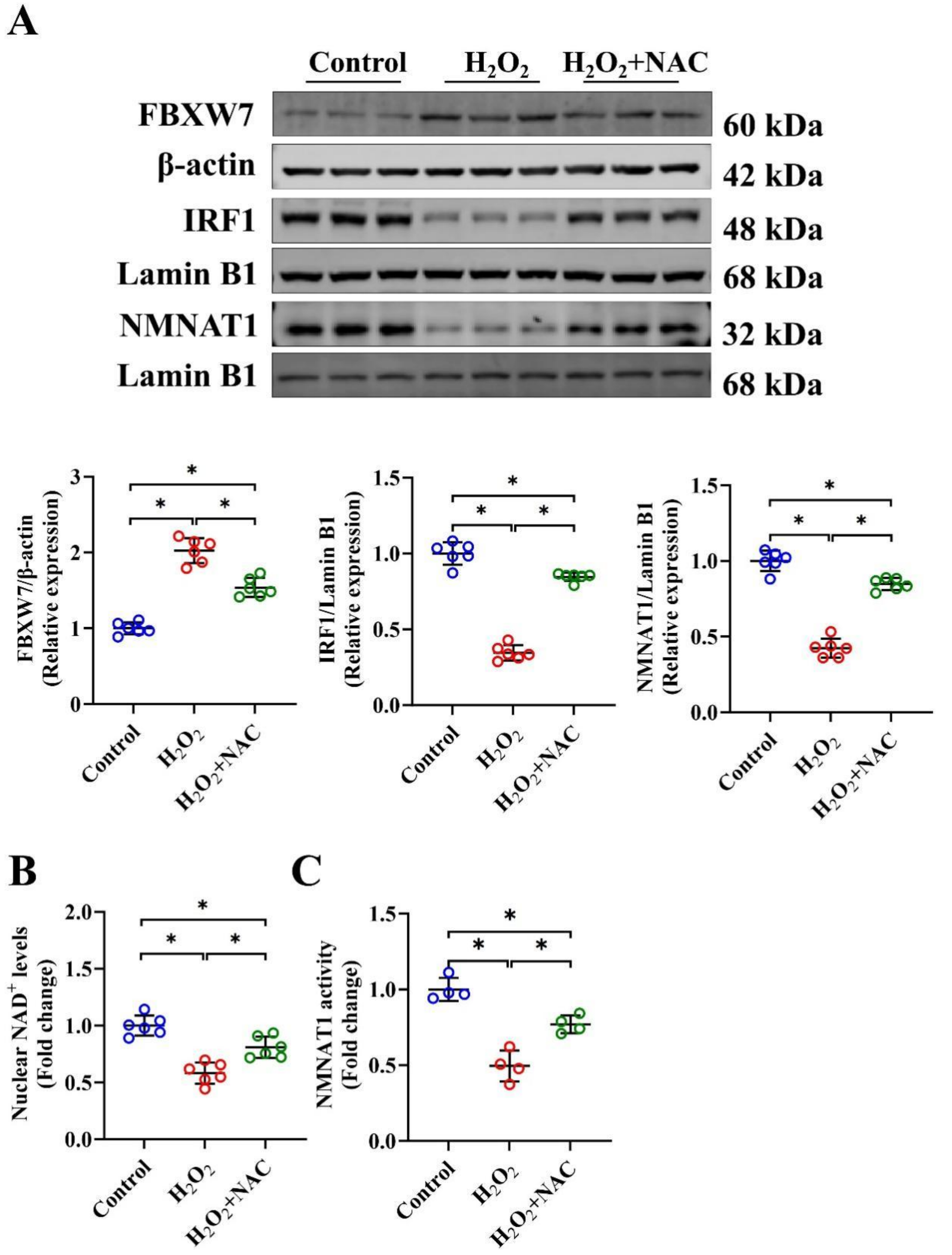

Figure S12. NAC treatment alleviated H_2O_2-induced alterations in FBXW7, IRF1, and NMNAT1 in hepatocytes. AML-12 cells were treated with H_2O_2 (0.1 mM) for 24 h with or without 2 h NAC (2 mM) pretreatment. (A) Intracellular FBXW7, nuclear IRF1, and nuclear NMNAT1 protein expressions were detected by Western-blot, respectively (n = 6). (B) Nuclear

NAD^+ content (n = 6). (C) NMNAT1 activity was measured (n = 4). Protein band intensity was quantified by ImageJ. Data are presented as means ± SD. *$P < 0.05$ represents statistical difference. NAC, N-acetylcysteine.

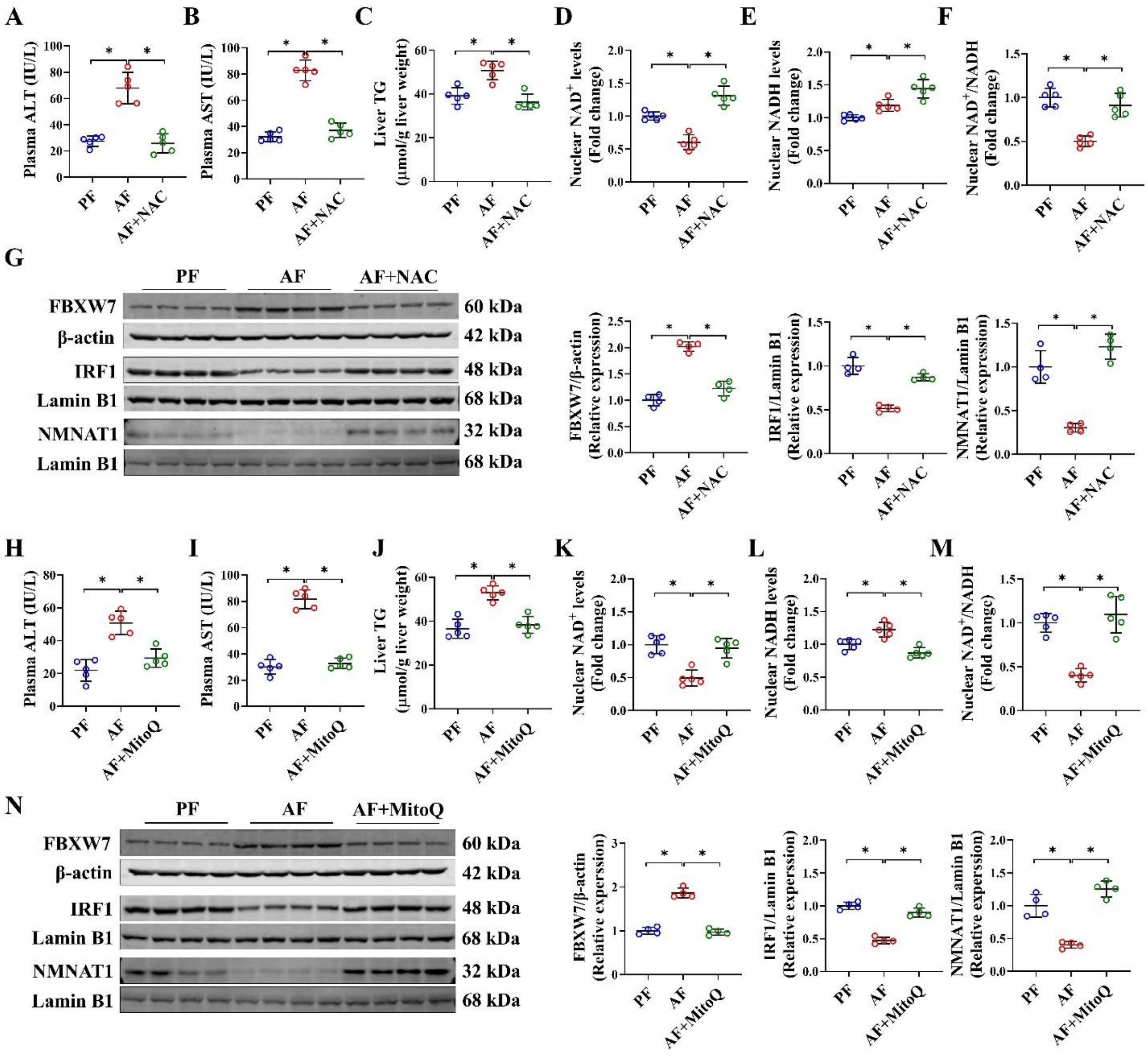

Figure S13. Antioxidants administration inhibited chronic alcohol-induced alterations in the expressions of hepatic FBXW7, IRF1, and NMNAT1, as well as the hepatic nuclear NAD^+ and NADH levels in mice. (A-G) NAC (40 mg/kg/day) was administrated to chronic alcohol-fed mice as described in the Methods. (A) Plasma ALT levels (n = 5). (B) Plasma AST levels (n = 5). (C) Liver total triglyceride (TG) levels (n = 5). (D) Nuclear NAD^+ content (n = 5). (E) Nuclear NADH content (n = 5). (F) Nuclear NAD^+/NADH ratio (n = 5). (G) total FBXW7, nuclear IRF1, and nuclear NMNAT1 protein expressions were detected by Western-blot, respectively (n = 4). (H-N) MitoQ (5 mg/kg/day) was administrated to chronic alcohol-fed mice as described in the Methods. (H) Plasma ALT levels (n = 5). (I) Plasma AST levels (n = 5). (J)

Liver total triglyceride (TG) levels (n = 5). (K) Nuclear NAD^+ content (n = 5). (L) Nuclear NADH content (n = 5). (M) Nuclear NAD^+/NADH ratio (n = 5). (N) total FBXW7, nuclear IRF1, and nuclear NMNAT1 protein expressions were detected by Western-blot, respectively (n = 4). Protein band intensity was quantified by ImageJ. Data are presented as means ± SD. $^*P < 0.05$ represents statistical difference. PF, pair-fed; AF, alcohol-fed; NAC, N-acetylcysteine; MitoQ, mitoquinone.

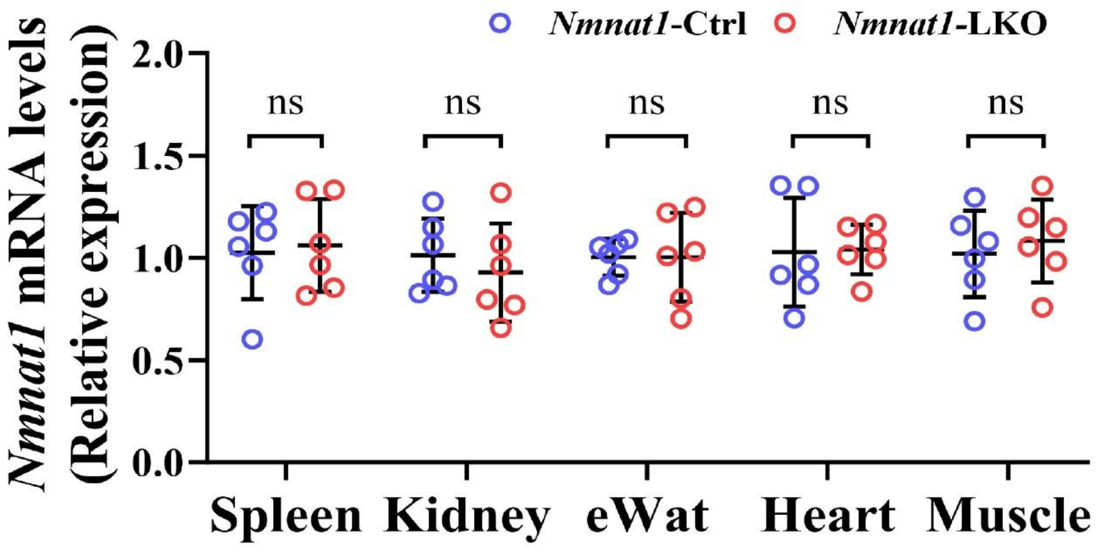

Figure S14. The *Nmnat1* expression was not affect in the tested organs of hepatic NMNAT1 knockout mice. The *Nmnat1* mRNA expression was detected in the spleen, kidney, epididymal white fat, heart, and muscle tissues (n = 6). Data are presented as means ± SD. ns represents no statistical difference.

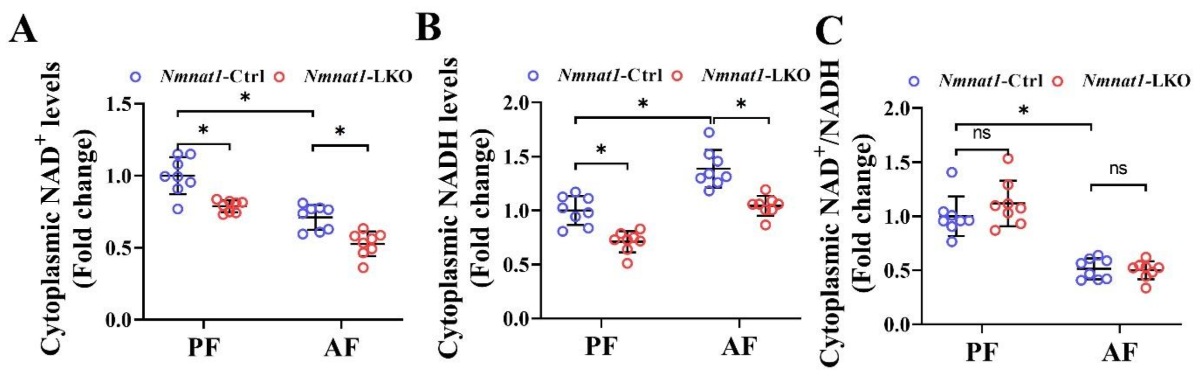

Figure S15. Cytoplasmic NAD$^+$ was decreased in the liver of NMNAT1-LKO mice. (A) Cytoplasmic NAD$^+$ content (n = 8). (B) Cytoplasmic NADH content (n = 8). (C) Cytoplasmic NAD$^+$/NADH ratio (n = 8). Data are presented as means ± SD. *$P < 0.05$ represents statistical difference; ns represents no statistical difference. PF, pair-fed; AF, alcohol-fed.

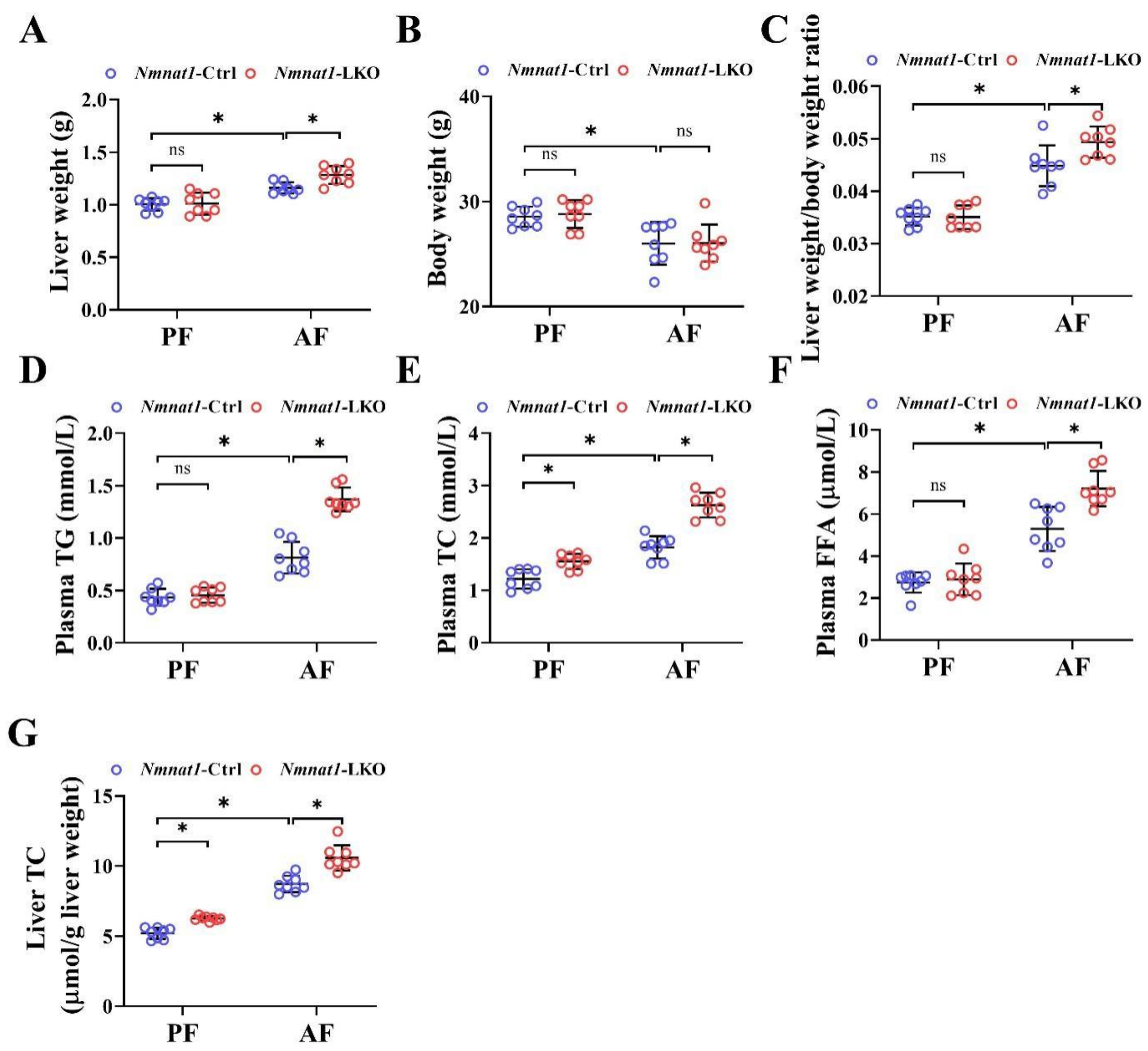

Figure S16. Hepatic NMNAT1 loss aggravated chronic alcohol-induced liver weight gain and dyslipidemia in mice. (A) Liver weight (n = 8). (B) Body weight (n = 8). (C) Liver weight/body weight ratio (n = 8). (D) Plasma total triglyceride (TG) levels (n = 8). (E) Plasma TC levels (n = 8). (F) Plasma FFA levels (n = 8). (G) Liver TC levels (n = 8). Data are presented as means ± SD. **P* < 0.05 represents statistical difference; ns represents no statistical difference. PF, pair-fed; AF, alcohol-fed.

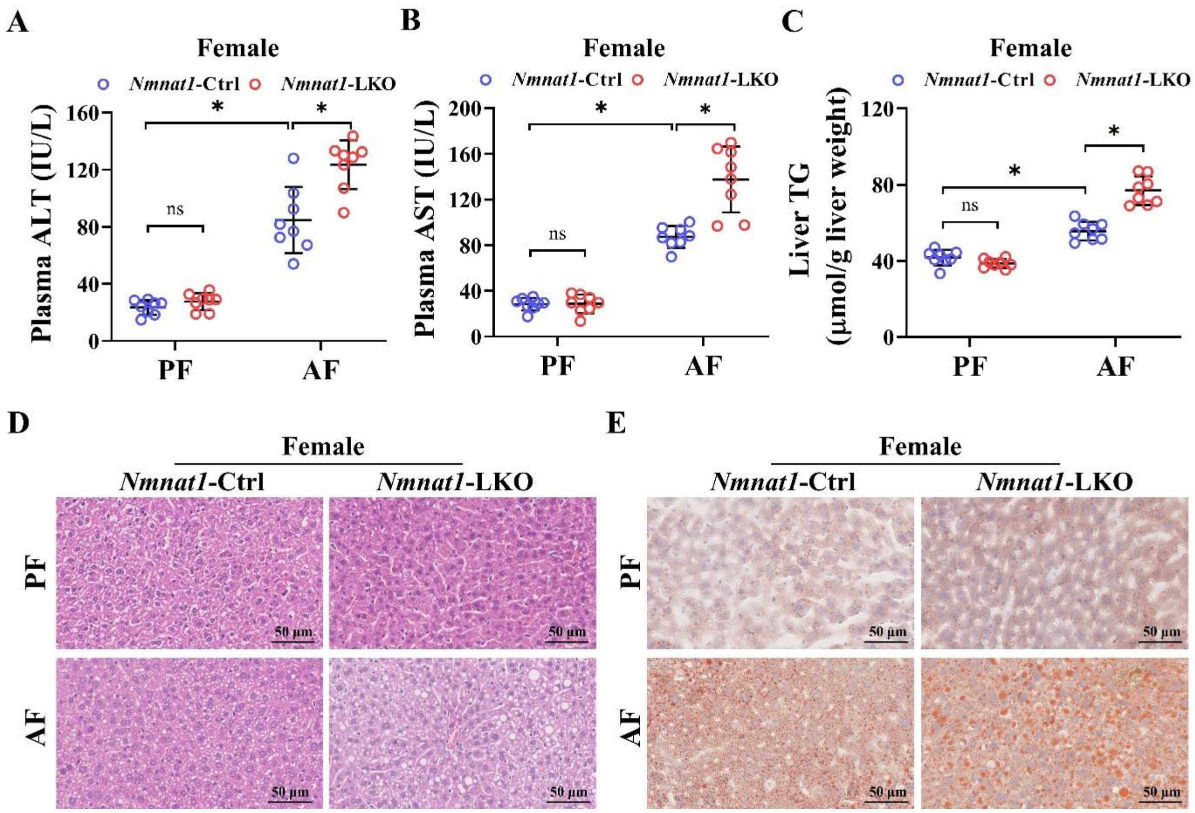

Figure S17. Hepatic NMNAT1 knockout exacerbated chronic alcohol-induced liver injury and lipid deposition in female C57BL/6N mice. Female *Nmnat1*-Ctrl and *Nmnat1*-LKO mice were pair-fed or alcohol-fed for 8-week plus a single binge of ethanol (5 g/kg). Samples were collected 4 hours after the single binge. (A) Plasma ALT levels (n = 8). (B) Plasma AST levels (n = 8). (C) Liver total triglyceride (TG) levels (n = 8). (D) Liver H&E staining (n = 4). (E) Liver Oil red O staining (n = 4). Data are presented as means ± SD. *$P < 0.05$ represents statistical difference; ns represents no statistical difference. PF, pair-fed; AF, alcohol-fed.

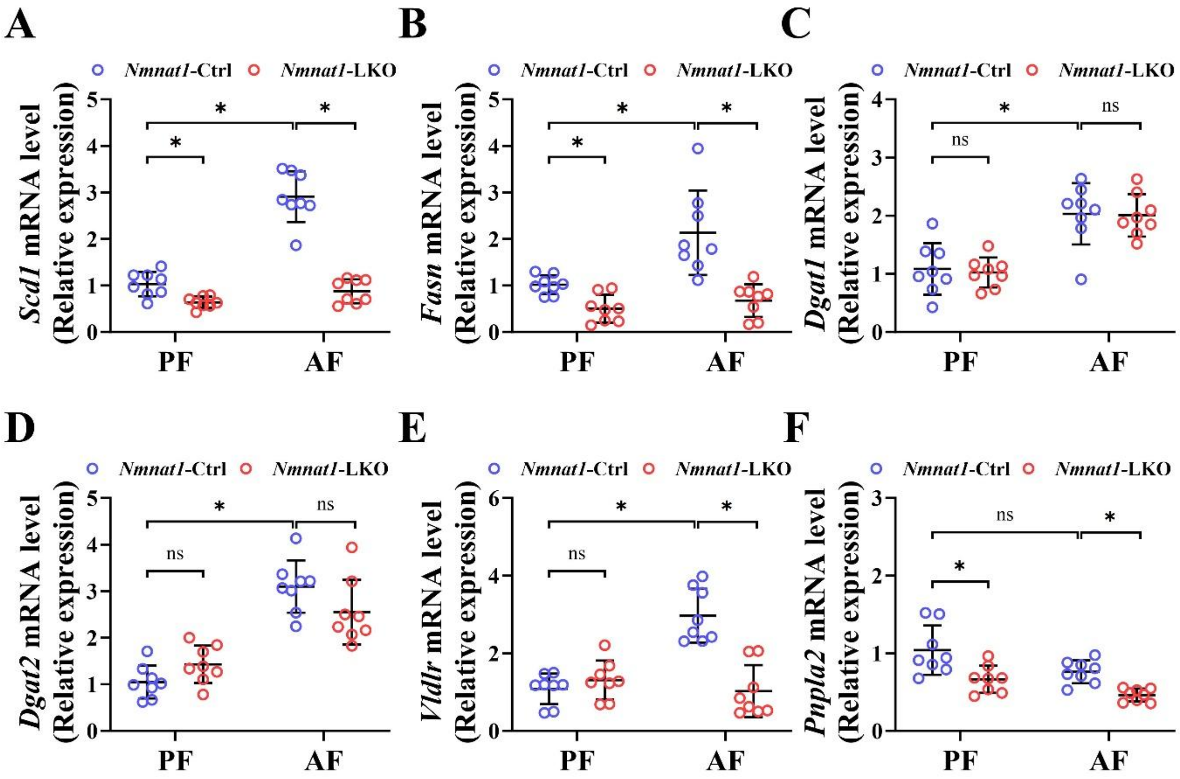

Figure S18. Hepatic NMNAT1 knockout disrupted the expression of lipid metabolism related genes. (A-F) The mRNA expressions of *Scd1, Fasn, Dgat1, Dgat2, Vldlr, and Pnpla2* in mice liver were detected by qRT-PCR, respectively (n = 8). Data are presented as means ± SD. *$P < 0.05$ represents statistical difference; ns represents no statistical difference. PF, pair-fed; AF, alcohol-fed.

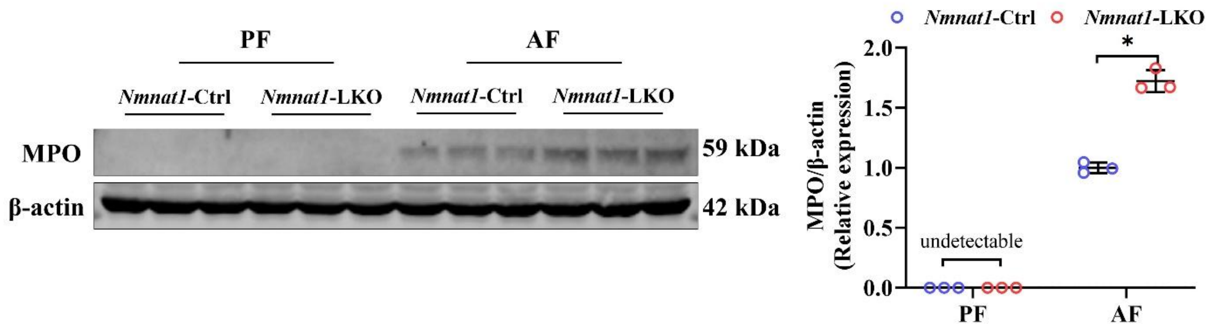

Figure S19. Hepatic NMNAT1 knockout exacerbated alcohol-induced hepatic MPO expression increase. The MPO protein expression in mice liver was detected by Western-blot (n = 3). Protein band intensity was quantified by ImageJ. Data are presented as means ± SD. *$P < 0.05$ represents statistical difference. PF, pair-fed; AF, alcohol-fed.

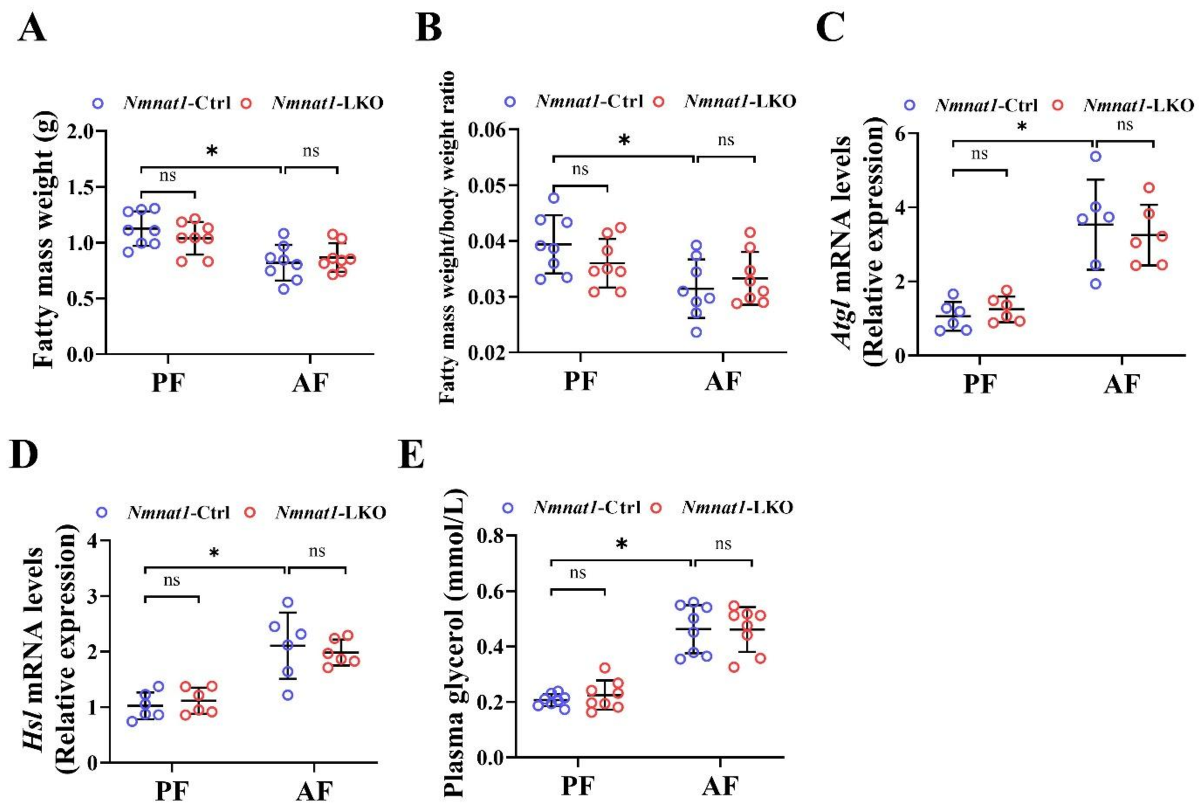

Figure S20. Alcohol-induced lipolysis was not enhanced in hepatic NMNAT1 knockout mice. (A) Fatty mass weight (n = 8). (B) Fatty mass weight/body weight ratio (n = 8). (C & D) The mRNA expressions of *Atgl* and *Hsl* in mice epididymal white fat (n = 6). (E) Plasma glycerol levels (n = 8). Data are presented as means ± SD. *$P < 0.05$ represents statistical difference; ns represents no statistical difference. PF, pair-fed; AF, alcohol-fed.

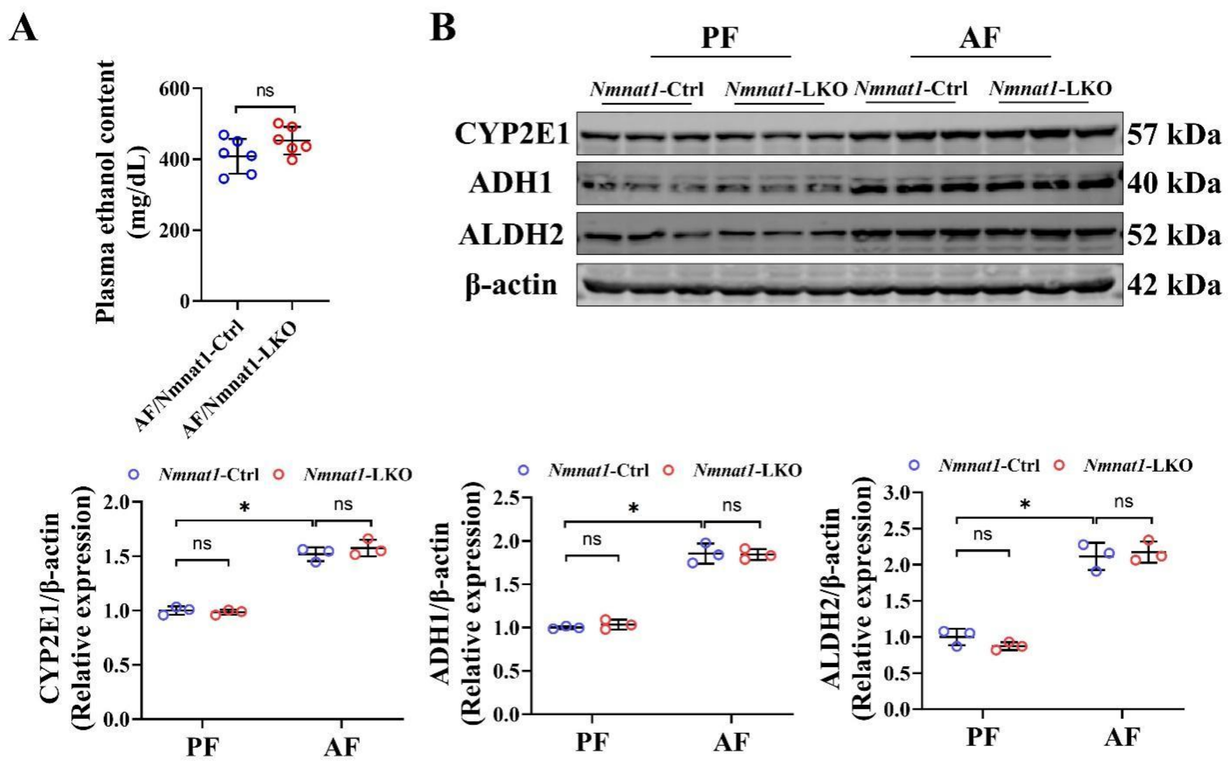

Figure S21. Alcohol metabolism was irrelevant to hepatic NMNAT1 in mice. (A) Plasma ethanol content (n = 6). (B) Cytoplasmic CYP2E1, ADH1, and ALDH2 protein expressions were detected by Western-blot, respectively (n = 3). Protein band intensity was quantified **by ImageJ.** Data are presented as means ± SD. *$P < 0.05$ represents statistical difference; ns represents no statistical difference. PF, pair-fed; AF, alcohol-fed.

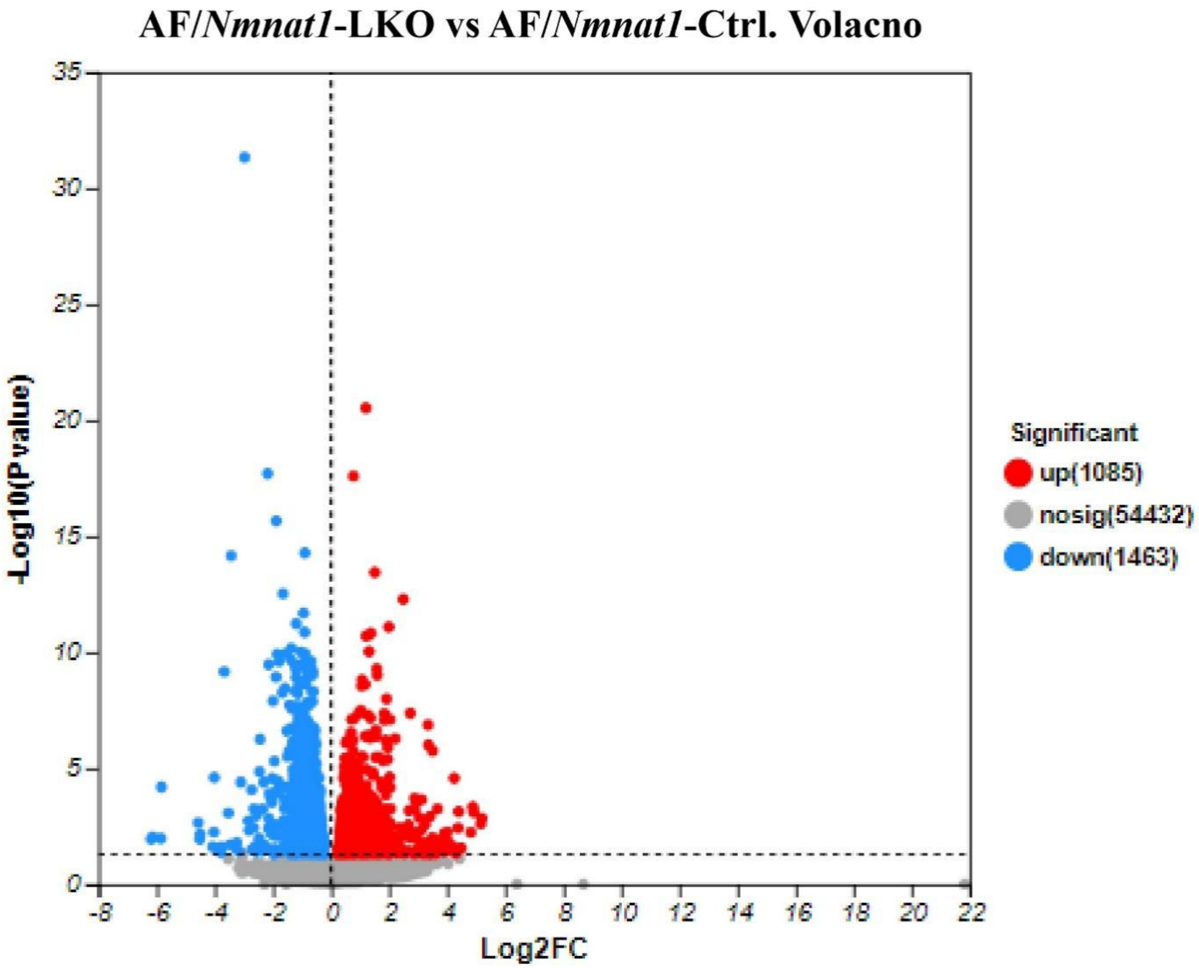

Figure S22. RNA sequencing analysis showed the number of differentially expressed genes in the liver between alcohol-fed NMNAT1-LKO mice and its littermate control mice. The volcano map showed differentially expressed genes in the liver of alcohol-fed *Nmnat1*-Ctrl and *Nmnat1*-LKO mice (n = 4). AF, alcohol-fed.

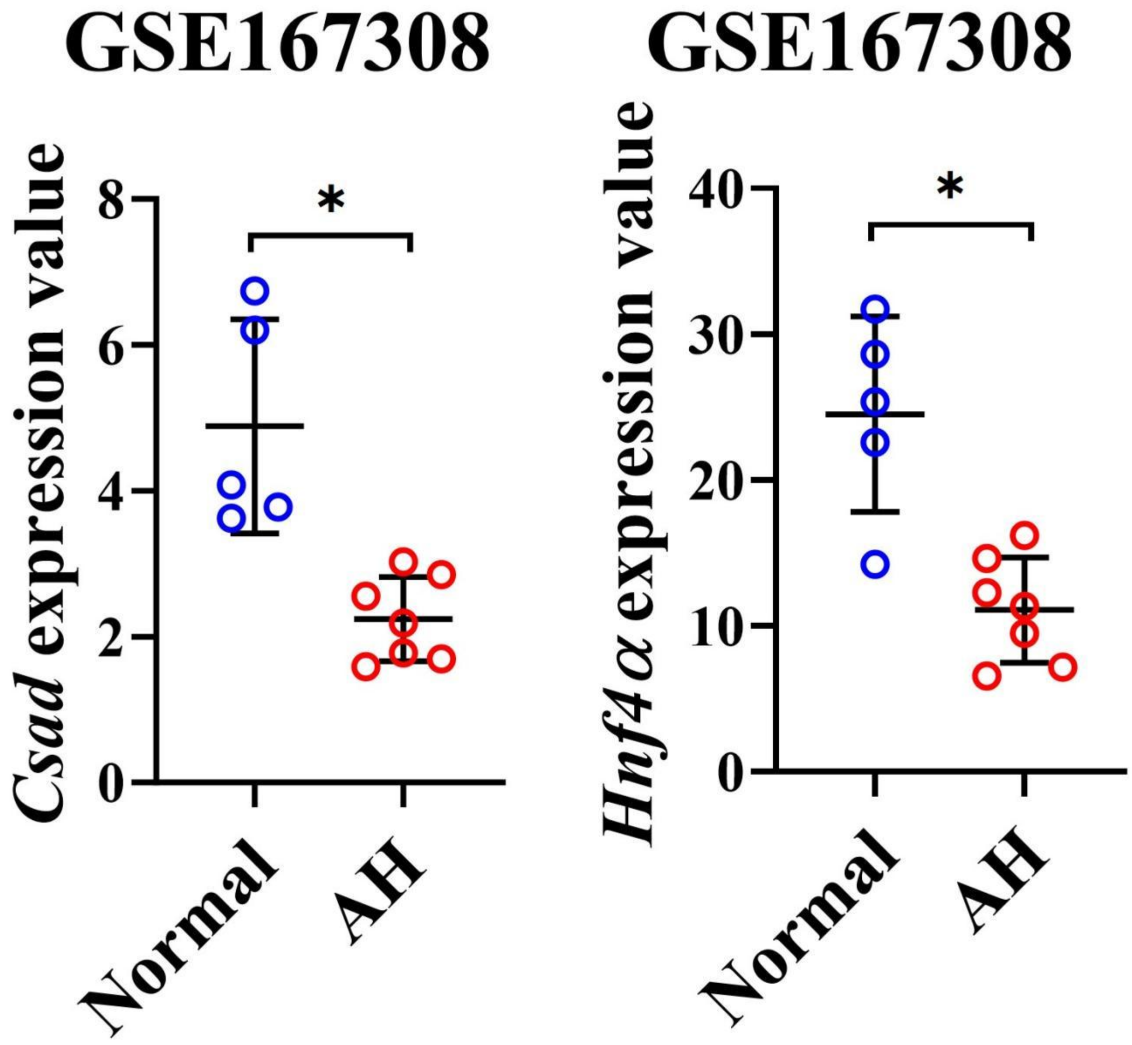

Figure S23. Hepatic *Csad* and *Hnf4α* gene expressions were decreased in the patients with alcoholic steatohepatitis (ASH). The GEO public database analyzes hepatic *Csad* and *Hnf4α* gene expression in ASH patients. **The hepatic** *Csad* and *Hnf4α* **gene expressions in the liver samples from normal human (n = 5) and ASH patients (n = 7) was analyzed based on the GEO database (GSE167308) from NCBI using its online software GEO2R.** Data are presented as means ± SD. *$P < 0.05$ represents statistical difference.

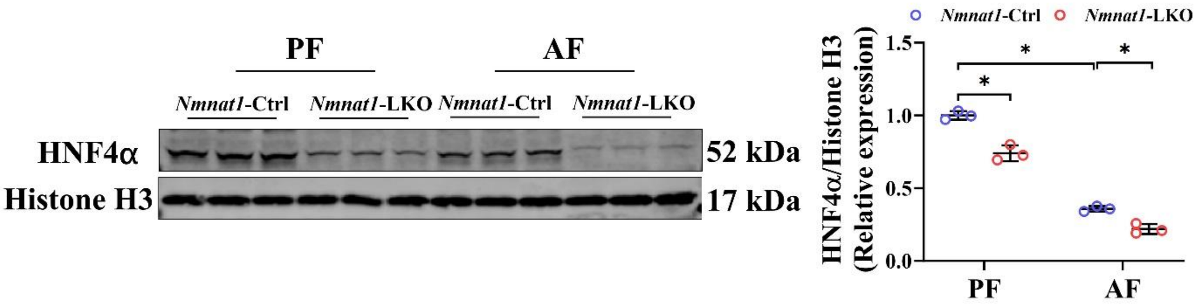

Figure S24. Hepatic NMNAT1 knockout aggregated chronic alcohol-induced downregulation of hepatic HNF4α expression. The nuclear HNF4α **protein expression in the liver samples were detected by Western-blot (n = 3).** **Protein band intensity was quantified by ImageJ.** Data are presented as means ± SD. *$P < 0.05$ represents statistical difference. PF, pair-fed; AF, alcohol-fed.

A

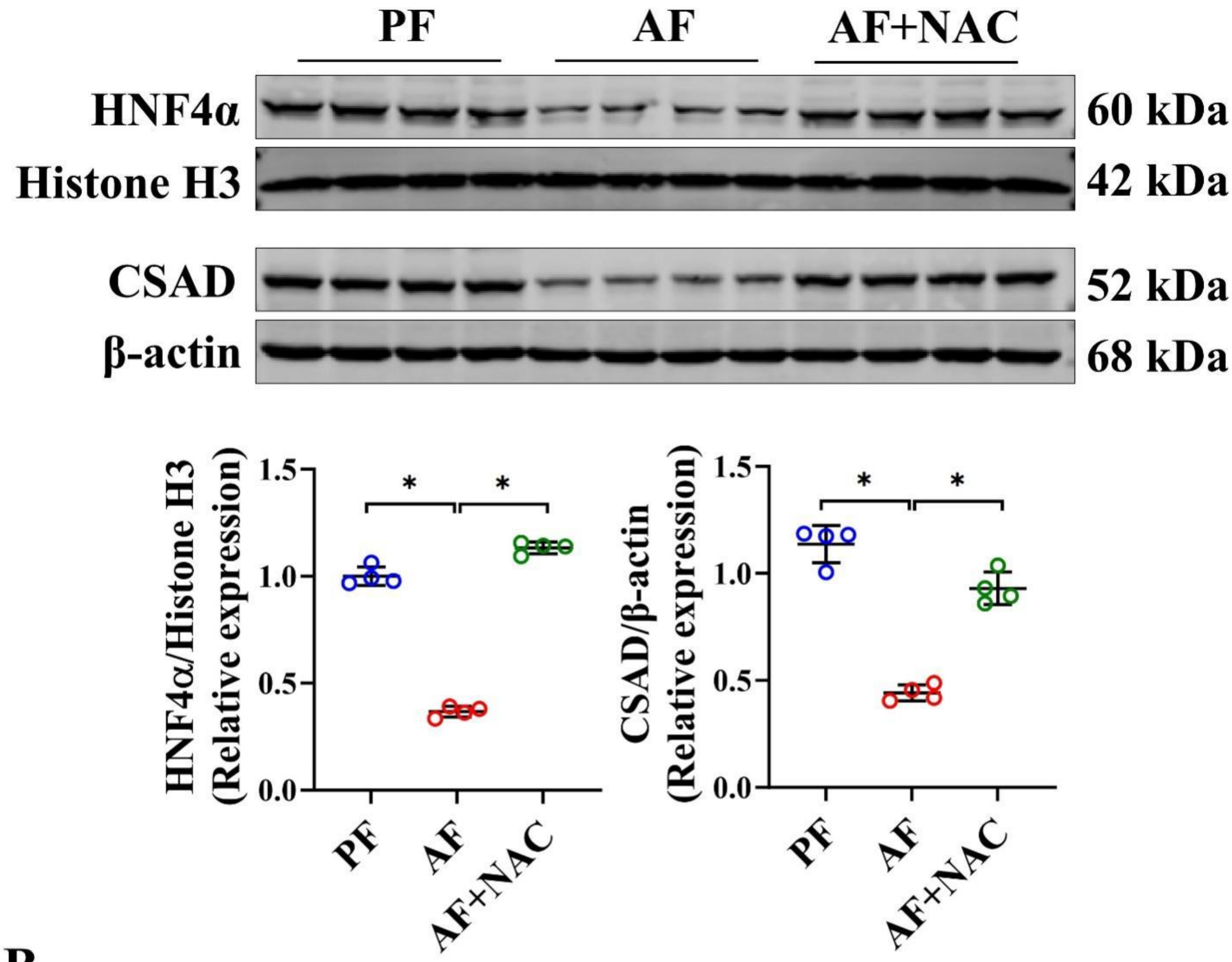
PF
AF
AF+NAC
HNF4α
Histone H3
CSAD
β-actin
60 kDa
42 kDa
52 kDa
68 kDa
HNF4α/Histone H3
(Relative expression)
CSAD/β-actin
(Relative expression)
1.5
1.0
0.5
0.0
*
PF
AF
AF+NAC

B

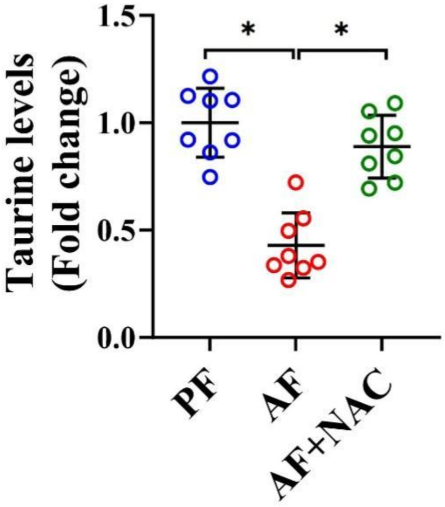
Taurine levels
(Fold change)
1.5
1.0
0.5
0.0
*
PF
AF
AF+NAC

Figure S25. NAC administration rescued chronic alcohol-induced decrease of hepatic HNF4α and CSAD expressions and taurine levels. (A) Nuclear HNF4α and cytoplasmic CSAD protein expressions (n = 4). (B) Taurine levels (n = 8). Protein band intensity was quantified by ImageJ. Data are presented as means ± SD. *$P < 0.05$ represents statistical difference. PF, pair-fed; AF, alcohol-fed, NAC, N-acetylcysteine.

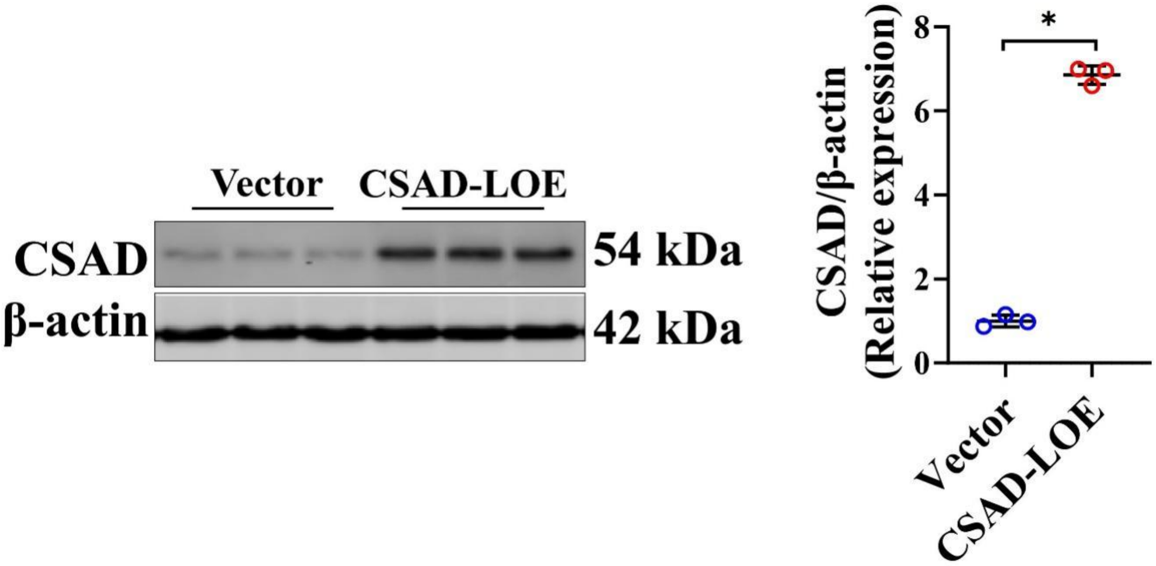

Figure S26. Hepatic CSAD expression was increased in CSAD overexpression (CSAD LOE) mice. Liver-specific CSAD overexpression mice were generated by caudal vein injection with AAV-8 constructed vector containing CSAD sequence into NMNAT1-LKO mice. Mice injected with null-vector were served as control. The protein expression of CSAD was detected in the liver of chronic alcohol-fed NMNAT1-LKO mice from vector control and CSAD-LOE groups (n = 3). P**rotein band intensity was quantified by ImageJ**. Data are presented as means ± SD. **P* < 0.05 represents statistical difference.

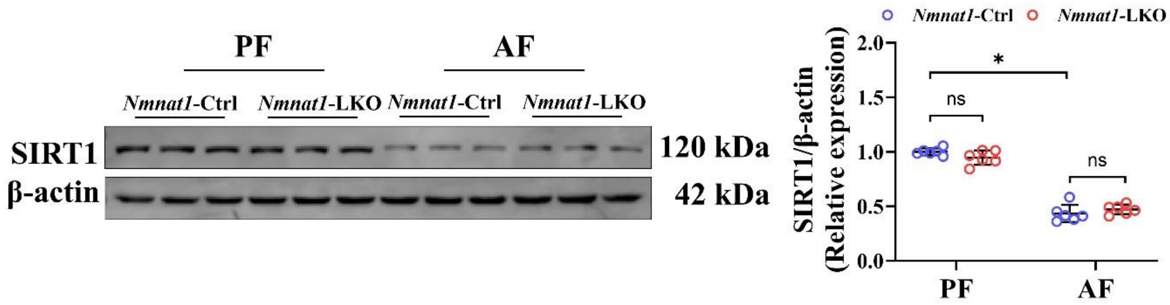

Figure S27. Chronic alcohol-induced decrease of hepatic SIRT1 was not affected by Hepatic *Nmnat1* knockout. The SIRT1 **protein expression in the liver of alcohol-fed *Nmnat1*-Ctrl and *Nmnat1*-LKO mice was detected by Western-blot (n = 6).** Protein band **intensity was quantified by ImageJ.** Data are presented as means ± SD. *P < 0.05 represents statistical difference; ns represents no statistical difference. PF, pair-fed; AF, alcohol-fed.

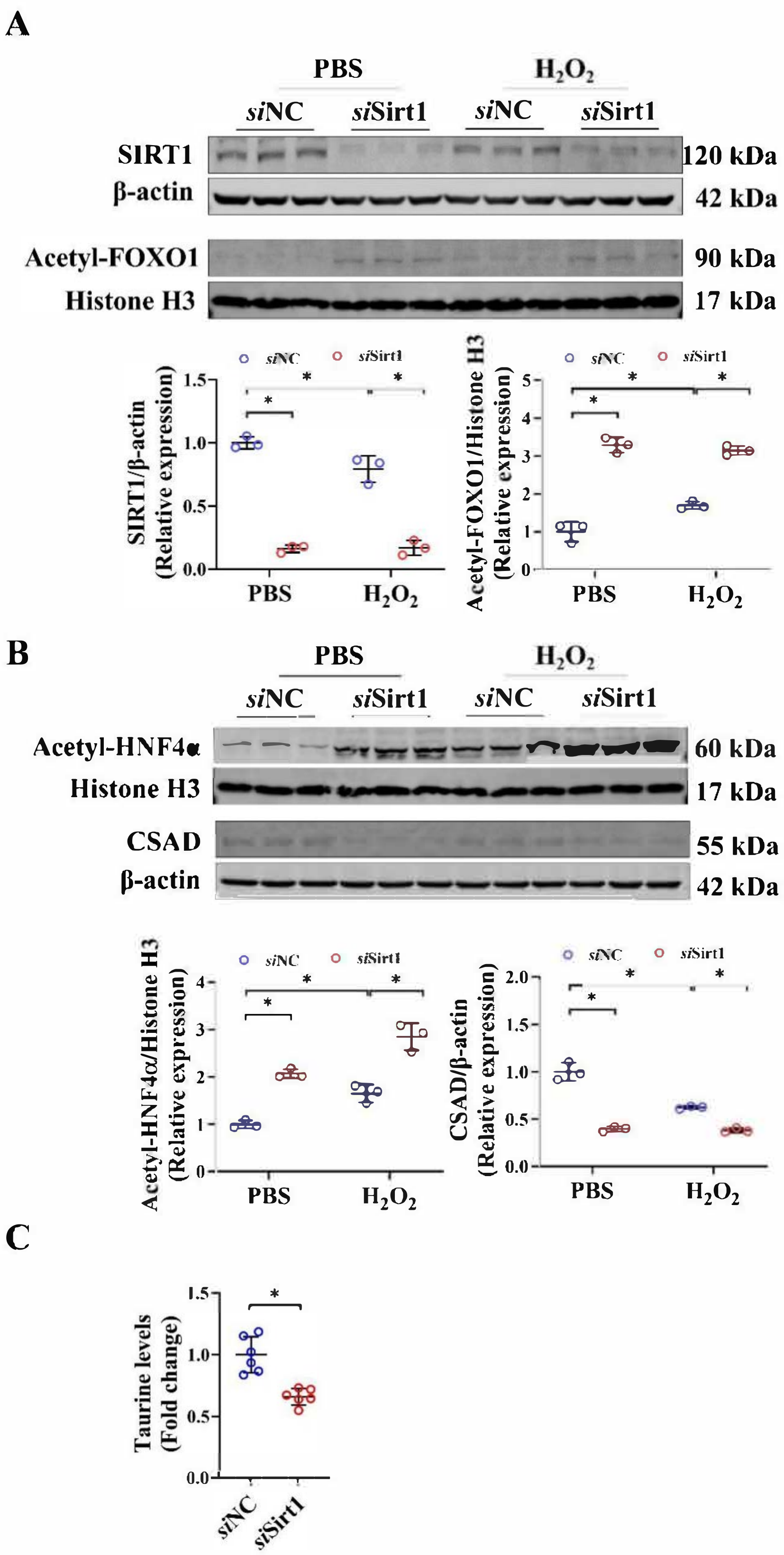

A
PBS
H2O2
siNC
siSirt1
siNC
siSirt1
SIRT1
120 kDa
β-actin
42 kDa
Acetyl-FOXO1
90 kDa
Histone H3
17 kDa
siNC
siSirt1
SIRT1/β-actin (Relative expression)
1.5
1.0
0.5
0.0
PBS
H2O2
Acetyl-FOXO1/Histone H3 (Relative expression)
5
4
3
2
1
0
PBS
H2O2
B
PBS
H2O2
siNC
siSirt1
siNC
siSirt1
Acetyl-HNF4α
60 kDa
Histone H3
17 kDa
CSAD
55 kDa
β-actin
42 kDa
siNC
siSirt1
Acetyl-HNF4α/Histone H3 (Relative expression)
4
3
2
1
0
PBS
H2O2
CSAD/β-actin (Relative expression)
2.0
1.5
1.0
0.5
0.0
PBS
H2O2
C
Taurine levels (Fold change)
1.5
1.0
0.5
0.0
siNC
siSirt1

Figure S28. SIRT1 knockdown aggravated H_2O_2**-induced the increase of HNF4α acetylation and the decrease of CSAD expression and taurine levels in hepatocytes. AML-12 cells were transfected with either control siRNA (*si*NC) or SIRT1 siRNA (*si*SIRT1) for 48 h and treated with** H_2O_2 **(0.1 mM) for 24 h. (A) The total SIRT1 and nuclear** Acetyl-FOXO1 **protein expressions in the AML-12 (n = 3). (B) The nuclear** Acetyl-HNF4α and total CSAD **protein expressions in the AML-12 (n = 3). (C) Taurine levels (n = 6). Protein band intensity was quantified by ImageJ**. Data are presented as means ± SD. $^*P < 0.05$ represents statistical difference.

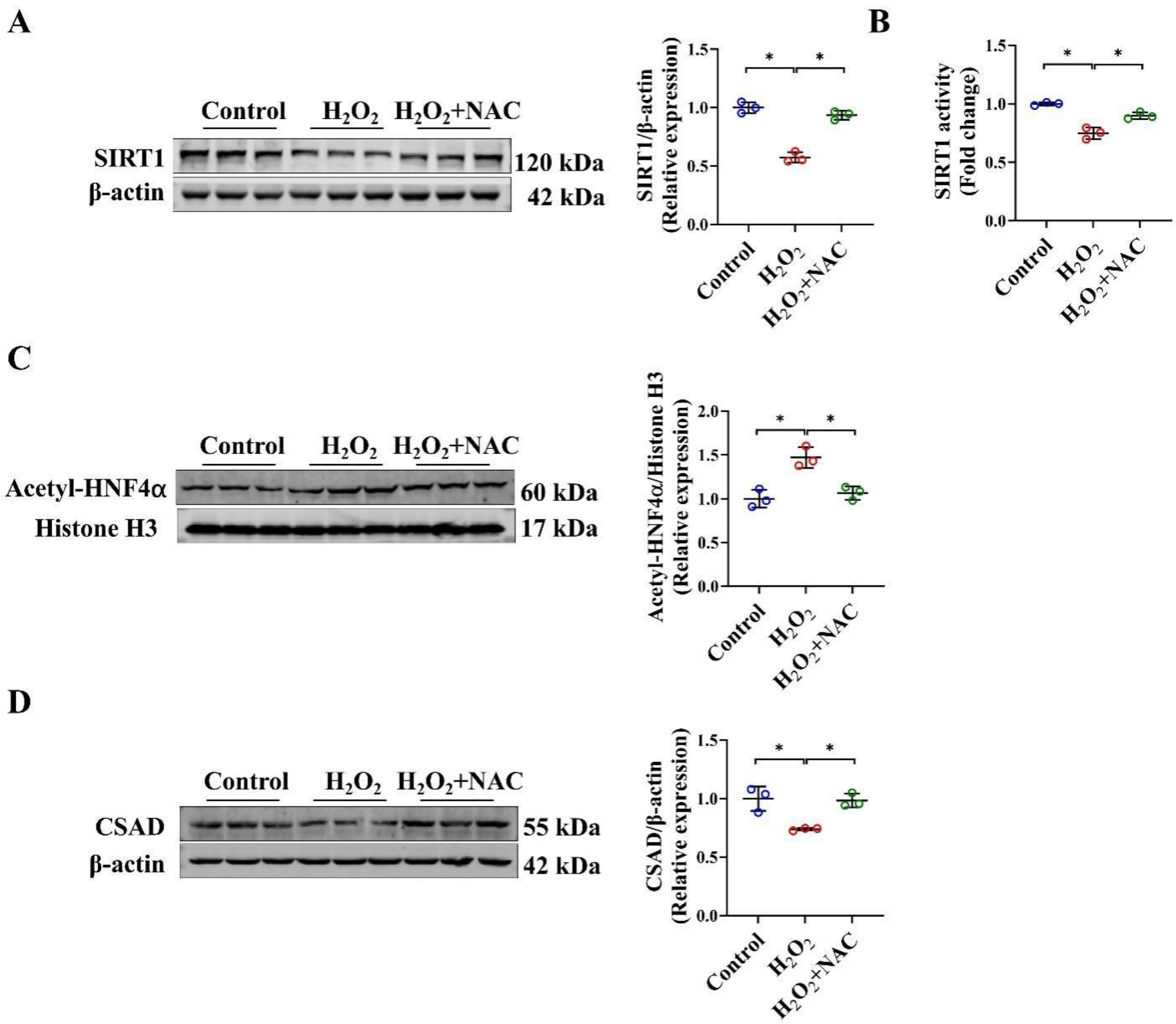

Figure S29. NAC treatment alleviated H_2O_2-induced alterations in SIRT1, Acetyl-HNF4α, and CSAD in hepatocytes. AML-12 cells were treated with H_2O_2 (0.1 mM) for 24 h with or without a 2 h pretreatment with NAC (2 mM). (A & B) SIRT1 protein expressions and activity were measured (n = 3). (C & D) Nuclear Acetyl-HNF4α and total CSAD **protein expressions were measured. (n = 3). Protein band intensity was quantified by ImageJ.** Data are presented as means ± SD. *$P < 0.05$ represents statistical difference. NAC, N-acetylcysteine**.**

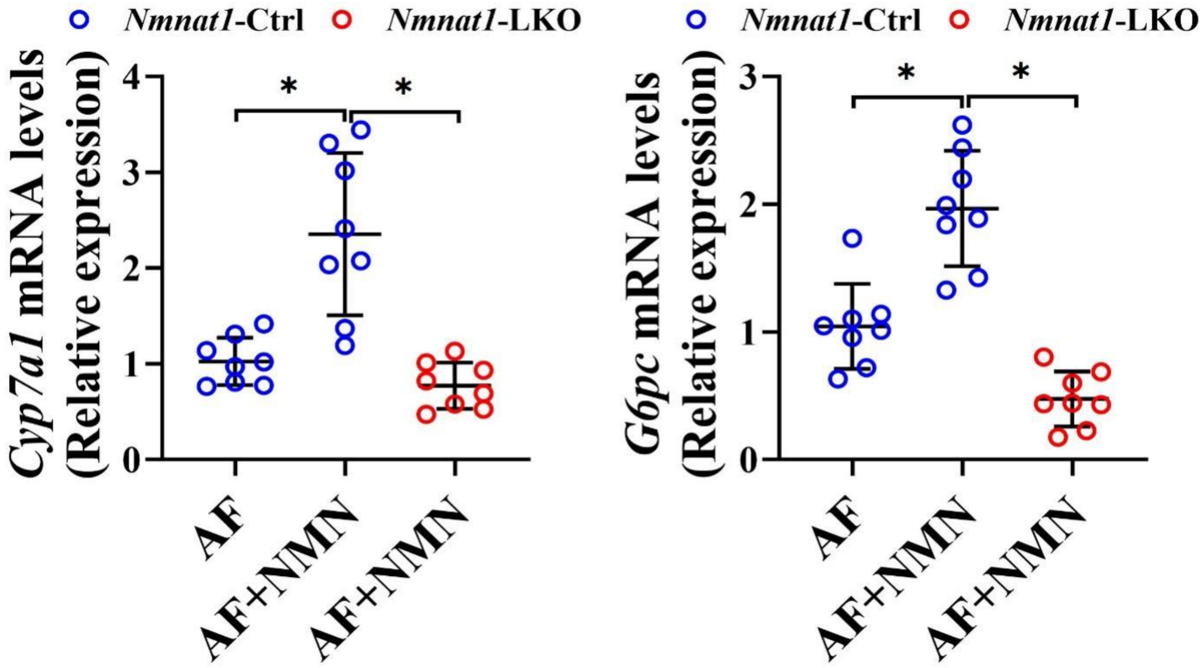

Figure S30. Hepatic *Nmnat1* knockout inhibited the ameliorative effect of NMN supplementation on the transcriptional activity of HNF4α in alcohol-fed mice. The expressions of *Cyp7a1* and *G6pc* mRNA were detected in the liver of alcohol-fed *Nmnat1*-Ctrl and *Nmnat1*-LKO mice supplemented with/without NMN (n = 8). Data are presented as means ± SD. *P < 0.05 represents statistical difference. AF, alcohol-fed; NMN, nicotinamide mononucleotide.

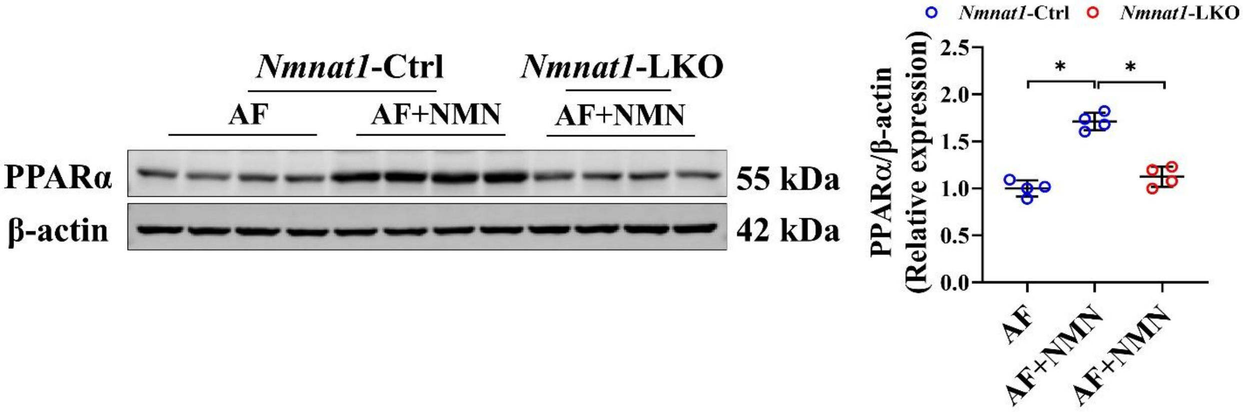

Figure S31. Hepatic *Nmnat1* knockout inhibited NMN-upregulated PPARα in mice liver. The PPARα **protein expression was detected in the liver of alcohol-fed *Nmnat1*-Ctrl and *Nmnat1*-LKO mice supplemented with/without NMN (n = 4). Protein band intensity was quantified by ImageJ**. Data are presented as means ± SD. **P* < 0.05 represents statistical difference. AF, alcohol-fed; NMN, nicotinamide mononucleotide.

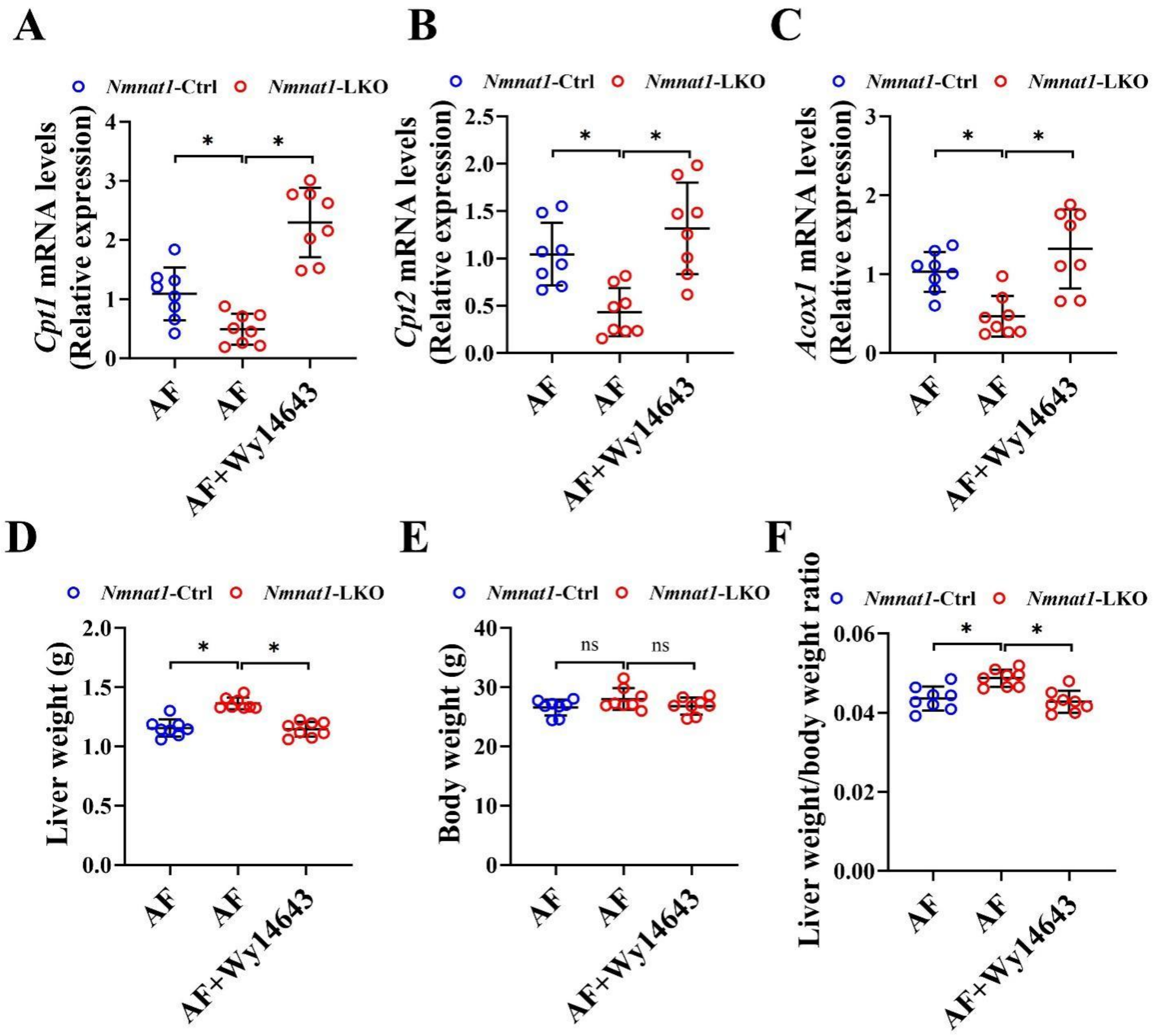

Figure S32. PPARα agonists (Wy-14643) intervention ameliorated chronic alcohol-induced increase of liver weight and its ratio to body weight in NMNAT1-LKO mice. (A-C) The activation efficiency of Wy-14643 on PPARα was evaluated by the detection of mRNA expressions of ***Cpt1*****,** ***Cpt2*****, and** ***Acox1*** **in mice liver (n = 8). (D) Liver weight (n = 8). (E) Body weight (n = 8). (F) Liver weight/body weight ratio (n = 8).** Data are presented as means ± SD. **P* < 0.05 represents statistical difference; ns represents no statistical difference. AF, alcohol-fed.

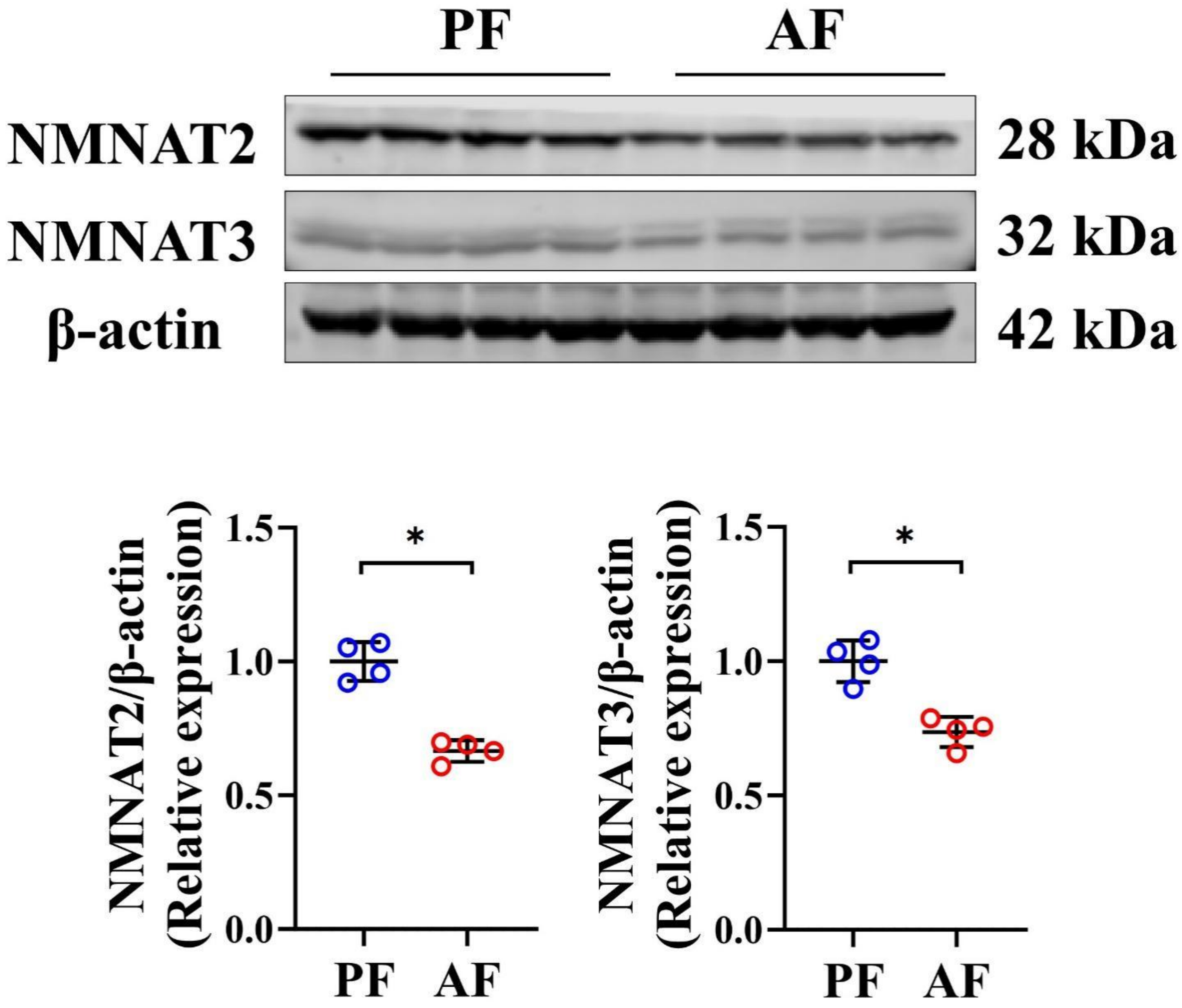

Figure S33. Chronic alcohol-fed decreased hepatic expressions of NMNAT2 and NMNAT3 in mice. The expressions of hepatic **NMNAT2 and NMNAT3 were detected in chronic alcohol-fed mice (n = 4). Protein band intensity was quantified by ImageJ.** Data are presented as means ± SD. **P* < 0.05 represents statistical difference. PF, pair-fed; AF, alcohol-fed.

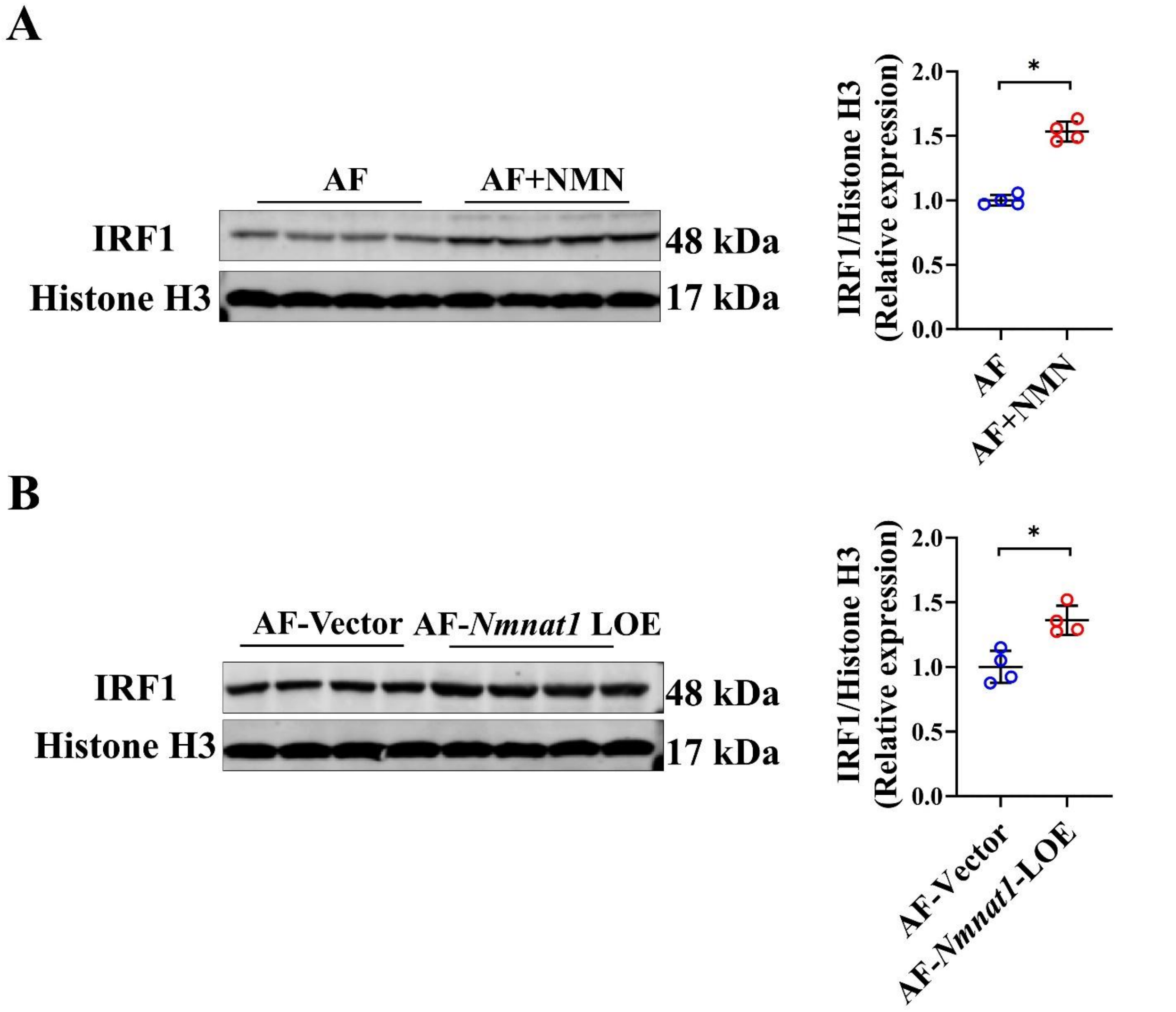

Figure S34. Nuclear IRF1 was upregulated in either NMN-administrated or Nmnat1-LOE mice liver. (A) **The nuclear** IRF1 **protein expression in the liver of alcohol-fed mice supplemented with/without NMN (n = 4). (B) The nuclear** IRF1 **protein expression in the liver of alcohol-fed** vector control and Nmnat1-LOE **mice (n = 4). Protein band intensity was quantified by ImageJ.** Data are presented as means ± SD. **P* < 0.05 represents statistical difference. AF, alcohol-fed; NMN, nicotinamide mononucleotide.

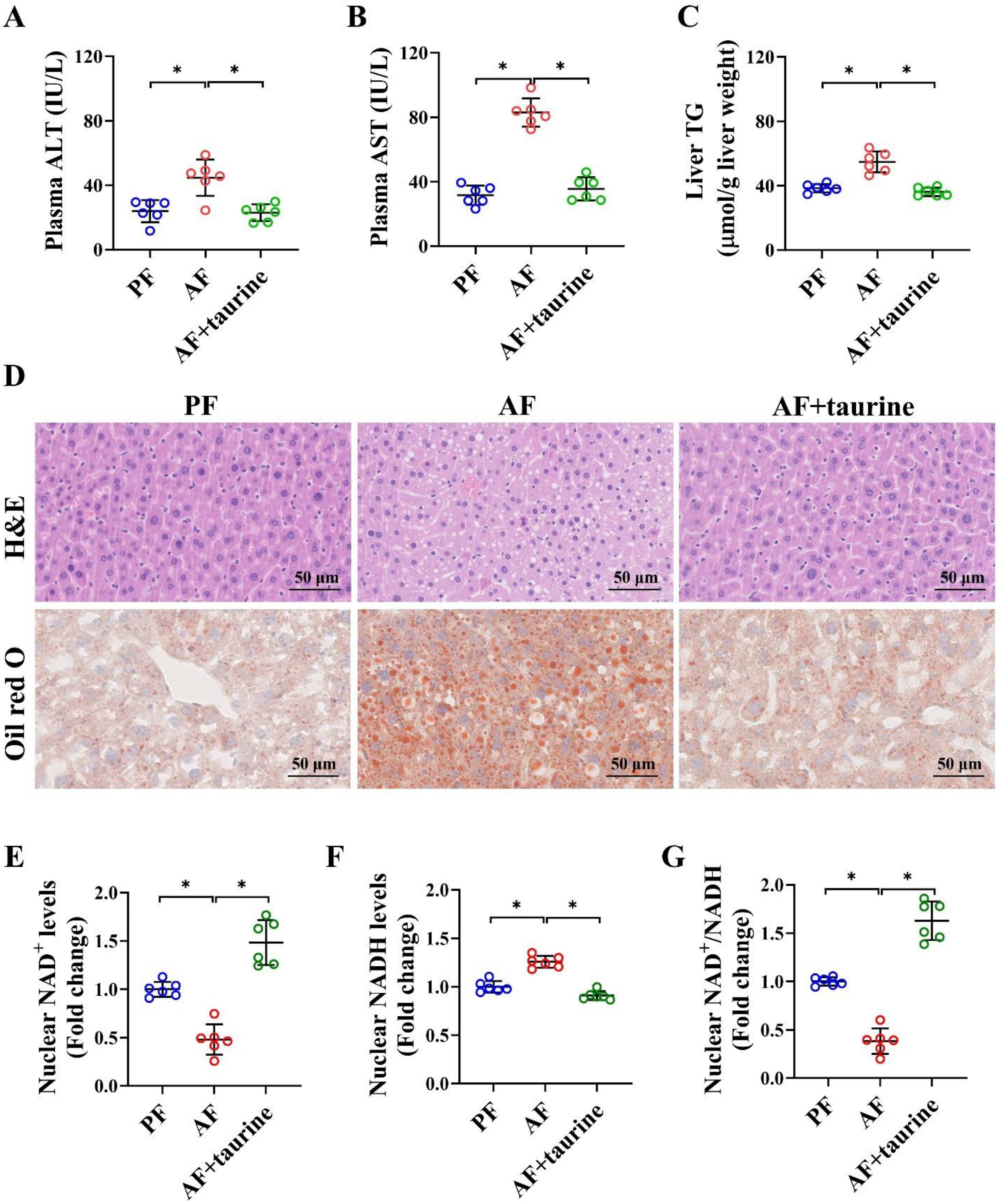

Figure S35. Taurine supplementation ameliorated chronic alcohol-induced liver injury and lipid deposition. (A) Plasma ALT levels (n = 6). (B) Plasma AST levels (n = 6). (C) Liver total triglyceride (TG) **levels (n = 6). (D & E)** Liver H&E and Oil red O staining **(n = 4). (E)**

Nuclear NAD^+ content (n = 6). (F) Nuclear NADH content (n = 6). (G) Nuclear NAD^+/NADH ratio (n = 6). Data are presented as means ± SD. *$P < 0.05$ represents statistical difference. PF, pair-fed; AF, alcohol-fed.

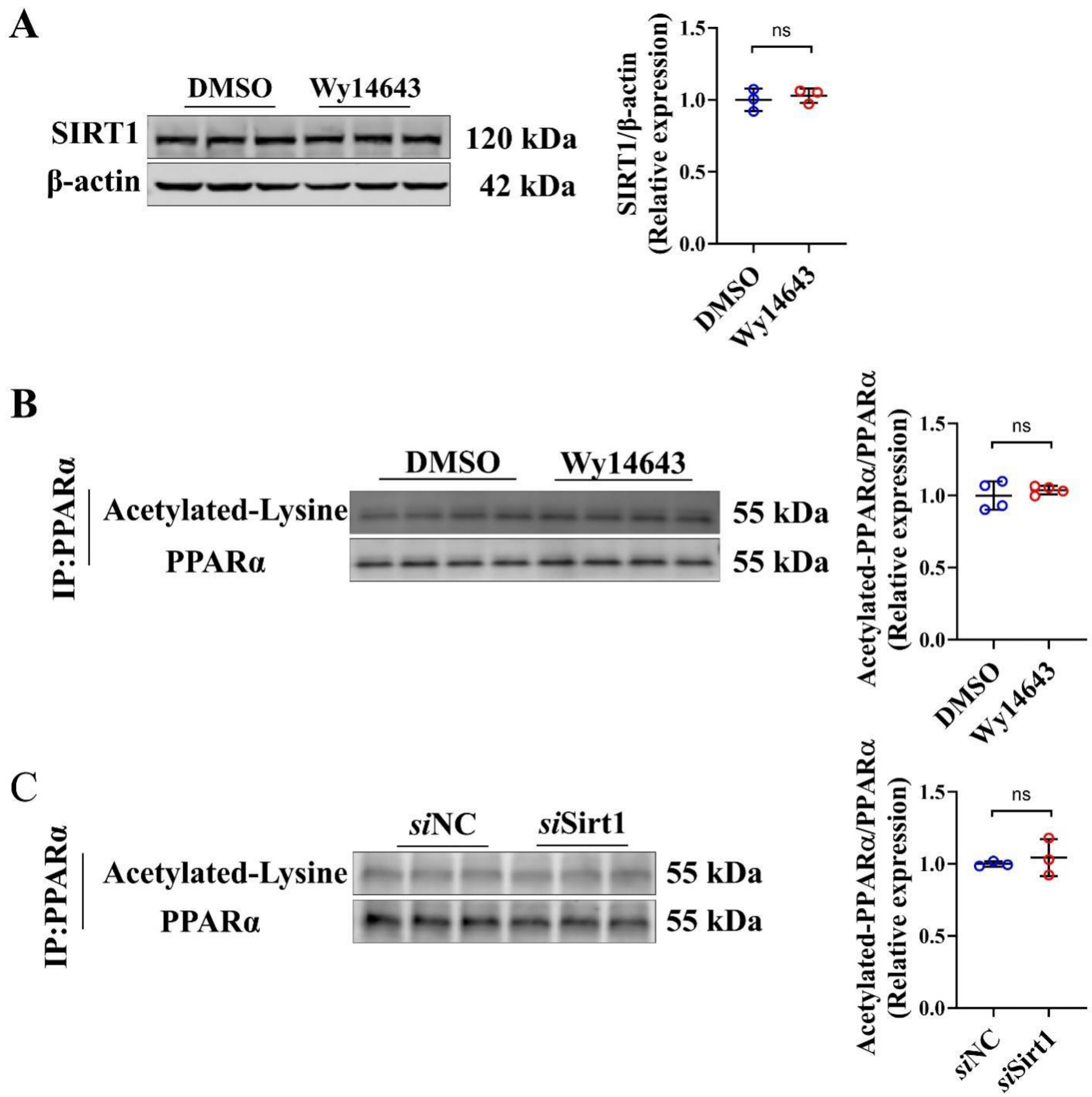

Figure S36. Neither SIRT1 expression nor PPARα acetylation **was regulated by Wy-14643 treatment. (A & B)** AML-12 **cells were treated with Wy-14643 (10 μM) for 24 h. SIRT1 protein (A) and acetylated-**PPARα **(B) expressions were measured by (n = 3-4). (C)** AML-12 **cells were transfected with either control siRNA (*si*NC) or SIRT1 siRNA (*si*SIRT1) for 48 h.** IP was performed to assess the acetylation level of PPARα**. (n = 3). Protein band intensity was quantified by ImageJ.** Data are presented as means ± SD. ns represents no statistical difference.

Supplementary Table 1. Primers designed for quantitative Real-Time PCR (mouse).

Gene name	Forward (5’-3’)	Reverse (5’-3’)
18S	AGGTCTGTGATGCCCTTAGA	GAATGGGGTTCAACGGGTTA
Nmnat1	TACGAGTCCGATGTGCTGTG	CCTTCGCTCTCCGTGTTGTA
Nmnat2	GATGTTCGAGAGAGCCAGGG	AAGGCCCTGTTTTCCGTAGG
Nmnat3	CCTGTGGTTCCTTCAACCCC	AGATGATGCCCTCAATCACCT
Irf1	GTTGTGCCATGAACTCCCTG	GTGTCCGGGCTAACATCTCC
Rarg	AAGTACACCACGAACTCCAGT	TTCGCAAACTCCACAATCTTGA
Mitf	CAAATGGCAAATACGTTACCCG	CAATGCTCTTGCTTCAGACTCT
Nos2	TACCAGATCGAGCCCTGGAAGA	AGCAAAGAACCACTTTCACCA
Eif2ak2	ATGCACGGAGTAGCCATTACG	TGACAATCCACCTTGTTTTCGT
Fbxw7	GTTCCGCTGCCTAATCTTCCT	CCCTTCAGGGATTCTGTGCC
Pparα	TGCCTTCCCTGTGAACTGAC	TGGGGAGAGAGGACAGATGG
Pgc1α	TATGGAGTGACATAGAGTGTGCT	CCACTTCAATCCACCCAGAAAG
Fasn	GGAGGTGGTGATAGCCGGTAT	TGGGTAATCCATAGAGCCCAG
Scd1	TTCTTGCGATACACTCTGGTGC	CGGGATTGAATGTTCTTGTCGT
Dgat1	GTGCCATCGTCTGCAAGATTC	GCATCACCACACACCAATTCAG
Dgat2	AGTGGCAATGCTATCATCATCGT	TCTTCTGGACCCATCGGCCCCAGGA
Cd36	GGACATTGAGATTCTTTTCCTCTG	GCAAAGGCATTGGCTGGAAGAAC
Vldlr	GAGTCTGACTTCGTGTGCAAA	GAACCGTCTTCGCAATCAGGA
Pnpla2	ATGTTCCCGAGGGAGACCAA	GAGGCTCCGTAGATGTGAGTG
Fatp5	CTACGCTGGCTGCATATAGATG	CCACAAAGGTCTCTGGAGGAT
Il-1β	GAAATGCCACCTTTTGACAGTG	TGGATGCTCTCATCAGGACAG

Cxcl1	ACTGCACCCAAACCGAAGTC	TGGGGACACCTTTTAGCATCTT
Tgf-β	TGCTAATGGTGGACCGCAA	CACTGCTTCCCGAATGTCTGA
Acta2	CTGACAGAGGCACCACTGAA	CATCTCCAGAGTCCAGCACA
Csad	CCAGGACGTGTTTGGGATTGT	ACCAGTCTTGACACTGTAGTGA
Fmo2	AGCATCTACCGCTCTGTCATT	CAGGAGTTTAGAGTTGTGCAGG
Fmo3	GGCCTGTGGAAATTCTCAGAC	AAGTCATCGGGATAGGGGAAG
Acox1	CCGCCACCTTCAATCCAGAG	CAAGTTCTCGATTTCTCGACGG
Cpt1	CTATGCGCTACTCGCTGAAGG	GGCTTTCGACCCGAGAAGA
Cpt2	CAAAAGACTCATCCGCTTTGTTC	CATCACGACTGGGTTTGGGTA
Cyp7a1	GAACCTCCTTTGGACAACGGG	GGAGTTTGTGATGAAGTGGACAT
G6pc	CGACTCGCTATCTCCAAGTGA	GGGCGTTGTCCAAACAGAAT
Atgl	GAGACCAAGTGGAACATC	GTAGATGTGAGTGGCGTT
Hsl	CCATCTCACCTCCCTTGG	TCCTTCCCGTAGGTCATAGG

Supplementary Table 2. Antibodies list.

Name	Supplier	Cat no.
NMNAT1	LSBio	LS-B12097, RRID: AB_3105945
NMNAT2	Santa Cruz Biotechnology	sc-515206, RRID: AB_2827765
NMNAT3	Santa Cruz Biotechnology	sc-390433, RRID: AB_2819344
IRF1	Proteintech	11335-1-AP, RRID: AB_2877759
MITF	Proteintech	13092-1-AP, RRID: AB_10597698
RARG	Immunoway	YN2233, AB_3626303
CSAD	Immunoway	YT7700, AB_3626311
PPAR-alpha	Santa Cruz Biotechnology	sc-398394, RRID: AB_2885073
HNF4 alpha	Affinity	AF6297, RRID: AB_2835147
Acetyl-HNF4 alpha (Lys106)	CUSABIO	CSB-PA727840, RRID: AB_3626302
Acetyl-FOXO1	Affinity	AF2305, RRID: AB_2845319
SIRT1	Santa Cruz Biotechnology	sc-74504, RRID: AB_2188348
FBXW7	Affinity	DF12400, RRID: AB_2845205
Acetylated-Lysine	Cell signaling technology	9441, RRID: AB_331805

CYP2E1	Proteintech	19937-1-AP, RRID: AB_10646444
ADH1	Santa Cruz Biotechnology	sc-133207, RRID：AB_2223714
ALDH2	Santa Cruz Biotechnology	sc-100496, RRID：AB_2242451
Lamin B1	Proteintech	12987-1-AP, RRID: AB_2136290
Histone H3	Huabio	HA500298, RRID: AB_3071393
β-actin	ABclonal	AC026, RRID: AB_2768234
IRDye® 680RD Goat anti-Rabbit IgG Secondary Antibody	Licor	P/N 925-68071, RRID: AB_2721181
IRDye® 680RD Goat anti-Mouse IgG Secondary Antibody	Licor	P/N 925-68070, RRID: AB_2651128

Data S1.
Raw data of Figures and supplementary figures

REFERENCES AND NOTES

1. H. K. Seitz, R. Bataller, H. Cortez-Pinto, B. Gao, A. Gual, C. Lackner, P. Mathurin, S. Mueller, G. Szabo, H. Tsukamoto, Alcoholic liver disease. *Nat. Rev. Dis. Primers.* **4**, 16 (2018).

2. B. Mackowiak, Y. Fu, L. Maccioni, B. Gao, Alcohol-associated liver disease. *J. Clin. Invest.* **134**, e176345 (2024).

3. A. J. Covarrubias, R. Perrone, A. Grozio, E. Verdin, NAD^+ metabolism and its roles in cellular processes during ageing. *Nat. Rev. Mol. Cell Biol.* **22**, 119–141 (2021).

4. S. W. French, Chronic alcohol binging injures the liver and other organs by reducing NAD^+ levels required for sirtuin's deacetylase activity. *Exp. Mol. Pathol.* **100**, 303–306 (2016).

5. L. Hao, Q. Sun, W. Zhong, W. Zhang, X. Sun, Z. Zhou, Mitochondria-targeted ubiquinone (MitoQ) enhances acetaldehyde clearance by reversing alcohol-induced posttranslational modification of aldehyde dehydrogenase 2: A molecular mechanism of protection against alcoholic liver disease. *Redox Biol.* **14**, 626–636 (2018).

6. S. Wang, T. Wan, M. Ye, Y. Qiu, L. Pei, R. Jiang, N. Pang, Y. Huang, B. Liang, W. Ling, X. Lin, Z. Zhang, L. Yang, Nicotinamide riboside attenuates alcohol induced liver injuries via activation of SirT1/PGC-1α/mitochondrial biosynthesis pathway. *Redox Biol.* **17**, 89–98 (2018).

7. Y. Liu, C. Cheng, H. Gao, X. J. Zhu, X. He, M. X. Zhou, Y. Gao, Y. W. Lu, X. H. Song, X. H. Xiao, J. B. Wang, C. J. Xu, Z. T. Ma, Restoring energy metabolism by NAD^+ supplement prevents alcohol-induced liver injury and boosts liver regeneration. *Food Sci. Nutr.* **12**, 5100–5110 (2024).

8. N. M. Gold, Q. Ding, Y. Yang, S. Pu, W. Cao, X. Ge, P. Yang, M. N. Okeke, A. Nisar, Y. Pan, Q. Luo, X. Wang, H. Xu, R. Tian, M. Zi, X. Zhang, S. Li, Y. He, Therapeutic potential of nicotinamide and ABT263 in alcohol-associated liver disease through targeting cellular senescence. *MedComm* **6**, e70086 (2025).

9. J. M. Brazill, C. Li, Y. Zhu, R. G. Zhai, NMNAT: It's an NAD$^+$ synthase... It's a chaperone... It's a neuroprotector. *Curr. Opin. Genet. Dev.* **44**, 156–162 (2017).

10. V. Mori, A. Amici, F. Mazzola, M. Di Stefano, L. Conforti, G. Magni, S. Ruggieri, N. Raffaelli, G. Orsomando, Metabolic profiling of alternative NAD biosynthetic routes in mouse tissues. *PLOS ONE* **9**, e113939 (2014).

11. M. Emanuelli, F. Carnevali, F. Saccucci, F. Pierella, A. Amici, N. Raffaelli, G. Magni, Molecular cloning, chromosomal localization, tissue mRNA levels, bacterial expression, and enzymatic properties of human NMN adenylyltransferase. *J. Biol. Chem.* **276**, 406–412 (2001).

12. L. Sorci, F. Cimadamore, S. Scotti, R. Petrelli, L. Cappellacci, P. Franchetti, G. Orsomando, G. Magni, Initial-rate kinetics of human NMN-adenylyltransferases: substrate and metal ion specificity, inhibition by products and multisubstrate analogues, and isozyme contributions to NAD$^+$ biosynthesis. *Biochemistry* **46**, 4912–4922 (2007).

13. L. Conforti, L. Janeckova, D. Wagner, F. Mazzola, L. Cialabrini, M. Di Stefano, G. Orsomando, G. Magni, C. Bendotti, N. Smyth, M. Coleman, Reducing expression of NAD$^+$ synthesizing enzyme NMNAT1 does not affect the rate of Wallerian degeneration. *FEBS J.* **278**, 2666–2679 (2011).

14. S. H. Lee, M. Nishino, T. Mazumdar, G. E. Garcia, M. Galfione, F. L. Lee, C. L. Lee, A. Liang, J. Kim, L. Feng, N. T. Eissa, S. H. Lin, L. Y. Yu-Lee, 16-kDa prolactin down-regulates inducible nitric oxide synthase expression through inhibition of the signal transducer and activator of transcription 1/IFN regulatory factor-1 pathway. *Cancer Res.* **65**, 7984–7992 (2005).

15. T. Sato, C. Selleri, N. S. Young, J. P. Maciejewski, Inhibition of interferon regulatory factor-1 expression results in predominance of cell growth stimulatory effects of interferon-gamma due to phosphorylation of Stat1 and Stat3. *Blood* **90**, 4749–4758 (1997).

16. M. Galicia-Moreno, G. Gutierrez-Reyes, The role of oxidative stress in the development of alcoholic liver disease. *Rev. Gastroenterol. Mex.* **79**, 135–144 (2014).

17. A. J. Garvin, A. H. A. Khalaf, A. Rettino, J. Xicluna, L. Butler, J. R. Morris, D. M. Heery, N. M. Clarke, GSK3β-SCFFBXW7α mediated phosphorylation and ubiquitination of IRF1 are required for its transcription-dependent turnover. *Nucleic Acids Res.* **47**, 4476–4494 (2019).

18. W. Zhong, Y. Zhao, Y. Tang, X. Wei, X. Shi, W. Sun, X. Sun, X. Yin, X. Sun, S. Kim, C. J. McClain, X. Zhang, Z. Zhou, Chronic alcohol exposure stimulates adipose tissue lipolysis in mice: role of reverse triglyceride transport in the pathogenesis of alcoholic steatosis. *Am. J. Pathol.* **180**, 998–1007 (2012).

19. Y. Wang, D. Matye, N. Nguyen, Y. Zhang, T. Li, HNF4α regulates CSAD to couple hepatic taurine production to bile acid synthesis in mice. *Gene Expr.* **18**, 187–196 (2018).

20. H. Dong, W. Guo, R. Yue, X. Sun, Z. Zhou, Nuclear nicotinamide adenine dinucleotide deficiency by Nmnat1 deletion impaired hepatic insulin signaling, mitochondrial function, and hepatokine expression in mice fed a high-fat diet. *Lab. Invest.* **104**, 100329 (2024).

21. Y. Sasaki, B. P. Vohra, F. E. Lund, J. Milbrandt, Nicotinamide mononucleotide adenylyl transferase-mediated axonal protection requires enzymatic activity but not increased levels of neuronal nicotinamide adenine dinucleotide. *J. Neurosci.* **29**, 5525–5535 (2009).

22. H. Yin, M. Hu, X. Liang, J. M. Ajmo, X. Li, R. Bataller, G. Odena, S. M. Stevens, Jr., M. You, Deletion of SIRT1 from hepatocytes in mice disrupts lipin-1 signaling and aggravates alcoholic fatty liver. *Gastroenterology* **146**, 801–811 (2014).

23. Y. Yang, H. Hou, E. M. Haller, S. V. Nicosia, W. Bai, Suppression of FOXO1 activity by FHL2 through SIRT1-mediated deacetylation. *EMBO J.* **24**, 1021–1032 (2005).

24. Q. Song, Y. Chen, J. Wang, L. Hao, C. Huang, A. Griffiths, Z. Sun, Z. Zhou, Z. Song, ER stress-induced upregulation of NNMT contributes to alcohol-related fatty liver development. *J. Hepatol.* **73**, 783–793 (2020).

25. X. Xiong, J. Yu, R. Fan, C. Zhang, L. Xu, X. Sun, Y. Huang, Q. Wang, H. B. Ruan, X. Qian, NAMPT overexpression alleviates alcohol-induced hepatic steatosis in mice. *PLOS ONE* **14**, e0212523 (2019).

26. W. Zhong, W. Zhang, Q. Li, G. Xie, Q. Sun, X. Sun, X. Tan, X. Sun, W. Jia, Z. Zhou, Pharmacological activation of aldehyde dehydrogenase 2 by Alda-1 reverses alcohol-induced hepatic steatosis and cell death in mice. *J. Hepatol.* **62**, 1375–1381 (2015).

27. Y. Zhang, C. Wang, Y. Tian, F. Zhang, W. Xu, X. Li, Z. Shu, Y. Wang, K. Huang, D. Huang, Inhibition of Poly(ADP-Ribose) polymerase-1 protects chronic alcoholic liver injury. *Am. J. Pathol.* **186**, 3117–3130 (2016).

28. D. Sokolov, E. R. Sechrest, Y. Wang, C. Nevin, J. Du, S. Kolandaivelu, Nuclear NAD^+-biosynthetic enzyme NMNAT1 facilitates development and early survival of retinal neurons. *eLife* **10**, e71185 (2021).

29. F. Rossi, P. C. Geiszler, W. Meng, M. R. Barron, M. Prior, A. Herd-Smith, A. Loreto, M. Y. Lopez, H. Faas, M. C. Pardon, L. Conforti, NAD-biosynthetic enzyme NMNAT1 reduces early behavioral impairment in the htau mouse model of tauopathy. *Behav. Brain Res.* **339**, 140–152 (2018).

30. P. Wang, Y. Lu, D. Han, P. Wang, L. Ren, J. Bi, J. Liang, Neuroprotection by nicotinamide mononucleotide adenylyltransferase 1 with involvement of autophagy in an aged rat model of transient cerebral ischemia and reperfusion. *Brain Res.* **1723**, 146391 (2019).

31. Y. Zhang, X. Guo, Z. Peng, C. Liu, L. Ren, J. Liang, P. Wang, Nicotinamide mononucleotide adenylyltransferase 1 regulates cerebral ischemia-induced blood-brain barrier disruption through NAD^+/SIRT1 signaling pathway. *Mol. Neurobiol.* **59**, 4879–4891 (2022).

32. F. Cheng, Y. Zhang, H. Xiong, M. Zhao, Q. Wang, Y. Zhu, Y. Li, R. Tang, J. Li, NMNATs expression inhibition mediated NAD^+ deficiency plays a critical role in doxorubicin-induced hepatotoxicity in mice. *Toxicol. Appl. Pharmacol.* **482**, 116799 (2024).

33. T. Iqbal, A. Nawaz, M. Karim, K. Yaku, K. Hikosaka, M. Matsumoto, T. Nakagawa, Loss of hepatic Nmnat1 has no impact on diet-induced fatty liver disease. *Biochem. Biophys. Res. Commun.* **636**, 89–95 (2022).

34. M. Dall, M. Penke, K. Sulek, M. Matz-Soja, B. Holst, A. Garten, W. Kiess, J. T. Treebak, Hepatic NAD^+ levels and NAMPT abundance are unaffected during prolonged high-fat diet consumption in C57BL/6JBomTac mice. *Mol. Cell. Endocrinol.* **473**, 245–256 (2018).

35. X. Wei, C. Wei, Y. Tan, X. Dong, Z. Yang, J. Yan, X. Luo, Both prolonged high-fat diet consumption and calorie restriction boost hepatic NAD^+ metabolism in mice. *J. Nutr. Biochem.* **115**, 109296 (2023).

36. S. Yamaguchi, D. Kojima, T. Iqbal, S. Kosugi, M. P. Franczyk, N. Qi, Y. Sasaki, K. Yaku, K. Kaneko, K. Kinouchi, H. Itoh, K. Hayashi, T. Nakagawa, J. Yoshino, Adipocyte NMNAT1 expression is essential for nuclear NAD^+ biosynthesis but dispensable for regulating thermogenesis and whole-body energy metabolism. *Biochem. Biophys. Res. Commun.* **674**, 162–169 (2023).

37. M. Karim, T. Iqbal, A. Nawaz, K. Yaku, T. Nakagawa, Deletion of Nmnat1 in skeletal muscle leads to the reduction of NAD^+ levels but has no impact on skeletal muscle morphology and fiber types. *J. Nutr. Sci. Vitaminol.* **69**, 184–189 (2023).

38. X. A. Cambronne, M. L. Stewart, D. Kim, A. M. Jones-Brunette, R. K. Morgan, D. L. Farrens, M. S. Cohen, R. H. Goodman, Biosensor reveals multiple sources for mitochondrial NAD^+. *Science* **352**, 1474–1477 (2016).

39. A. Guldenpfennig, A. K. Hopp, L. Muskalla, P. Manetsch, F. Raith, L. Hellweg, C. Dordelmann, D. M. Leslie Pedrioli, K. Johnsson, G. Superti-Furga, M. O. Hottiger, Absence of mitochondrial SLC25A51 enhances PARP1-dependent DNA repair by increasing nuclear NAD^+ levels. *Nucleic Acids Res.* **51**, 9248–9265 (2023).

40. J. Li, X. Y. Cheng, R. X. Ma, B. Zou, Y. Zhang, M. M. Wu, Y. Yao, J. Li, Nicotinamide mononucleotide combined with PJ-34 protects microglial cells from lipopolysaccharide-induced mitochondrial impairment through NMNAT3-PARP1 axis. *J. Transl. Med.* **23**, 279 (2025).

41. H. Negishi, T. Taniguchi, H. Yanai, The interferon (IFN) class of cytokines and the IFN regulatory factor (IRF) transcription factor family. *Cold Spring Harb. Perspect. Biol.* **10**, a028423 (2018).

42. H. Feng, Y. B. Zhang, J. F. Gui, S. M. Lemon, D. Yamane, Interferon regulatory factor 1 (IRF1) and anti-pathogen innate immune responses. *PLOS Pathog.* **17**, e1009220 (2021).

43. K. Gao, Q. Shi, Y. Gu, W. Yang, Y. He, Z. Lv, Y. Ding, W. Cao, C. Wang, X. Wan, SPOP mutations promote tumor immune escape in endometrial cancer via the IRF1-PD-L1 axis. *Cell Death Differ.* **30**, 475–487 (2023).

44. G. Jing, Y. Ma, OGT-mediated O-GlcNAcylation regulates macrophage polarization in heart failure via targeting IRF1. *BMC Cardiovasc. Disord.* **24**, 757 (2024).

45. S. L. Stevens, P. Y. Leung, K. B. Vartanian, B. Gopalan, T. Yang, R. P. Simon, M. P. Stenzel-Poore, Multiple preconditioning paradigms converge on interferon regulatory factor-dependent signaling to promote tolerance to ischemic brain injury. *J. Neurosci.* **31**, 8456–8463 (2011).

46. C. Zeng, X. Zhu, H. Li, Z. Huang, M. Chen, The role of interferon regulatory factors in liver diseases. *Int. J. Mol. Sci.* **25**, e176345 (2024).

47. S. Liang, Z. Zhong, S. Y. Kim, R. Uchiyama, Y. S. Roh, H. Matsushita, R. A. Gottlieb, E. Seki, Murine macrophage autophagy protects against alcohol-induced liver injury by degrading interferon regulatory factor 1 (IRF1) and removing damaged mitochondria. *J. Biol. Chem.* **294**, 12359–12369 (2019).

48. H. Li, X. Chen, J. Xu, L. Zhu, C. Li, X. Sun, X. Li, J. Guo, J. Li, S. Wang, Y. He, H. Wang, C. Huang, X. M. Meng, J. Li, GRP/GRPR enhances alcohol-associated liver injury through the IRF1-mediated Caspase-1 inflammasome and NOX2-dependent ROS pathway. *Hepatology* **79**, 392–408 (2024).

49. Y. Lu, A. I. Cederbaum, CYP2E1 and oxidative liver injury by alcohol. *Free Radic. Biol. Med.* **44**, 723–738 (2008).

50. X. Dou, S. Li, Z. Wang, D. Gu, C. Shen, T. Yao, Z. Song, Inhibition of NF-κB activation by 4-hydroxynonenal contributes to liver injury in a mouse model of alcoholic liver disease. *Am. J. Pathol.* **181**, 1702–1710 (2012).

51. J. Y. Wu, X. W. Tang, J. V. Schloss, M. D. Faiman, Regulation of taurine biosynthesis and its physiological significance in the brain. *Adv. Exp. Med. Biol.* **442**, 339–345 (1998).

52. Y. C. Chang, S. T. Ding, Y. H. Lee, Y. C. Wang, M. F. Huang, I. H. Liu, Taurine homeostasis requires de novo synthesis via cysteine sulfinic acid decarboxylase during zebrafish early embryogenesis. *Amino Acids* **44**, 615–629 (2013).

53. R. Tan, J. Li, L. Liu, Q. Wu, L. Fan, N. Ma, C. Yu, H. Lu, X. Zhang, J. Chen, L. Gong, J. Ren, CSAD ameliorates lipid accumulation in high-fat diet-fed mice. *Int. J. Mol. Sci.* **23**, 15931(2022).

54. R. Tang, Q. Yang, S. Lin, Y. Feng, J. Yang, Q. Lv, G. Wu, J. Hu, Preventive or curative administration of taurine regulates lipid metabolism in the liver of rats with alcoholic liver disease. *Adv. Exp. Med. Biol.* **1155**, 119–131 (2019).

55. C. J. Lin, C. C. Chiu, Y. C. Chen, M. L. Chen, T. C. Hsu, B. S. Tzang, Taurine attenuates hepatic inflammation in chronic alcohol-fed rats through inhibition of TLR4/MyD88 signaling. *J. Med. Food* **18**, 1291–1298 (2015).

56. L. Xiao, G. F. Xu, S. L. Chen, Y. M. He, F. Peng, C. F. Yuan, Kaempferol ameliorated alcoholic liver disease through inhibiting hepatic bile acid synthesis by targeting intestinal FXR-FGF15 signaling. *Phytomedicine* **120**, 155055 (2023).

57. Y. J. Fang, C. H. Chiu, Y. Y. Chang, C. H. Chou, H. W. Lin, M. F. Chen, Y. C. Chen, Taurine ameliorates alcoholic steatohepatitis via enhancing self-antioxidant capacity and alcohol metabolism. *Food Res. Int.* **44**, 3105–3110 (2011).

58. A. Kiss, C. Csikos, Z. Regdon, Z. Polgar, L. Virag, C. Hegedus, NMNAT1 is a survival factor in actinomycin D-induced osteosarcoma cell death. *Int. J. Mol. Sci.* **22**, 8869 (2021).

59. T. Song, L. Yang, N. Kabra, L. Chen, J. Koomen, E. B. Haura, J. Chen, The NAD^+ synthesis enzyme nicotinamide mononucleotide adenylyltransferase (NMNAT1) regulates ribosomal RNA transcription. *J. Biol. Chem.* **288**, 20908–20917 (2013).

60. T. Zhang, J. G. Berrocal, K. M. Frizzell, M. J. Gamble, M. E. DuMond, R. Krishnakumar, T. Yang, A. A. Sauve, W. L. Kraus, Enzymes in the NAD^+ salvage pathway regulate SIRT1 activity at target gene promoters. *J. Biol. Chem.* **284**, 20408–20417 (2009).

61. M. You, A. Jogasuria, C. Taylor, J. Wu, Sirtuin 1 signaling and alcoholic fatty liver disease. *Hepatobiliary Surg. Nutr.* **4**, 88–100 (2015).

62. J. Argemi, M. U. Latasa, S. R. Atkinson, I. O. Blokhin, V. Massey, J. P. Gue, J. Cabezas, J. J. Lozano, D. Van Booven, A. Bell, S. Cao, L. A. Vernetti, J. P. Arab, M. Ventura-Cots, L. R. Edmunds, C. Fondevila, P. Starkel, L. Dubuquoy, A. Louvet, G. Odena, J. L. Gomez, T. Aragon, J. Altamirano, J. Caballeria, M. J. Jurczak, D. L. Taylor, C. Berasain, C. Wahlestedt, S. P. Monga, M. Y. Morgan, P. Sancho-Bru, P. Mathurin, S. Furuya, C. Lackner, I. Rusyn, V. H. Shah, M. R. Thursz, J. Mann, M. A. Avila, R. Bataller, Author correction: Defective HNF4alpha-dependent gene expression as a driver of hepatocellular failure in alcoholic hepatitis. *Nat. Commun.* **14**, 757 (2023).

63. F. Liu, Y. Peng, H. Qian, M. C. Xiao, C. H. Ding, X. Zhang, W. F. Xie, Abrogating K458 acetylation enhances hepatocyte nuclear factor 4α (HNF4α)-induced differentiation therapy for hepatocellular carcinoma. *J. Dig. Dis.* **25**, 255–265 (2024).

64. H. Ren, F. Hu, D. Wang, X. Kang, X. Feng, L. Zhang, B. Zhou, S. Liu, G. Yuan, Sirtuin 2 prevents liver steatosis and metabolic disorders by deacetylation of hepatocyte nuclear factor 4α. *Hepatology* **74**, 723–740 (2021).

65. A. Yokoyama, S. Katsura, R. Ito, W. Hashiba, H. Sekine, R. Fujiki, S. Kato, Multiple post-translational modifications in hepatocyte nuclear factor 4α. *Biochem. Biophys. Res. Commun.* **410**, 749–753 (2011).

66. R. A. Palu, C. S. Thummel, Sir2 acts through hepatocyte nuclear factor 4 to maintain insulin signaling and metabolic homeostasis in *Drosophila*. *PLOS Genet.* **12**, e1005978 (2016).

67. F. G. Meng, X. N. Zhang, S. X. Liu, Y. R. Wang, T. Zeng, Roles of peroxisome proliferator-activated receptor α in the pathogenesis of ethanol-induced liver disease. *Chem. Biol. Interact.* **327**, 109176 (2020).

68. J. H. Suh, K. H. Kim, M. E. Conner, D. D. Moore, G. A. Preidis, Hepatic PPARα is destabilized by SIRT1 deacetylase in undernourished male mice. *Front. Nutr.* **9**, 831879 (2022).

69. Y. Y. Chang, C. H. Chou, C. H. Chiu, K. T. Yang, Y. L. Lin, W. L. Weng, Y. C. Chen, Preventive effects of taurine on development of hepatic steatosis induced by a high-fat/cholesterol dietary habit. *J. Agric. Food Chem.* **59**, 450–457 (2011).

70. M. L. Bonfleur, P. C. Borck, R. A. Ribeiro, L. C. Caetano, G. M. Soares, E. M. Carneiro, S. L. Balbo, Improvement in the expression of hepatic genes involved in fatty acid metabolism in obese rats supplemented with taurine. *Life Sci.* **135**, 15–21 (2015).

71. Z. Sadr, A. Ghasemi, M. Rohani, A. Alavi, NMNAT1 and hereditary spastic paraplegia (HSP): Expanding the phenotypic spectrum of NMNAT1 variants. *Neuromuscul. Disord.* **33**, 295–301 (2023).

72. A. Eblimit, S. A. Zaneveld, W. Liu, K. Thomas, K. Wang, Y. Li, G. Mardon, R. Chen, NMNAT1 E257K variant, associated with Leber Congenital Amaurosis (LCA9), causes a mild retinal degeneration phenotype. *Exp. Eye Res.* **173**, 32–43 (2018).

73. N. Bedoni, M. Quinodoz, M. Pinelli, G. Cappuccio, A. Torella, V. Nigro, F. Testa, F. Simonelli, TUDP (Telethon Undiagnosed Disease Program), M. Corton, S. Lualdi, F. Lanza, G. Morana, C. Ayuso, M. D. Rocco, M. Filocamo, S. Banfi, N. Brunetti-Pierri, A. Superti-Furga, C. Rivolta, An Alu-mediated duplication in NMNAT1, involved in NAD biosynthesis, causes a novel syndrome, SHILCA, affecting multiple tissues and organs. *Hum. Mol. Genet.* **29**, 2250–2260 (2020).

74. J. Qiu, F. Dong, H. Zhuge, Q. Han, J. Li, R. Guo, X. Dou, J. Li, S. Li, Preventive effect of low-carbohydrate high-fat dietary pattern on liver disease caused by alcohol consumption via a 6pgd-involved mechanism in mice. *Food Funct.* **15**, 732–746 (2024).

75. W. Zhong, X. Wei, L. Hao, T. D. Lin, R. Yue, X. Sun, W. Guo, H. Dong, T. Li, A. R. Ahmadi, Z. Sun, Q. Zhang, J. Zhao, Z. Zhou, Paneth cell dysfunction mediates alcohol-related steatohepatitis through promoting bacterial translocation in mice: Role of zinc deficiency. *Hepatology* **71**, 1575–1591 (2020).

76. N. Huda, P. Kusumanchi, K. Perez, Y. Jiang, N. J. Skill, Z. Sun, J. Ma, Z. Yang, S. Liangpunsakul, Telomere length in patients with alcohol-associated liver disease: A brief report. *J. Invest. Med.* **70**, 1438–1441 (2022).

77. Q. Ding, A. Pi, L. Hao, T. Xu, Q. Zhu, L. Shu, X. Yu, W. Wang, C. Si, S. Li, Genistein protects against acetaldehyde-induced oxidative stress and hepatocyte injury in chronic alcohol-fed mice. *J. Agric. Food Chem.* **71**, 1930–1943 (2023).

78. Q. Ding, R. Guo, L. Hao, Q. Song, A. Fu, S. Lai, T. Xu, H. Zhuge, K. Chang, Y. Chen, H. Wei, D. Ren, Z. Sun, Z. Song, X. Dou, S. Li, Hepatic TRPC3 loss contributes to chronic alcohol consumption-induced hepatic steatosis and liver injury in mice. *Life Metab.* **3**, load050 (2024)

79. T. M. Donohue, N. A. Osna, D. L. Clemens, Recombinant Hep G2 cells that express alcohol dehydrogenase and cytochrome P450 2E1 as a model of ethanol-elicited cytotoxicity. *Int. J. Biochem. Cell Biol.* **38**, 92–101 (2006).

80. Q. Sun, W. Zhang, W. Zhong, X. Sun, Z. Zhou, Dietary fisetin supplementation protects against alcohol-induced liver injury in mice. *Alcohol. Clin. Exp. Res.* **40**, 2076–2084 (2016).
